# Origin and Evolution of RAS Oncoprotein Membrane Targeting

**DOI:** 10.21203/rs.3.rs-2485219/v1

**Published:** 2023-01-20

**Authors:** Antonio García-España, Mark R. Philips

**Affiliations:** * Bionos Biotech SL; Biopolo Hospital La Fe, Valencia, Spain; # Perlmutter Cancer Center, New York University Grossman School of Medicine, New York, NY, USA

## Abstract

*KRAS, HRAS* and *NRAS* oncogenes belong to a family of 40 highly homologous genes, which in turn are a subset of a superfamily of >160 genes encoding small GTPases. RAS oncoproteins consist of a globular G-domain (aa1-166) and a 22-23aa unstructured hypervariable region (HVR) that mediates membrane targeting. The evolutionarily origins of the RAS isoforms, their HVRs and alternative splicing of the *KRAS* locus has not been explored. We found that *KRAS* is basal to the oncogene family and its duplication generated *HRAS* in the common ancestor of vertebrates. In a second round of duplication *HRAS* generated *NRAS and KRAS* generated an additional *RAS* gene we have designated *KRASBL*, absent in mammals and birds. KRAS4A arose through a duplication and insertion of the 4^th^ exon of *NRAS* into the 3^rd^ intron of *KRAS*. We found evolutionarily conservation of a short polybasic region (PBR1) in HRAS, NRAS and KRAS4A, a second polybasic region (PBR2) in KRAS4A, two neutralized basic residues (NB) and a serine in KRAS4B and KRASBL, and a modification of the CaaX motif in vertebrates with farnesyl rather than geranylgeranyl polyisoprene lipids, suggesting that a less hydrophobic membrane anchor is critical to RAS oncoprotein function. The persistence of four RAS isoforms through >400 MY of evolution argues strongly for differential function.

## INTRODUCTION

RAS oncoproteins, HRAS, NRAS, KRAS4B and KRAS4A have been extensively studied due to their frequent activation in human tumors [1, 2]. Oncogenic mutations in their catalytic domains, particularly at residues G12, G13, and Q61, are among the most common in mammalian tumors [3].

At the sequence level, RAS oncoproteins consist of two functionally distinct regions: (i) catalytic GTP/GDP binding domains (G-domain) comprising residues 1-166, which are very similar between isoforms (G-domains amino acids are 90% identical within human isoforms) and fold into a well-characterized protein domains that acts as binary molecular switches, and (ii) an intrinsically disordered C-terminal hypervariable region (HVR) of 22-23 residues that anchors the GTPases to cellular membranes [4, 5]. G-domains are highly stable structures with important functional elements including a set of five conserved G box GTP/GDP binding elements and two switch-regions (I and II) that form the binding interface for guanine nucleotide exchange factors (GEFs), effectors, and GTPase activating proteins (GAPs) [6].

**Fig. 1.**
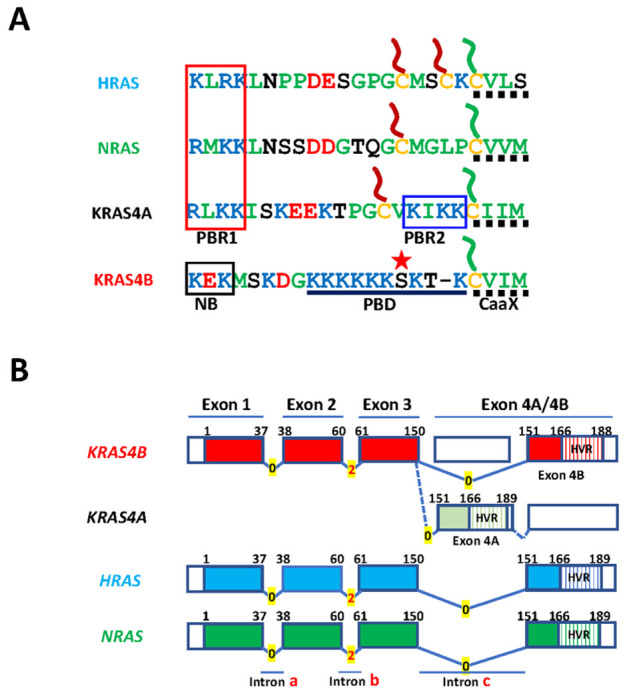
Membrane targeting motifs and gene structure of human RAS oncoproteins. (A) Hypervariable regions (HVR) that contain membrane targeting motifs. The ubiquitous C-terminal CaaX motif (dotted line) is shown with its cysteine (yellow) modified with a farnesyl lipid (green). The other hVr cysteines are shown modified with palmitate (red). The strong polybasic domain (PBD) of KRAS4B is shown with lysines in blue with an intervening serine 181 starred to indicate the phosphorylation site. Polybasic region 1 (PBR1) is shared by HRAS, NRAS and KRAS4A. Polybasic region 2 (PBR2) is unique to KRAS4A. A neutralized basic motif (NB) is boxed in KRAS4B. (B) Splicing of *RAS* oncogene transcripts. Coding exons are depicted as boxes numbered 1-4 and connecting introns a, b, and c as lines with intron phases 0 or 2 indicated. Regions encoding G-domains (amino acid residues 1-166) are indicated with solid color whereas regions encoding the HVR (residues 167-188/189) are striped. Alternative splicing of the *KRAS* transcript is shown to generate KRAS4A or KRAS4B.

Despite these high degrees of homology, the functions of the isoforms do not entirely overlap [7]. Whereas activated forms of each oncoprotein stimulate key pathways such as the mitogen-activated protein kinase (MAPK) pathway, other pathways are differentially activated by RAS oncoproteins, best understood as a consequence of distinct subcellular localizations [8, 9]. Trafficking between cellular membrane compartments and steady-state localizations depend on the various membrane targeting motifs located in the HVRs that either directly afford affinity for the cytosolic face of membranes or direct post-translational modifications that modulate hydrophobicity. These include farnesylation, endoproteolysis and carboxyl methylation of a ubiquitous C-terminal CaaX motif, palmitoylation of adjacent cysteines, polybasic regions (PBRs), and phosphorylation (Fig. 1A) [4, 5, 10].

RAS oncoproteins, also known as classical or canonical RAS proteins, are an evolutionarily considered subfamily of the *RAS* family of small GTPases, which in humans comprise 40 members [11, 12]. The *RAS* family itself is a subset of a much larger group of evolutionarily related small GTPases known as the *RAS* Superfamily, which in addition to the *RAS* family also includes the *RAB, RHO, RAN* and *ARF* families [12-14]. All *RAS* superfamily members, which in humans consist of more than 160 gene products, function as binary molecular switches modulated by the binding of GDP or GTP, which in turn is regulated by exchange factors and GTPase activating proteins [15]. *RAS* superfamily members regulate a plethora of cellular functions including proliferation, differentiation, actin remodeling and vesicular and nuclear pore transport [11]. The broad evolutionary history of the *RAS* superfamily has been the subject of several studies (reviewed in [11-14]). Nevertheless, some aspects of the evolutionary history of the *RAS* subfamily of oncogenes have not been fully addressed, such us the diversification of the *RAS* oncogenes, the generation of the KRAS4A splice variant and the origin of the HVR sequences.

The evolutionary history of KRAS4A has been hampered by its sequence similarity with KRAS4B. Both proteins are generated by the alternative splicing of the fourth coding exons of the *KRAS* gene making their G-domain sequences, normally used in phylogenetic analyses, almost identical (in humans they differ by only four amino acids encoded in the 5’ portion of the 4^th^ exon that precedes the membrane targeting signals of the HVR) (Fig. 1B) [16]. Until recently, KRAS4A has been considered less relevant in tumorigenesis than KRAS4B because of its relatively low expression. However, recent analyses of colorectal tumors have revealed equal expression of the two splice variants [17] and splice variant-specific interactions have been reported [18-20]. Importantly, KRAS4A, but not KRAS4B, has been found to directly regulate hexokinase 1 as a consequence of HVR-driven trafficking to the outer mitochondrial membrane, an interaction that uncovered a unique metabolic vulnerability in tumors expressing relatively high levels of oncogenic KRAS4A, which could have therapeutic potential [16, 18].

These findings and the availability of newly sequenced genomes of basal vertebrates instrumental in tracking *RAS* oncogene diversification, prompted us to study the specific evolution of the RAS oncoproteins, in particular the origins of the HVRs and of the *KRAS* splice variants. We find that *KRAS* is primordial and gave rise to *HRAS* through a gene duplication >600 MYA, that *NRAS* is a duplication of *HRAS* and that KRAS4A arose 475 MYA with the evolution in the common ancestor of jawed vertebrates (cartilaginous fish and bony vertebrates) not by gene duplication but rather by capture into the third intron of the *KRAS* locus of exon 4 of *NRAS*. Despite highly overlapping regulation and function, the persistence of four RAS isoforms with unique membrane-targeting sequences for >400 MY of evolution suggests important and distinct functions.

## RESULTS

### Data mining

**Fig. 2.**
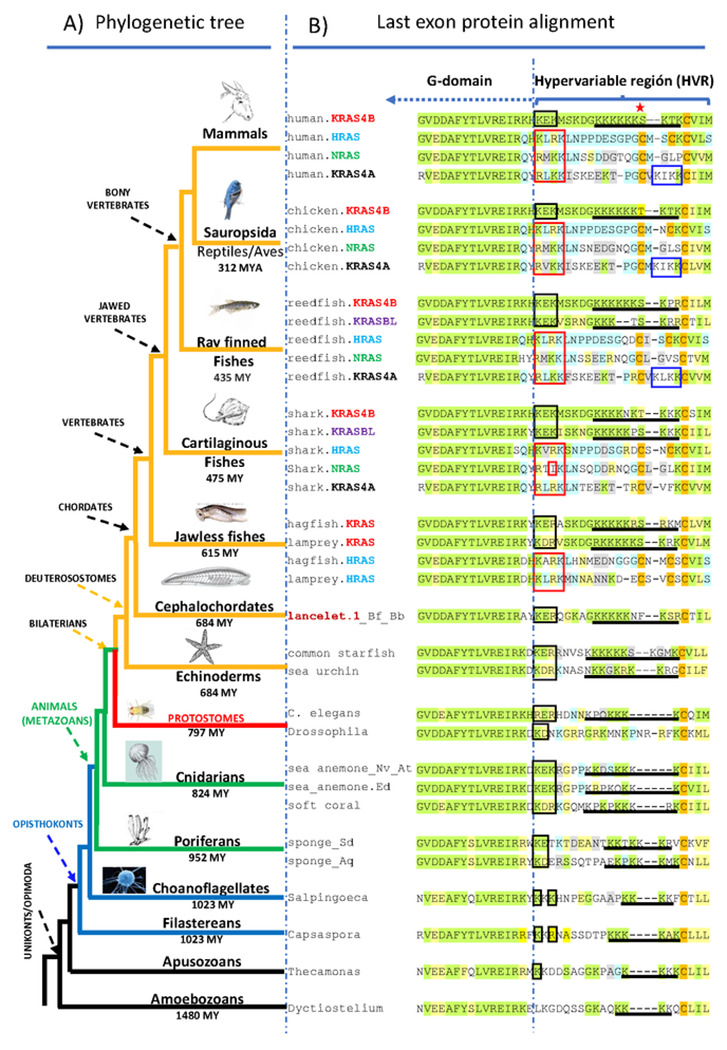
Eukaryotic oncoproteins C-terminal alignment and HVR targeting motifs. A) Simplified phylogenetic tree of eukaryotic organisms. Major groups are indicated with arrows. Distances in millions of years (MY) are indicated below the corresponding branches [64]. Urochordates/tunicates and Plants/Alveolates which lack oncoprotein sequences, and fungi that have extended carboxy-terminal regions are not shown. B) Alignment of protein regions corresponding to exon 4 of human *RAS* oncogenes. Exon 4 consists of the last 15 aa of the G-domain (dashed arrow) and the HVR region (bracket). Membrane targeting motifs are depicted as in the legend in Figure 1A: NB black box, PBD underline, PBR1 red box, PBR2 blue box, and phosphorylation site red star. Green and yellow amino acids are identical or similar to human KRAS4B, respectively; blue and grey identical or similar to human HRAS, respectively.

To study *RAS* oncogene evolution we took advantage of sequenced genomes of lower vertebrate species near evolutionary branching points, such as the jawless fishes lampreys (*P. marinus, E. tridentatus*) and hagfish (*E. burgeri*), which are basal to vertebrates, elephant shark (*C. milii*) basal to cartilaginous fishes, and reedfish (*E. calabaricus*) basal to bony fishes (Fig. 2A) [21-24]. We also compiled representative orthologs of RAS oncoproteins from key major groups of eukaryotes extending over 1000 MY of evolutionary distance, from single celled amoebozoans (*D. discoideum*) to mammals (*H. sapiens*). We did not find orthologous RAS oncoprotein sequences in the closest relatives of vertebrates, urochordates/tunicates (i.e., *C. intestinalis*) and in bikonts/diphoda (i.e., plants and alveolates) [14]. For sequences, organisms, and identification numbers see Suppl. File 1.

### A novel *RAS* oncogene-related sequence in vertebrates

**Fig. 3.**
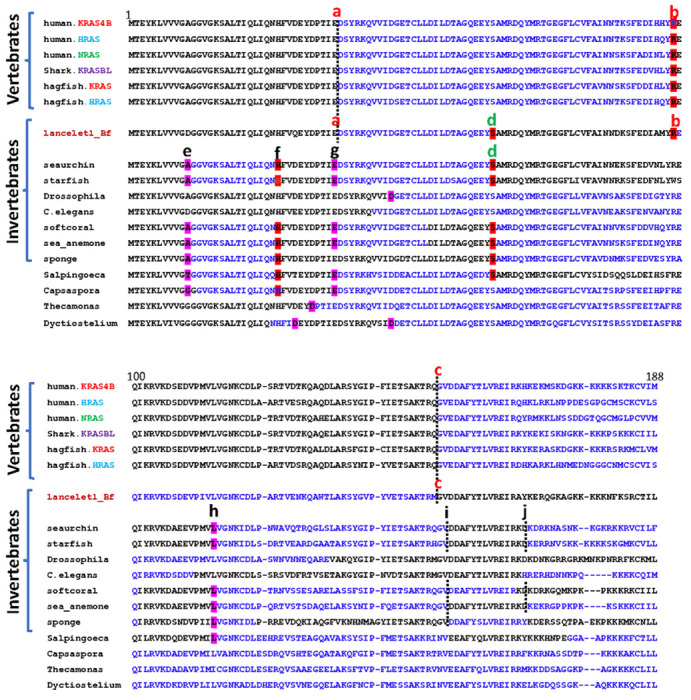
Conservation of intron/exon gene structures. The alignment of representative full-length sequences from humans to single-celled eukaryotes was generated with the MAFFT server with default parameters. Exons are depicted in alternate black and blue letters. Location of introns are indicated as red a-b letters (vertebrate and cephalochordate), green d letters (cephalochordate, other invertebrate and single celled organisms), and black e-j letters (invertebrate, except cephalochordates, and single celled organisms). Introns in phase 0, between amino acids, are additionally indicated by black dotted vertical lines. The amino acids split by introns in phases 1 and 2 are shaded in cyan and red, respectively.

Sequences related to each one of the human oncoproteins HRAS, NRAS, and splicing variants KRAS4B and KRAS4A were found in all vertebrates except most basal jawless fishes (lampreys and hagfish) that only have HRAS and KRAS4B (Fig. 2; Suppl. File 1). In addition, all vertebrates except mammals, birds, and jawless fishes, have an additional, previously unrecognized RAS GTPase we have designated KRAS4B-like (KRASBL) because its high similarity with KRAS4B in sequence and in membrane targeting motifs, although its CaaX motif is unique among the vertebrate RAS oncogenes in signaling for geranylgeranylation (see below) (Fig. 2B; Suppl. File 1). A common evolutionary origin for all vertebrate RAS oncogenes is suggested by the conservation in all of them of four coding exons separated by three homologous introns that we designate a, b and c in phases 0, 2, and 0 respectively, (Fig. 1B and Fig. 3)

### *RAS* oncogene-related sequences in invertebrates, fungi, and single celled eukaryotes

In contrast to vertebrates, we mostly found only one oncoprotein-related sequence in invertebrates, fungi and single celled eukaryotic organisms; exceptions were the amphioxus or lancelets (*Branchiostoma*) genes we have designated lancelet1 and lancelet2 in cephalochordates and the yeast *S. cerevisiae* genes RAS1 and RAS2 in fungi (Suppl. File 1). However, RAS1 and RAS2 are the product of a well-known unique duplication in *S. cerevisiae* [25], and lancelet2 also appears to be a cephalochordate-specific event unrelated to the core evolution of *RAS* oncoproteins since *lancelet2* has a different gene structure and lacks HVR oncoprotein characteristics (see below) (Suppl. Fig. 1). The evolutionary relationship between vertebrate, invertebrate, and single celled eukaryote oncogenes is also supported by: (i) the exon-intron structure of the cephalochordate gene *lancelet1* which consists of introns a, b and c like vertebrates, but with an additional intron that splits exon 2 (intron d) also present in invertebrate oncogenes and in single celled *Salpingoeca*, and (ii) the presence of additional introns (e-j) in invertebrates (except cephalochordates and protostomes) and in single celled eukaryotes (*Salpingoeca* and *Capsaspora*) (Fig. 3). On the other hand, fungi are intronless except *Aspergillus*, which has unique combinations of intron positions like the most ancient eukaryotes *Thecamonas* and *Dyctiostelium* (Fig. 3 and Suppl. File 1).

### RAS oncoprotein G-domain sequence characteristics

Eukaryotic RAS oncoproteins are 184 to 189 amino acids long due to variations in HVR size (18-23 amino acids), except fungi sequences which have 213-322 amino acids because of extended N- and C-termini (Fig. 2B, Suppl. File. 1) [26]. Oncoprotein G-domain sequences (residues 1-166) are highly similar (Suppl. Fig 2). In comparison with the human proteins, amino acid pairwise identities range from 93-100% in vertebrates, 80-91% in non-vertebrate animals, 72-80% in single celled eukaryotes, and 63-68% in fungi (Suppl. Fig 3). In contrast, MRAS, RRAS and RRAS2/TC21, which are considered the RAS family members most closely related to the four RAS oncoprotein subfamily, are only 56-62% identical (Suppl. Fig. 3) [11]. Moreover, in phylogenetic analyses, all eukaryotic oncoprotein G-domain sequences of DNA and protein cluster together, except fungi in which the oncoproteins cluster with MRAS, RRAS and RRAS2/TC21 protein sequences (Suppl. Fig. 4).

**Fig. 4.**
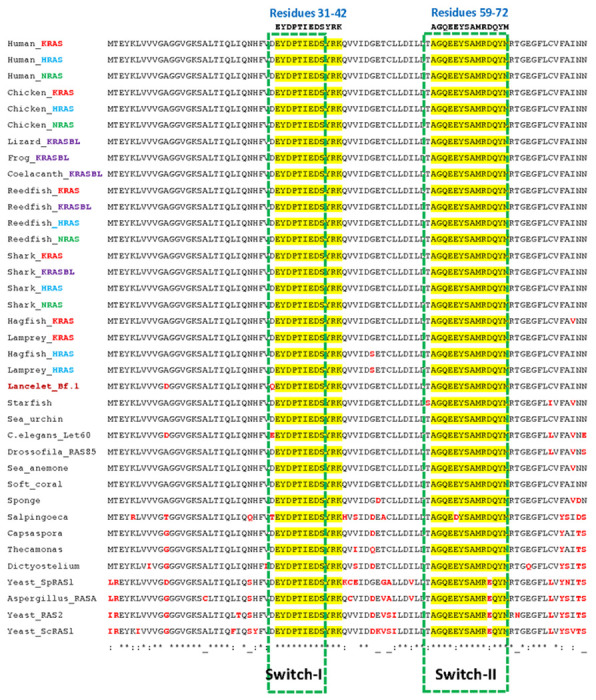
The switch-I and -II regions of eukaryotic oncoproteins are strictly conserved over 1480 MY. Clustalw alignment of eukaryotic oncoprotein G-domain effector lobe (amino acids 1-86) sequences. Highlighted in yellow and indicated above the alignment 100% conserved residues 31-42 EYDPTIEDSYRK, and 59-72 AGQEEYSAMRDQYM. Green boxes, switch-regions I and II. Amino acids different from human oncoproteins are indicated in red. The alignment was generated using the MAFFT server with default parameters. Amino acid identity: identical (*); strongly similar (:); weakly similar (.).

Interestingly, residues 31-42 EYDPTIEDSYRK, which overlap with the switch-I functional element (residues 30-40), appear oncoprotein-specific since they are 100% identical in all representative sequences gathered for this study, as well as in all additional sequences we could retrieve by BLAST searching NCBI protein databases (Fig. 4 and Suppl. Fig 5). Likewise, residues 59-72 AGQEEYSAMRDQYM, which overlap with Switch-II (residues 58-72), are identical in all animal oncoproteins and only differ by one residue in fungi (AGQEEYSAMR**e**QYM) and in single celled choanoflagellates *S. rosseta* (AGQE**d**YSAMRDQYM) (Fig. 4 and Suppl. Fig. 5 ). In comparison, each of these regions differ by 2-3 residues in MRAS **d**YDPTIEDSY**l**K/AGQEE**f**SAMR**e**QYM and in RRAS and RRAS2/TC21 **d**YDPTIEDSY**t**K/AGQEE**fg**AMR**e**QYM (Suppl. File. 1). Thus, these two sequences can be considered a signature of the evolutionary path to the vertebrate oncoproteins.

### Evolutionary conservation of membrane targeting motifs within isoforms

The primary association of RAS isoforms with cellular membranes as well as trafficking between membrane compartments and steady-state subcellular localization is specified by post-translational modifications of cysteine and serine residues and by the presence of polybasic regions in the HVR (Fig. 1A) [4, 5]. In this region all isoforms possess a C-terminal CaaX motif, also known as a CaaX box (cysteine, aliphatic x2, variable), that is necessary but not sufficient for delivery of the proteins to the plasma membrane (PM). For PM delivery each isoform requires a so-called second signal immediately upstream of the CaaX sequence. For KRAS4B the second signal is a polylysine sequence that affords a strong positive charge (+8) at physiological pH and thereby allows for an electrostatic interaction with the negatively charged inner leaflet of the PM [10, 27]. The three other isoforms possess one (NRAS and KRAS4A) or two (HRAS) cysteines upstream of the CaaX sequence that are modified by palmitate, which provides the second signal. Because palmitoylation is readily reversed, this type of second signal allows for regulation of membrane targeting. The three palmitoylated isoforms also possess a short polybasic sequence (PBR1, +3 charge) upstream of the modified cysteines (Fig. 1A). Interestingly, KRAS4B has two lysines in this region, that we designate a neutralized basic (NB) sequence because their charge is negated by an intervening glutamic acid. KRAS4B possesses a conserved serine within its polybasic region. This serine is a site for phosphorylation [28-32], a posttranslational modification that partially neutralizes the positive charge and weakens PM targeting [28, 29]. KRAS4A has a second short polybasic sequence (PBR2, +3 charge) between the palmitate and the farnesyl modifications that has been shown to support PM targeting in the absence of palmitoylation (Fig. 1A) [17].

To analyze the evolutionary conservation of these targeting motifs in eukaryotes, we aligned the 18-23 amino acid oncoprotein HVR region from humans to amoebas (Fig. 2). As expected, all sequences (Fig. 2; Suppl. File. 1) including those of fungi (Suppl. Fig. 6) have a terminal CaaX motif. The CaaX motif is shared by other members of the RAS superfamily and is also found in more than two hundred other proteins [33]. The CaaX motif directs irreversible modification of the cysteine with a 15-carbon farnesyl polyisoprene lipid, except when the terminal amino acid is L or F, which direct modification by a distinct prenyltransferase with the 20-carbon geranylgeranyl polyisoprene lipid [4, 5]. Our evolutionarily representative data and all the sequences we could retrieve by searching NCBI protein databases reveal that all vertebrate isoforms have a CaaX motif that directs farnesylation except for HRAS from cartilaginous fish and KRASBL sequences that have the geranylgeranylation L or F residues (Fig. 2; Suppl. Fig. 6). Interestingly most non-vertebrate species also have an L/F terminal residue. Exceptions were most fungi and some nematodes such as the ones from the *Caenorhabditis* genus (Fig. 2, Suppl. Fig. 6). Importantly, since the C-20 geranylgeranyl modification affords higher affinity for phospholipid bilayers than does the C-15 farnesyl modification [34], this observation suggests evolutionary pressure to weaken membrane affinity in vertebrates allowing for greater regulation of membrane association. This observation also suggests an enhanced role for isoprenylcysteine carboxyl methylation, the final step of CaaX processing, because it is readily reversible [4, 5] and augments the membrane affinity of farnesylated proteins to a much greater degree than geranylgeranylated proteins [34].

In these analyses, the four amino acid polybasic PBR1 motif showed a consensus motif of three positively charged K or R amino acids and one hydrophobic amino acid located in the second positions (K/R-hydrophobic-K/R-K/R) (Fig. 2). A single PBR1 motif was found in all HRAS and NRAS isoforms except in NRAS from cartilaginous fishes that have a hydrophobic residue in the third position (Fig. 2). This suggests that a hydrophobic residue operates in conjunction with basic residues in affording affinity for the phospholipid bilayer, perhaps allowing electrostatic interaction with anionic head groups while the intervening side chain accesses the deeper lipid domain. PBR1 plus PBR2 motifs were found in all KRAS4A isoforms except in KRAS4A from cartilaginous fishes that lack PBR2 (Fig. 2). Analysis of the three amino acid NB motif yielded a consensus sequence of two positively charged polar K or R amino acids flanking one negatively charged polar amino acid (K/R-E/D-K/R) and was found in all KRS4B, KRASBL and in non-vertebrate animal sequences except in arthropods and in porifera (sponges) (Fig. 2). Finally, a polybasic domain (PBD) immediately upstream of the CaaX motif formed by at least four positively charged residues was found in all KRS4B, KRASBL and in all non-vertebrate sequences including single celled eukaryotes (Fig. 2; Suppl. File 1) other than fungi. This suggests that the PBD was the primordial evolutionary solution to augmenting the affinity for membranes of the prenylcysteine and predates palmitoylation by 800 MY. PBDs in mammals have a phosphorylation acceptor residue (T/S) located tree amino acids upstream of the CaaX motif, which is conserved in the same position in all vertebrate KRS4B and KRASBL sequences (Fig. 2), suggesting that modulation of the charge by phosphorylation is conserved and therefore functionally important.

### Expansion of RAS oncoproteins in vertebrates

The existence of a single RAS oncogene homolog in invertebrates, two (*hras* and *kras*) in jawless fishes the most basal extant vertebrates, and four (*kras, hras, nras*, and *krasbl*) in jawed vertebrates (Fig. 2), suggests that two rounds of gene duplication are behind the oncoprotein expansion in vertebrates, which coincide with the proposed two rounds of whole-genome duplication (WGD) that shape early vertebrate evolution [35-37].

### Generation of KRAS4B and HRAS

**Fig. 5.**
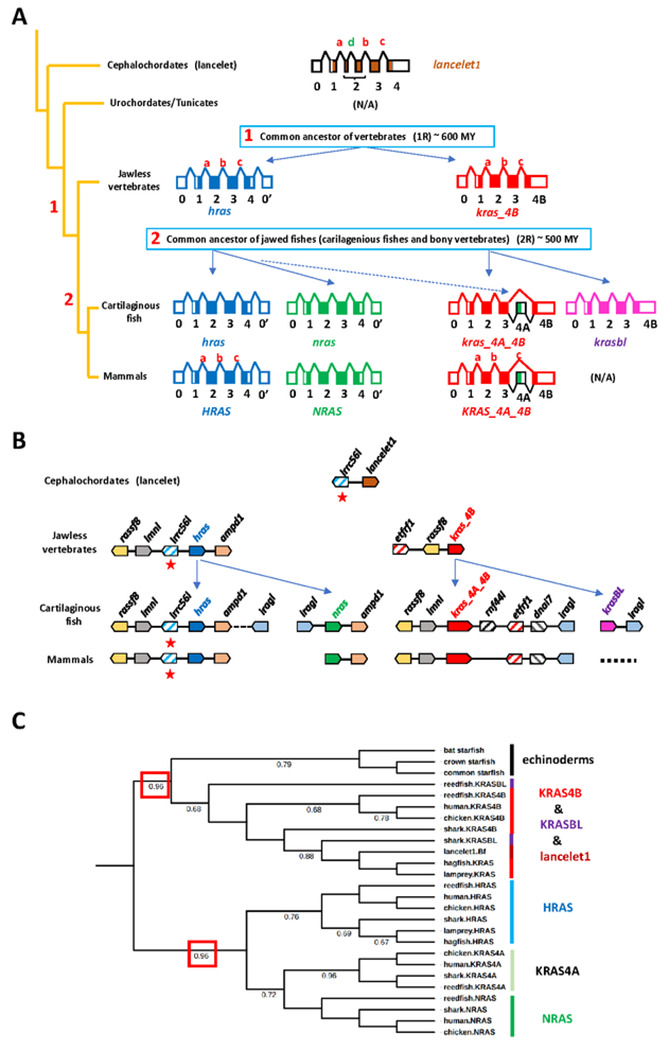
Vertebrate RAS oncoproteins expansion. (A) Scheme of oncoprotein expansion from cephalochordates to mammals. Oncogene exon-intron structures are depicted with exons as boxes (1-4 coding, 0 and 0’ non-coding) and introns as lines connecting the exons (a-d). (B) Oncoprotein genomic organization. Genes are depicted by colored polygons pointing in the direction of transcription. Evolutionarily conserved solitary genes (striped) and duplicated genes (solid) are indicated. Gene names are indicated above the polygons and **lrrc56** is also indicated by a red star. (C) Phylogenetic analysis separates invertebrate and KRAS4B related sequences from those of palmotoylated isoforms. The phylogenetic tree reveals two clusters, one with KRAS4B, KRASBL and invertebrate sequences and a second one with NRAS, KRAS4A and HRAS (red boxed bootstrap values). Phylogenetic analysis was generated with DNA sequences corresponding to mammalian last exon using the tools on the Phylogeny.fr web site (MUSCLE for alignment, Gblocks for curation, and PhyML for tree building) [57, 63, 65]. Bootstrap values higher than 65% are indicated.

The most parsimonious scenario for the first round of duplications could be one in which the deuterostome oncogene in the common ancestor of chordates (cephalochordates, tunicates and vertebrates) evolved by speciation to form *lancelet1* in cephalochordates, was lost in tunicates, and duplicated to generate *hras* and *kras* in the common ancestor of vertebrates (Fig. 5A). During this process invertebrate intron d was lost, *hras* gained an additional non-coding exon (0’), and *kras* and *hras* evolve distinct HVR vertebrate characteristics: the location of a phosphorylation acceptor residue (T/S) tree amino acids upstream of the CaaX motif in KRAS and, the formation of PBR1 instead of the NB motif, and the acquisition of two additional cysteines instead of the PBD motif in HRAS (Fig. 2 and Fig. 5A). The fact that invertebrate HVR characteristic (NB, PBD and only one cysteine) were maintained in *kras* and modified in *hras* suggests that invertebrate oncogenes and vertebrate *kras* genes are orthologs (evolved by vertical descent) while *hras* genes are paralogous (evolved by duplication) (Fig. 2 and Fig. 5A). Moreover, phylogenetic analyses place in the same branch invertebrate and *kras* sequences (Fig. 5C). Interestingly, synteny (the maintenance or co-localization of groups of genes in the chromosomes of different species) which indicates evolution by vertical descent was not observed between *kras* and *hras* because the gene *lrrc56* that is just downstream of *hras* and *lancelet1* is missing in *kras* genomic area (Fig. 5B). However it is reasonable to infer that *lrrc56* disappeared from the *kras* locus after the first round of duplication because *lrrc56* is a single-copy gene as we have not found duplications of this gene in neither the Ensemble paralog database or by BLAST searches in the NCBI database (Fig. 5B and Suppl. Fig. 7), suggesting selective pressure against duplication. Along the same lines, after the duplication of *hras* that generated *nras* (see below), *lrrc56* disappeared from the *nras* genomic area while *ampd1* was maintained after the duplication (Fig. 5B and Suppl. Fig. 7) further suggesting negative selection for duplicate *lrrc56*.

### Generation of KRASBL AND NRAS

In a second round of duplications in the common ancestor of jawed vertebrates (cartilaginous fish and bony vertebrates), *hras* generated *nras*, and *kras* generated *krasBL* (Fig. 5A). In agreement with this proposed duplication: (i) the gene structure of *kras* in jawless fish and *krasbl* are identical, as well as that of *hras* and *nras* (Fig. 5A); (ii) phylogenetic analyses group together *kras* with *krasbl*, and *hras* with *nras* sequences (Fig. 5C); and (iii) there is presence of synteny, with the *ampd1* gene located just upstream of both *hras* and *nras* genes (Fig. 5B and Suppl. Fig. 7).

Interestingly, it has been proposed that while the first WGD in the ancestor of vertebrates took place by direct genome duplication within a species (autotetraploidization), the second round of WGD took place by a genome duplication following interspecific hybridization (allotetraploidization) from two now extinct progenitor species that fuse their genomes in the common ancestor of jawed vertebrates [35]. In this case it could be possible that *nras* as well as *krasbl* evolved by speciation in either one of the two extinct progenitors and were brought together with *hras* and *kras* in the allotetraploidization genome duplication that generated the jawed vertebrate ancestor. The fact that *krasbl* was later lost upon the emergence of mammals and birds suggests redundant function in contrast to *hras* and *nras* which are retained suggesting altered funcitons (Fig. 2).

### Generation of spliced isoform KRAS4A.

The difference between KRAS4B and KRAS4A resides in the alternative use of the two last exons 4A and 4B of *KRAS* by mutually exclusive alternative splicing, a tightly regulated process (Fig. 1B and Fig. 5A) [38]. Like *nras* and *krasbl*, exon 4A was formed in the common ancestor of jawed vertebrates since it can be found between *kras* exons 3 and 4B in cartilaginous fishes, but it is absent in the corresponding genomic regions of more ancient jawless fishes (Fig. 5A and Suppl. Fig 8). Phylogenetic analyses indicate that exon 4 of *nras* and exon 4A have highest DNA similarity, which suggests that a duplication of *nras* exon 4 could have generated *kras* exon 4A (Fig. 5C). Additionally, there is conservation of synteny between *hras, nras*, and *kras* genomic regions indicating that some extent of genomic recombination took place during the second round of duplications that could have favored the duplication and insertion of *nras* exon 4 into the *kras* locus by exon shuffling (Fig. 5B and Suppl. Fig 7) [39, 40]. Once brought together, exons 4A and 4B followed a frequent evolutionarily outcome for tandemly duplicated exons consisting of mutually exclusive alternative splicing [38, 41-43]. Interestingly, spliced exon 4A has the characteristics frequently found in alternatively spliced isoforms in that one isoform dominates at the protein level, the generation from homologous exons did not disrupt the functional G-domain, and the splice forms are evolutionarily conserved for over 450 MY [44].

To determine how unique is alternative splicing of 3’ exons in the RAS superfamily we analyzed all 167 members [14]. We found nine additional examples of carboxyl terminal alternative splicing: CDC42, HRAS, Arl6, TRIM23/Ard1, Rab8A/Mel, Rab28, Rab35, Arl8B, and Rab15. However, only the *CDC42* behaves like the *KRAS* locus in generating splice variants with distinct membrane-targeting HVRs (Suppl. Fig. 9) [45, 46]. Alternative splicing of *HRAS* has been previously described and yields a non-functional protein lacking a membrane-targeting motif [47].

## DISCUSSION

Although evolution of the RAS superfamily of small GTPases has been the subject of several analyses, the focus has been on the G domains shedding little light on the origins of the multiple RAS oncogene products themselves. Ras oncoproteins are composed of two distinct domains, a guanine-nucleotide binding domain (G domain) with a highly conserved structure and an unstructured HVR. The expanded availability of sequenced genomes of basal vertebrates has allowed us to analyze the evolution of the HVRs and obtain insight into the alternative splicing of the *KRAS* locus. We report several new observations including a novel KRAS4B paralog we designated KRASBL that appeared with the emergence of vertebrates but was subsequently lost in mammals and birds. The transience of KRASBL contrasts with the strict conservation of the other four RAS oncoproteins and argues against functional redundancy of the retained four. We show that the switch 1 and 2 regions of the G-domain are identical in RAS oncogene orthologs throughout evolution, providing a signature for this subset of RAS superfamily genes and suggesting intense evolutionary pressure to maintain signaling down the MAPK and perhaps other pathways. We found that the PBR1 motif found in HRAS, NRAS and KRAS4A is highly conserved and incorporates a hydrophobic residue interspersed among three basic residues. This suggests a membrane insertion motif that has not been explored biophysically. We also found that the NB motif consisting of two basic residues flanking an acidic residue is conserved in all KRAS4B orthologs including those found in invertebrates. The function of the NB motif in membrane targeting has not been investigated. We found that all vertebrate KRAS4B and KRASBL sequences include a phosphate acceptor (S or T) three residues upstream of the CaaX motif, suggesting that modulation of the negative charge by phosphorylation is functionally important. Importantly, we found that, with the exception of HRAS in cartilaginous fish and KRASBL, all RAS oncogene orthologs have a CaaX motif that signals for farnesylation rather than geranylgeranylation. In contrast, the majority of RAS superfamily small GTPases as well as orthologs of RAS oncoproteins prior to jawless fishes have CaaX motifs that signal for geranylgeranylation. Thus, it appears that diminishing the hydrophobicity of the prenyl lipid that modifies RAS oncogenes was essential for their function. This may explain why KRAS4BL was lost from mammals and birds. This suggests that the modulation of the membrane affinity of farnesylated RAS oncoproteins by palmitoylation/depalmitoylation or phosphorylation/dephosphorylation was an important adaptation for vertebrate oncoproteins.

Our analysis suggests that *KRAS* is primordial and gave rise to *HRAS* by gene duplication 615 MYA, which in turn generated *NRAS* by another duplication event. In contrast, KRAS4A evolved not by gene duplication but rather by incorporation of exon 4 of *NRAS* into the 3rd intron of the *KRAS* locus 475 MYA. RAS oncogenes have been the focus of intense investigation by cancer biologists for more than 40 years because of the prevalence of oncogenic RAS mutations in human cancer. Through most of those decades RAS proteins were thought to be functionally interchangeable. The persistence of the four RAS isoforms through >400 MY of evolution argues strongly against this view and suggests unique functions driven by the HVRs, including the HVRs of the splice variants of the *KRAS* locus. This revised view is consistent with recent analysis of tumors driven by oncogenic RAS mutations where differences are observed not only among the isoforms but also among different mutations of the same isoform [48, 49]. Targeted therapies for RAS-driven cancers must take into account these differences and the evolutionary analysis presented here will contribute to a more complete understanding of the relevant parameters.

## METHODS

### Data mining and sequence analysis

This paper analyzes existing, publicly available data. We collected the protein and DNA sequences used in this study from online genomic, proteomic and nucleotide NCBI databases (https://www.ncbi.nlm.nih.gov). Hagfish and pacific lamprey sequences were retrieved from Ensembl (http://www.ensembl.org/) and SIMRBASE (https://genomes.stowers.org) databases, respectively. Whole sequences, organisms’ scientific names, identification numbers, and individual sequence details are provided in Supplemental File 1. All sequences used in this study were checked for errors and curated manually.

Data mining was performed as previously described by [50, 51]. We searched the NCBI protein database with the BLASTP program using as a bait known human oncoproteins [52]. For each collected protein of interest, the corresponding cDNA and gene information were retrieved using the Nucleotide and Gene links in the database related information window. Hagfish sequences were obtained from Ensembl orthologs database and from searches in the hagfish genome with the TBLASN program with multiple starting queries using Ensembl servers. Pacific lamprey sequences were retrieved by searching with related sea lamprey cDNAs the pacific lamprey genome at SIMRBASE.

Alignments of protein and DNA sequences were performed using the MAFFT server (https://mafft.cbrc.jp/alignment/server/) [53]. The SMART server (http://smart.embl-heidelberg.de/) [54] was used to determine the G-domain (RAS domain) in the protein sequences. Exons and intron positions and phases (phase 0 introns are located between codons, phase 1 introns between the first and second nucleotide of a codon, and phase 2 introns between the second and third nucleotide) were retrieved from the sequence’s NCBI or Ensembl gene information or determined as described [55] with the “align two sequences” option of the NCBI BLAST program and manual supervision of the splice consensus signals. An intron was considered homologous when it occupies the same amino acid position in the alignments of the protein sequences and has the same intron phase. Pairwise identities for G domain protein sequences were determined by aligning each sequence with the human oncoprotein G-domains using the “align two sequences” option of the NCBI PLASTP program [52]. Alternative carboxyl terminal splicing variants in the Ras superfamily were obtained from the UNIPROT server (http://www.uniprot.org/help/uniprotkb) [56].

### Phylogenetic analyses

All amino acid and nucleotide sequences used in the phylogenetic analyses are provided in Supplemental File 1. Sequences were aligned using multiple sequence comparison by log-expectation (MUSCLE) with default setting [57] and ambiguous regions curated with Gblocks [58]. The protein phylogenetic tree was reconstructed using the neighbor-joining method implemented in the BioNJ program [59] with 1000 bootstrap replicates and the Jones-Taylor-Thornton (JTT) substitution model [60]. The DNA phylogenetic trees were reconstructed using the maximum likelihood method implemented in the PhyML program (v3.1/3.0 aLRT) [61]. The HKY85 substitution model was selected assuming an estimated proportion of invariant sites (of 0.270) and 4 gamma-distributed rate categories to account for rate heterogeneity across sites. The gamma shape parameter was estimated directly from the data (gamma=1.394). Reliability for an internal branch was assessed using the aLRT test (SH-Like). The resulting phylogenies were visualized using the Interactive Tree of Life (iTOL) platform (https://itol.embl.de/) [62]. The programs MUSCLE, Gblocks, BioNJ and PhyML are available on the phylogeny.fr platform (https://www.phylogeny.fr/) [63].

## Supplementary Material

Suppl. File 1 **Suppl. File 1**. List of all sequences, accession numbers, and species scientific names used in this study. Exons are represented with alternate black and blue colors. Amino acids in red letters means they are split between two exons. G-domains are underlined.

Suppl. Fig 1 **Suppl. Fig. 1. Lancelet2 has distinct gene structure and HVR characteristics.** Clustalw MAFFT alignment of lancelet1 and lancelet2 cephalochordate sequences. Exons are depicted in alternate black and blue letters. Whereas lancelet1 has homolog introns a-c like vertebrate oncogenes and intron d like other invertebrates, lancelet2 has a single intron (indicated by a red arrow). The dicysteine motif that includes the CaaX cysteine of Lancelet2 (highlighted in orange) is also different from other oncogenes. Amino acid identity: identical (*); strongly similar (:); weakly similar (.).

Suppl. Fig 2 **Suppl. Fig. 2. Eukaryotic G-domain sequences are highly similar.** The alignment of the full-length G-domain oncoprotein sequences from amoebas to humans was generated by the MAFFT server with default parameters. Upper panel effector lobe (residues 1-86) and lower panel allosteric lobe (residues 87-166). Switch-regions I and II (green boxes) and GTP/GDP binding elements (dashed pink boxes). Arrows and rectangles above the sequences α-helices and β-sheets, respectively. Amino acid identity: identical (*); strongly similar (:); weakly similar (.).

Suppl. Fig 3 **Suppl. Fig. 3. Eukaryotic G-domains pairwise amino acids identity**. Heat map representing amino acids Blastp pairwise identities in comparison with human oncoproteins. Numerical values are depicted inside the cells, which are colored from green (low similarity) to red (high similarity).

Suppl. Fig 4 **Suppl. Fig. 4. Evolutionary relationships between eukaryotic G-domain sequences.** (A) DNA sequences. (B) Protein sequences. Evolutionary relationships between DNA and protein sequences corresponding exclusively to G-domains, where analyzed using the tools on the Phylogeny.fr web site [63], MUSCLE for alignment, Gblocks for curation, PhyML for DNA tree building, and BioNJ with 1000 bootstrap replicates and the JTT substitution model for protein sequences [57, 63, 65]. The resulting outputs in the Newick files format were visualized using the Interactive Tree of Life platform ^52^. Bootstrap values are indicated below tree branches with value at group branch points boxed in red. G-domain sequences from RRAS, MRAS and TC21 which are the closest members to classical oncoproteins in the RAS family, where used as outgroup [12].

Suppl. Fig 5 **Suppl. Fig. 5. Oncoprotein specific signature conservation in eukaryotes.** Protein Blast searches in NCBI protein eukaryotic databases were performed in Blastp with default parameters using as a bait the oncoprotein specific signature (EYDPTIEDSYRKxxxxxxxxxxxxxxxxAGQEEYSAMRDQYM) corresponding to the effector domain. Single non identical amino acids in one choanoflagellate species (*S. rosseta*) and in all Fungi are indicated with black rectangles.

Suppl. Fig 6 **Suppl. Fig. 6. The CaaX motifs of oncoproteins.** Whereas the sequences predict geranylgeranylation signal (L or F terminal residues) in KRASBL, HRAS from cartilaginous fishes, and in most non-vertebrate groups they predict farnesylation for all vertebrate oncoproteins since jawed fish other than KRASBL that is not found in birds and mammals. CaaX terminal residues from BLASTP searches are shown as multiple alignment outputs.

Suppl. Fig 7 **Suppl. Fig. 7. Conservation of oncoproteins genomic architecture (synteny) from humans to lancelets**. The figure depicts *Ras* oncogene genomic regions from cephalochordates to mammals. Genes are depicted as arrows pointing to the direction of transcription. Evolutionarily conserved solitary genes (hashed), duplicated genes (solid), and non-conserved (outline) are shown. Genomic coordinates are indicated below each species name. GenBank assembly accession numbers are human, GCF_000001405.39; lizard, GCF_009819535.1; frog, GCF_000004195.4; coelacanth, GCF_000225785.1; reedfish, GCF_900747795.1; skate, GCF_010909765.1; elephant shark, GCF_018977255.1; hagfish, GCA_900186335.2; lamprey, GCF_010993605.1; and lancelet, GCF_000003815.2.

Suppl. Fig 8 **Suppl. Fig. 8. Appearance of *KRAS* Exon 4A in vertebrates.** Genomic regions of shark, hagfish and lamprey showing the locations of exons 4B (blue letters) and 4A (green letters). Stop codons are highlighted in red. Genomic regions: shark NW_006890288.1 (547536..553042); hagfish, Eburgeri 3.2: FYBX02009586.1: 2433624:2434677:1; lamprey Chr. 65: NC_046133.1, 5454894 to 5455895. GenBank assembly accession numbers: shark, GCF_000165045.1; hagfish GCA_900186335.2; lamprey, GCF_010993605.1.

Suppl. Fig 9 **Suppl. Fig. 9. Alternative carboxyl terminal splicing variants in the Ras superfamily.** NCBI sequences with exons in alternate colors, and screen captures of the genomic gene structures with the spliced exons indicated by arrows. Time of appearance of isoforms was deduced from NCBI protein BLAST searches. Isoforms as described in the Uniprot server that are produced by alternative splicing affecting the carboxyl terminal region are shown in the box.
