# Supplementary material for: Origin and Evolution of RAS Oncoprotein Membrane Targeting
Source: Res Sq. 2023 Jan 20:rs.3.rs-2485219. Preprint. [Version 1] doi: 10.21203/rs.3.rs-2485219/v1 (PMC9882654; doi:10.21203/rs.3.rs-2485219/v1)
Supplement: Suppl. File 1 — List of all sequences, accession numbers, and species scientific names used in this study. Exons are represented with alternate black and blue colors. Amino acids in red letters means they are split between two exons. G-domains are underlined. [file Suppl.File.1.pdf]

1. DEUTEROSTOMES

1.1 VERTEBRATES

1.1.2 MAMMALS

Human.KRAS4B NP_001356716.1 GTPase KRas isoform b [Homo sapiens]
MTEYKLVVVGAGGVGKSALTIQLIQNHFVDEYDPTIEDSYRKQVVIDGETCLLDILDTAGQEEYSAMRDQYMRTGEGF
LCVFAINNTKSFEDIHHYREQIKRVKDSEDVPMVLVGNKCDLPSRTVDTKQAQDLARSYGIPFIETSAKTRQGVDDAF
YTLVREIRKHKEKMSKDGKKKKKKSKTKCVIM

Human.HRAS NP_001123914.1 GTPase HRas isoform 1 [Homo sapiens]
MTEYKLVVVGAGGVGKSALTIQLIQNHFVDEYDPTIEDSYRKQVVIDGETCLLDILDTAGQEEYSAMRDQYMRTGEGF
LCVFAINNTKSFEDIHQYREQIKRVKDSDDVPMVLVGNKCDLAARTVESRQAQDLARSYGIPYIETSAKTRQGVEDAF
YTLVREIRQHKLRKLNPPDESGPGCMSCKCVLS

Human.NRAS NP_002515.1 GTPase NRas [Homo sapiens]
MTEYKLVVVGAGGVGKSALTIQLIQNHFVDEYDPTIEDSYRKQVVIDGETCLLDILDTAGQEEYSAMRDQYMRTGEGF
LCVFAINNSKSFADINLYREQIKRVKDSDDVPMVLVGNKCDLPTRTVDTKQAHELAKSYGIPFIETSAKTRQGVEDAF
YTLVREIRQYRMKKLNSSDDGTQGCMGLPCVVM

Human.KRAS4A NP_001356715.1 GTPase KRas isoform a [Homo sapiens]
MTEYKLVVVGAGGVGKSALTIQLIQNHFVDEYDPTIEDSYRKQVVIDGETCLLDILDTAGQEEYSAMRDQYMRTGEGF
LCVFAINNTKSFEDIHHYREQIKRVKDSEDVPMVLVGNKCDLPSRTVDTKQAQDLARSYGIPFIETSAKTRQRVEDAF
YTLVREIRQYRLKKISKEEKTPGCVKIKKCIIM

Human.RRAS NP_006261.1 ras-related protein R-Ras [Homo sapiens]
MSSGAASGTGRGRPRGGGPGPGDPPPSETHKLVVVGGGGVGKSALTIQFIQSYFVSDYDPTIEDSYTKIC
SVDGIPARLDILDTAGQEEFGAMREQYMRAGHGFLLVFAINDRQSFNEVGKLFTQILRVKDRDDFPVVLV
GNKADLESQRQVPRSEASAFGASHHVAYFEASAKLRLNVDEAFEQLVRAVRKYQEQELPPSPPSAPRKKG
GGCPCVLL

Human.MRAS NP_001078518.1 ras-related protein M-Ras isoform 1 precursor [Homo sapiens]
MATSAVPSDNLPTYKLVVVGDGGVGKSALTIQFFQKIFVPDYDPTIEDSYLKHTEIDNQWAILDVLDTAGQEEFSAMR
EQYMRTGDGFLIVYSVTDKASFEHVDRFHQLILRVKDRESFPMILVANKVDLMHLRKITREQGKEMATKHNIPYIETS
AKDPPLNVDKAFHDLVRVIRQQIPEKSQKKKKKTKWRGDRATGTHKLQCVIL

Human.TC21/RRAS2 NP_036382.2 ras-related protein R-Ras2 isoform a [Homo sapiens]
MAAAGWRDGSGQEKYRLVVVGGGGVGKSALTIQFIQSYFVTDYDPTIEDSYTKQCVIDDRAARLDILDTAGQEEFGAM
REQYMRTGEGFLLVFSVTDRGSFEEIYKFQRQILRVKDRDEFPMILIGNKADLDHQRQVTQEEGQQLARQLKVTYMEA
SAKIRMNVDQAFHELVRVIRKFQEQECPPSPEPTRKEKDKKGCHCVIF

1.1.3 SAUROPSIDAE (REPTILES/AVES)

Chicken.KRAS4B NP_001243091.1 GTPase KRas [Gallus gallus]
MTEYKLVVVGAGGVGKSALTIQLIQNHFVDEYDPTIEDSYRKQVVIDGETCLLDILDTAGQEEYSAMRDQYMRTGEGF
LCVFAINNTKSFEDIHHYREQIKRVKDSEDVPMVLVGNKCDLPSRTVDTKQAQDLARSYGIPFIETSAKTRQGVDDAF
YTLVREIRKHKEKMSKDGKKKKKKTKTKCIIM

Chicken.HRAS NP_990623.1 GTPase HRas [Gallus gallus]
MTEYKLVVVGAGGVGKSALTIQLIQNHFVDEYDPTIEDSYRKQVVIDGETCLLDILDTAGQEEYSAMRDQYMRTGEGF
LCVFAINNTKSFEDIHQYREQIKRVKDSDDVPMVLVGNKCDLPARTVETRQAQDLARSYGIPYIETSAKTRQGVEDAF
YTLVREIRQHKLRKLNPPDESGPGCMNCKCVIS

Chicken.NRAS NP_001012567.1 GTPase NRas [Gallus gallus]
MTEYKLVVVGAGGVGKSALTIQLIQNHFVDEYDPTIEDSYRKQVVIDGETCLLDILDTAGQEEYSAMRDQYMRTGEGF
LCVFAINNSKSFADINLYREQIKRVKDSDDVPMVLVGNKCDLPTRTVDTKQAQELAKSYGIPFIETSAKTRQGVEDAF
YTLVREIRQYRMKKLNSNEDGNQGCMGLSCIVM

Chicken.KRAS4A XP_015145733.1 GTPase KRas isoform X1 [Gallus gallus]
MTEYKLVVVGAGGVGKSALTIQLIQNHFVDEYDPTIEDSYRKQVVIDGETCLLDILDTAGQEEYSAMRDQYMRTGEGF
LCVFAINNTKSFEDIHHYREQIKRVKDSEDVPMVLVGNKCDLPSRTVDTKQAQDLARSYGIPFIETSAKTRQRVEDAF
YTLVREIRQYRVKKISKEEKTPGCMKIKKCLVM

Lizard.KRASBL ref|XP_033027004.1|:1-186 GTPase HRas-like [Lacerta agilis]
MTEYKLVVVGAGGVGKSALTIQLIQNHFVDEYDPTIEDSYRKQVVIDGETCLLDILDTAGQEEYSAMRDQYMRTGEGF
LCVFAINNTKSFEDVHHYREQINRVKDSDDVPMVLVGNKCDLPSRTVDTKQAQELARSYGIPFVETSAKTRQGVEDAF
YTLVREIRKHKEKISNGRKKKSSKRKCIIL

1.1.4 AMPHIBIANS

Frog.KRASBL NP_001008034.1 Kirsten rat sarcoma viral oncogene homolog [Xenopus tropicalis]
MTEYKLVVVGAGGVGKSALTIQLIQNHFVDEYDPTIEDSYRKQVVIDGETCLLDILDTAGQEEYSAMRDQYMRTGEGF
LCVFAINNTKSFEDVHHYREQINRVKDSDDVPMVLVGNKCDLPSRTVDTKQAQELAKSYGIPFIETSAKTRQGVEDAF
YTLVREIRKHKEKISNGKKKKSSKRKCVIL

1.1.5 LOBE-FINNED FISHES (Sarcopterygii)

Coelacanth.KRASBL XP_014344273.1 PREDICTED: GTPase HRas-like [Latimeria chalumnae]
MTEYKLVVVGAGGVGKSALTIQLIQNHFVDEYDPTIEDSYRKQVVIDGETCLLDILDTAGQEEYSAMRDQYMRTGEGF
LCVFAINNVKSFEDVHHYREQINRVKDSDDVPMVLVGNKCDLPSRTVDTKQAQDLAKSYGIPVIETSAKTRQGVEDAF
YTLVREIRRHKEKISKNGKKRRKKSSKRKCTIL

1.1.6 RAY FINNED FISHES (Actinopterygii)

Reedfish.KRAS4B XP_028671431.1 ENSECRT00000008296.1 GTPase KRas isoform X2 [Erpetoichthys calabaricus]
MTEYKLVVVGAGGVGKSALTIQLIQNHFVDEYDPTIEDSYRKQVVIDGETCLLDILDTAGQEEYSAMRDQYMRTGEGF
LCVFAINNTKSFEDIHHYREQIKRVKDSEDVPMVLVGNKCDLPSRTVDTKQAQDLARSYAIPFIETSAKTRQGVDDAF
YTLVREIRKHKEKMSKDGKKKKKKSKPRCILM

Reedfish.KRASBL XP_028669532.1 GTPase HRas-like [Erpetoichthys calabaricus]
MTEYKLVVVGAGGVGKSALTIQLIQNHFVDEYDPTIEDSYRKQVVIDGETCLLDILDTAGQEEYSAMRDQYMRTGEGF
LCVFAINNLKSFEDVHLYREQINRVKDSDDVPMVLVGNKCDLPSRTVETKPAQELAKSYGIPFVETSAKTRQGVEDAF
YTLVREIRKHKEKVSRNGKKKTSKRRCTIL

Reedfish.HRAS XP_028649344.1 ENSECRT00000002289.1GTPase HRas [Erpetoichthys calabaricus]
MTEYKLVVVGAGGVGKSALTIQLIQNHFVDEYDPTIEDSYRKQVVIDGETCLLDILDTAGQEEYSAMRDQYMRTGEGF
LCVFAINNTKSFEDIHQYREQIKRVKDSDDVPMVLVGNKCDLPARTVDTRQAQELARSYGIPYIETSAKTRQGVEDAF
YTLVREIRQHKLRKLNPPDESGQDCISCKCVIS

Reedfish.NRAS 6 XP_028652286.1 ENSECRT00000009742.1GTPase NRas [Erpetoichthys calabaricus]
MTEYKLVVVGAGGVGKSALTIQLIQNHFVDEYDPTIEDSYRKQVVIDGETCLLDILDTAGQEEYSAMRDQYMRTGEGF
LCVFAINNSKSFADIHLYREQIKRVKDSDDVPMVLVGNKCDLPTRTVDTKQAQELARSYGIEFVETSAKTRQGVEDAF
YTLVREIRHYRMKKLNSSEERNQGCLGVSCTVM

Reedfish.KRAS4A XP_028671408.1 ENSECRT00000008303.1 GTPase KRas isoform X1 [Erpetoichthys calabaricus]
MTEYKLVVVGAGGVGKSALTIQLIQNHFVDEYDPTIEDSYRKQVVIDGETCLLDILDTAGQEEYSAMRDQYMRTGEGF
LCVFAINNTKSFEDIHHYREQIKRVKDSEDVPMVLVGNKCDLPSRTVDTKQAQDLARSYAIPFIETSAKTRQRVEDAF
YTLVREIRQYRLKKFSKEEKTPRCVKLKKCVVM

1.1.7 CARTILAGINOUS FISHES (Chondrichthyes)

1.1.7.1 Holocephali

Shark.KRAS4B XP_007907218.1 PREDICTED: GTPase KRas isoform X1 [Callorhinchus milii]
MTEYKLVVVGAGGVGKSALTIQLIQNHFVDEYDPTIEDSYRKQVVIDGETCLLDILDTAGQEEYSAMRDQYMRTGEGF
LCVFAINNTKSFEDIHHYREQIKRVKDSDDVPMVLVGNKCDLPSRTVDTRQAQELARSYGIPFIETSAKTRQGVDDAF
YTLVREIRKHKEKMSKDGKKKKNKTKKKCSIM

shark.KRASBL NP_001279499.1 ENSCMIG00000018631 GTPase HRas-like [Callorhinchus milii]
MTEYKLVVVGAGGVGKSALTIQLIQNHFVDEYDPTIEDSYRKQVVIDGETCLLDILDTAGQEEYSAMRDQYMRTGEGF
LCVFAINNTKSFEDVHLYREQINRVKDSDDVPMVLVGNKCDLPSRTVDTKQAQELAKSYGIPFIETSAKTRQGVEDAF
YTLVREIRKYKEKISKNGKKKKKPSKKKCIIL

Shark.HRAS XP_007907535.1 PREDICTED: GTPase HRas [Callorhinchus milii] ENSCMIT00000047211.1
MTEYKLVVVGAGGVGKSALTIQLIQNHFVDEYDPTIEDSYRKQVVIDGETCLLDILDTAGQEEYSAMRDQYMRTGEGF
LCVFAINNTKSFEDIHQYREQIKRVKDSDDVPMVLVGNKCDLPARTVEARQAQDLARSYGIPYIETSAKTRQGVEDAF
YTLVREISQHKVRKSNPPDDSGRDCSNCKCVIL

Shark.NRAS XP_007908417.1 PREDICTED: GTPase NRas [Callorhinchus milii]
MTEYKLVVVGAGGVGKSALTIQLIQNHFVDEYDPTIEDSYRKQVVIDGETCLLDILDTAGQEEYSAMRDQYMRTGEGF
LCVFAINNSKSFADIHLYREQIKRVKDSEDVPMVLVGNKCDLPARTVDTKQAQELARSYGIPFIETSAKTRQGVEDAF
YTLVREIRQYRTIKLNSQDDRNQGCLGLKCIIM

Shark.KRAS4A XP_007907220.1 PREDICTED: GTPase KRas isoform X2 [Callorhinchus milii]
MTEYKLVVVGAGGVGKSALTIQLIQNHFVDEYDPTIEDSYRKQVVIDGETCLLDILDTAGQEEYSAMRDQYMRTGEGF
LCVFAINNTKSFEDIHHYREQIKRVKDSDDVPMVLVGNKCDLPSRTVDTRQAQELARSYGIPFIETSAKTRQRVEDAF
YTLVREIRQYRLRKLNTEEKTTRCVVFKCVVM

Shark.MRAS XP_007894456.1 PREDICTED: ras-related protein M-Ras [Callorhinchus milii]
MATSAVPSDNLPTYKLVVVGDGGVGKSALTIQFFQKIFVPDYDPTIEDSYLKHTEIDGQWAILDVLDTAGQEEFSAMR
EQYMRTGDGFLIVFSVTDKASFEHVDRFHQLILRVKDRESFPMILVANKVDLVHLRKVTSEQGREMAAKYNIPYIETS
AKDPPLNVDKAFHDLVRVIRQQIPEKSQKKKKAKWRGDRTAGSNKLQCSVM

Shark.TC21/RRAS2 XP_007885725.1 PREDICTED: ras-related protein R-Ras2 [Callorhinchus milii]
MAAAKDGGAAQEKFKLVVVGGGGVGKSALTIQFIQSYFVTDYDPTIEDSYTKQCVIDDRAARLDILDTAGQEEFGAMR
EQYMRTGEGFLLVFSVTDRGSFEEIYKFQRQILRVKDRDEFPMILVGNKADLEHQRQVTLEEGQQLARQLKVTYMEAS
AKIRLNVDQAFHQLVRVIRRFQEQECPPSPEPSRNEKDRSGCHCVLF

1.1.7.2 Helasmobranchii

SkateKRAS4B skate XP_032895339.1 GTPase KRas isoform X1 [Amblyraja radiata]
MTEYKLVVVGAGGVGKSALTIQLIQNHFVDEYDPTIEDSYRKQVVIDGETCLLDILDTAGQEEYSAMRDQYMRTGEGF
LCVFAINNSKSFEDIHHYREQIKRVKDSEDVPMVLVGNKCDLPSRTVDTKQAQDLARSYTIPFIETSAKTRLGVDDAF
YTLVREIRKHKEKMSKDGKKKKNKSKKKCLMM

Skate.KRASBL XP_032869079.1 GTPase HRas-like [Amblyraja radiata]
MTEYKLVVVGAGGVGKSALTIQLIQNHFVDEYDPTIEDSYRKQVVIDGETCLLDILDTAGQEEYSAMRDQYMRTGEGF
LCVFAINNIKSFEDVHLYREQINRVKDSDDVPMVLVGNKCDLPSRTVDTKQAQELAKGYGIPFIETSAKTRQGVEDAF
YTLVREIRKYKEKISKNGKRKKKNSKKRCIIL

Skate.Hras XP_032894766.1 GTPase HRas [Amblyraja radiata]
MTEYKLVVVGAGGVGKSALTIQLIQNHFVDEYDPTIEDSYRKQVVIDGETCLLDILDTAGQEEYSAMRDQYMRTGEGF
LCVFAINNTKSFEDIHQYREQIKRVKDSDDVPMVLVGNKCDLPSRTVETRQAQDLARSYGIPYIETSAKTRQGVEDAF
YTLVREISQHKVRKSNPPDESGQDCNNCKCVLL

Skate.NRAS XP_032898682.1 GTPase NRas [Amblyraja radiata]

MTEYKLVVVGAGGVGKSALTIQLIQNHFVDEYDPTIEDSYRKQVVIDGETCLLDILDTAGQEEYSAMRDQYMRTGEGF
LCVFAINNHKSFADVHLYREQIKRVKDSEDVPMVLVGNKCDLPSRTVDTKQAQELARSYGIPFIETSAKTRQGVEDAF
YTLVREIRQYRTIKLNSQDDRNQGCLGLKCIIM

Skate.KRAS4A XP_032895340.1 GTPase KRas isoform X2 [Amblyraja radiata]

MTEYKLVVVGAGGVGKSALTIQLIQNHFVDEYDPTIEDSYRKQVVIDGETCLLDILDTAGQEEYSAMRDQYMRTGEGF
LCVFAINNSKSFEDIHHYREQIKRVKDSEDVPMVLVGNKCDLPSRTVDTKQAQDLARSYTIPFIETSAKTRLRVEDAF
YTLVREIRQYRLRKLNVEEKTARCVPFKCIMM

1.1.8 JAWLESS FISH (Cyclostomata)

1.1.8.1 Myxini; Myxiniformes;

Hagfish.KRAS ENSEBUT00000024928.1 FYBX02009586.1 (Eptatretus burgeri)

MTEYKLVVVGAGGVGKSALTIQLIQNHFVDEYDPTIEDSYRKQVVIDGETCLLDILDTAGQEEYSAMRDQYMRTGEGF
LCVFAVNNSKSFDDIHLYREQIKRVKDSEDVPMVLVGNKCDLPTRTVDTKQALDLARSFGIPCIETSAKTRQGVDDAF
YTLVREIRKYKERASKDGKKKKKRSRKMCLVM

Hagfish.HRAS ENSEBUT00000007591.1 FYBX02010718.1 (Eptatretus burgeri)
MTEYKLVVVGAGGVGKSALTIQLIQNHFVDEYDPTIEDSYRKQVVIDSETCLLDILDTAGQEEYSAMRDQYMRTGEGF
LCVFAINNTKSFEDIHQYREQIKRVKDSDDVPMVLVGNKCDLPARTVDSRQAQDLARSYNIPYVETSAKTRQGVEDAF
YTLVREIRDHKARKLHNMEDNGGGCNMCSCVIS

1.1.8.2 Hyperoartia; Petromyzontiformes.

Lamprey.KRAS XP_032834078. GTPase HRas [Petromyzon marinus] curated.
MTEYKLVVVGAGGVGKSALTIQLIQNHFVDEYDPTIEDSYRKQVVIDGETCLLDILDTAGQEEYSAMRDQYMRTGEGF
LCVFAINNSKSFEDIHQYREQIKRVKDSEDVPMVLVGNKCDLPTRTVDTKQAQELARSYGIPFIETSAKTRQGVDDAF
YTLVREIRKYKDRVSKDGRKKKKKSKKKCLLM

Lamprey.HRAS XP_032815672.1 ENSPMAT00000005016.GTPase HRas-like [Petromyzon marinus]
MTEYKLVVVGAGGVGKSALTIQLIQNHFVDEYDPTIEDSYRKQVVIDSETCLLDILDTAGQEEYSAMRDQYMRTGEGF
LCVFAINNSKSFEDIHQYREQIKRVKDSDDVPMVLVGNKCDLPARTVDTKQAQDLARSYGIPYVETSAKTRQGVEDAF
YTLVREIRDHKLRKMNNANNKDECSVCSCVLS

Lamprey.KRAS Entosphenus tridentatus Pacific Lamprey
https://genomes.stowers.org/chado/organism profiling
MTEYKLVVVGAGGVGKSALTIQLIQNHFVDEYDPTIEDSYRKQVVIDGETCLLDILDTAGQEEYSAMRDQYMRTGEGF
LCVFAINNSKSFEDIHQYREQIKRVKDSEDVPMVLVGNKCDLPTRTVDTKQAQELARSYGIPFIETSAKTRQGVDDAF
YTLVREIRKYKDRVSKDGRKKKKKSKRKCVLM

Lamprey.HRAS Entosphenus tridentatus Pacific Lamprey
https://genomes.stowers.org/chado/organism profiling
MTEYKLVVVGAGGVGKSALTIQLIQNHFVDEYDPTIEDSYRKQVVIDSETCLLDILDTAGQEEYSAMRDQYMRTGEGF
LCVFAINNSKSFEDIHQYREQIKRVKDSDDVPMVLVGNKCDLPARTVDTKQAQDLARSYGIPYVETSAKTRQGVEDAF
YTLVREIRDHKLRKMNNANNKDECSVCSCVLS

1.2 CEPHALOCHORDATA

Lancelet1_Bf XP_035672300.1 GTPase HRas-like [Branchiostoma floridae]
MTEYKLVVVGDGGVGKSALTIQLIQNHFVQEYDPTIEDSYRKQVVIDGETCLLDILDTAGQEEYSAMRDQYMRTGEGF
LCVFAINNDKSFEDIAMYREQIKRVKDSDEVPIVLVGNKCDLPARTVENKQAWTLAKSYGVPYVETSAKTRMGVDDAF
YTLVREIRAYKERQGKAGKKKKNFKSRCTIL

Lancelet1_Bb XP_019640053.1 PREDICTED: GTPase HRas-like isoform X1 [Branchiostoma belcheri]

MTEYKLVVVGDGGVGKSALTIQLIQNHFVQEYDPTIEDSYRKQVVIDGETCLLDILDTAGQEEYSAMRDQYMRTGEGF
LCVFAINNDKSFEDIAMYREQIKRVKDSDEVPIVLVGNKCDLPARTVENKQAFALAKSYGVPYVETSAKTRMGVDDAF
YTLVREIRAYKERQGKAGKKKKKNFKSRCTIL

Lancelet2_Bf XP_035696597.1 GTPase HRas [Branchiostoma floridae]
MTEYKLVVVGAGGVGKSALTIQLIQNHFVDEYDPTIEDSYRKQVVIDGETCLLDILDTAGQEEYSAMRDQYMRTGEGF
LCVFALNNTKSFEDISMYREQIKRVKDSEQVPMVLVGNKADLPQRVIDNKQAQDMAKSYSIPYVETSAKTRLGVEDAF
YTLVREIRMDKANRQATKRQGKDGTSGTCCVLL

Lancelet2_Bb XP_019642994.1 PREDICTED: GTPase HRas [Branchiostoma belcheri]
MTEYKLVVVGAGGVGKSALTIQLIQNHFVDEYDPTIEDSYRKQVVIDGETCLLDILDTAGQEEYSAMRDQYMRTGEGF
LCVFALNNTKSFEDISMYREQIKRVKDSEQVPMVLVGNKADLPQRVVDNKQAQDMAKSYSIPYVETSAKTRLGVEDAF
YTLVREIRMDKANRQASGKDGSSSSGTCCVLL

1.3.1 HEMICHORDATA

Acorn_Worm hemichordate XP_002736858.1 PREDICTED: GTPase KRas-like [Saccoglossus kowalevskii] PARTIAL first 2 exon missing.
FVDEYDPTIEDSYRKQVVIDGETCLLDILDTAGQEEYSAMRDQYMRTGEGFLCVFAVNNAKSFEDIGSYREQIKRVKD
ADDVPMVLVGNKCDLPTRAVDLKEANTLAKSYGIPFIETSAKTRQGVDDAFYTLVREIRKYKERRGNPQKKPGRRRFK
CSIL

1.3.2 ECHINODERMATA

1.3.2.1 Crinoidea

Crinoid XP_033102378.1 GTPase HRas [Anneissia japonica]
MTEYKLVVVGAGGVGKSALTIQLIQNHFVDEYDPTIEDSYRKQVVIDGETCLLDILDTAGQEEYSAMRDQYMRTGEGF
LCVYAVNNEKSFEDIHSYREQIQRVKDSEEVPMVLVGNKIDLPNRNVDQKSAGQLAKTFGIPYIETSAKTRQGVDEAF
YTLVREIRKDKEKRNISKNKKPSRRKCCLF

1.3.2.2.1 Echinozoa

Sea_urchin echinoderm XP_030848609.1 GTPase HRas [Strongylocentrotus purpuratus]
MTEYKLVVVGAGGVGKSALTIQLIQNHFVDEYDPTIEDSYRKQVVIDGETCLLDILDTAGQEEYSAMRDQYMRTGEGF
LCVFAINNEKSFEDVNLYREQIKRVKDAEEVPMVLVGNKIDLPNWAVQTRQGLSLAKSYGIPYIETSAKTRQGVDDAF
YTLVREIRKDKDRKNASNKKGKRKKRVCILF

Sea_Urchin.1 XP_030848582.1 GTPase HRas [Strongylocentrotus purpuratus]
MTEYKLVVVGAGGVGKSALTIQLIQNHFVDEYDPTIEDSYRKQVVIDGETCLLDILDTAGQEEYSAMRDQYMRTGEGF
LCVFAINNEKSFEDVNLYREQIKRVKDAEEVPMVLVGNKIDLPNWAVQTRQGLSLAKSYGIPYIETSAKTRQGVDDAF
YTLVREIRKDKDRKNASNKKGKRKKRGCILF

Green_sea_urchin XP_041467031.1 GTPase HRas [Lytechinus variegatus]
MTEYKLVVVGAGGVGKSALTIQLIQNHFVDEYDPTIEDSYRKQVVIDGETCLLDILDTAGQEEYSAMRDQYMRTGEGF
LCVFAINNEKSFEDVNLYREQIKRVKDAEEVPMVLVGNKIDLPNWAVNTKQGMTLAKSYGIPYIETSAKTRQGVDDAF
YTLVREIRKDKDRKNASNKKGKSKKRSCILF

1.3.2.2.2 Asterozoa

Common_starfish XP_033638627.1 GTPase HRas isoform X2 [Asterias rubens]
MTEYKLVVVGAGGVGKSALTIQLIQNHFVDEYDPTIEDSYRKQVVIDGETCLLDILDSAGQEEYSAMRDQYMRTGEGF
LIVFAVNNRKSFEDFNLYWSQIYRVKDAEEVPMVLVGNKIDLSDRTVEARDGAATAKSYGIPYIETSAKTRHGVDDAF
YTLVREIRKDKERRNVSKKKKKKSKGMKCVLL

Bat_starfish XP_038052655.1 GTPase HRas isoform X2 [Patiria miniata]
MTEYKLVVVGAGGVGKSALTIQLIQNHFVDEYDPTIEDSYRKQVVIDGETCLLDILDSAGQEEYSAMRDQYMRTGEGF
LIVFAVNNRKSFEDFNLYWSQIFRVKDTDEVPMVLVGNKIDLPERTVDTLDGLAMAKSSGIPYIETSAKTRHGVDDAF
YTLVREIRKDKERRNASKKRKKKSKGKCVLL

2. PROTOSTOMES

2.1 Ecdysozoa; Arthropoda;

Dhrosophila Ras85D FBtr0082122 NP_476699.1 Ras oncogene at 85D [Drosophila melanogaster]
MTEYKLVVVGAGGVGKSALTIQLIQNHFVDEYDPTIEDSYRKQVVIDGETCLLDILDTAGQEEYSAMRDQYMRTGEGF
LLVFAVNSAKSFEDIGTYREQIKRVKDAEEVPMVLVGNKCDLASWNVNNEQAREVAKQYGIPYIETSAKTRMGVDDAF
YTLVREIRKDKDNKGRRGRKMNKPNRRFKCKML

2.2 Ecdysozoa; Nematoda;

C.elegans.let60 ZK792.6.1 NP_502213.3 Ras protein let-60 [Caenorhabditis elegans]
MTEYKLVVVGDGGVGKSALTIQLIQNHFVEEYDPTIEDSYRKQVVIDGETCLLDILDTAGQEEYSAMRDQYMRTGEGF
LLVFAVNEAKSFENVANYREQIRRVKDSDDVPMVLVGNKCDLSSRSVDFRTVSETAKGYGIPNVDTSAKTRMGVDEAF
YTLVREIRKHRERHDNNKPQKKKKCQIM

3. CNIDARIANS

Sea_anemone XP_001625014.1 GTPase HRas [Nematostella vectensis]
MTEYKLVVVGAGGVGKSALTIQLIQNHFVDEYDPTIEDSYRKQVVIDGETCLLDILDTAGQEEYSAMRDQYMRTGEGF
LCVFAVNNSKSFEDINQYREQIKRVKDAEEVPMVLVGNKCDLPQRTVSTSDAQELAKSYNIPFQETSAKTRQGVDDAF
YTLVREIRKDKEKRGPPKPKKSKKKCIIL

Sea_anemone XP_031548789.1 GTPase HRas [Actinia tenebrosa]
MTEYKLVVVGAGGVGKSALTIQLIQNHFVDEYDPTIEDSYRKQVVIDGETCLLDILDTAGQEEYSAMRDQYMRTGEGF
LCVFAVNNRKSFEDINQYREQIKRVKDAEEVPMVLVGNKCDLPQRTVSTGDAQELAKSYGIPFQETSAKTRQGVDDAF
YTLVREIRKDKEKRGPPKKDSKKKKCIIL

Sea_anemoneEd XP_020898624.1 GTPase HRas [Exaiptasia diaphana]
MTEYKLVVVGAGGVGKSALTIQLIQNHFVDEYDPTIEDSYRKQVVIDGETCLLDILDTAGQEEYSAMRDQYMRTGEGF
LCVFAVNNRKSFEDINQYREQIKRVKDAEEVPMVLVGNKCDLPQRTVSTADAQELAKSYGIPFQETSAKTRQGVDDAF
YTLVREIRKDKEKRGPPKRPKQKKKCVIL

Soft_coral XP_028399623.1 GTPase HRas [Dendronephthya gigantea]
MTEYKLVVVGAGGVGKSALTIQLIQNHFVDEYDPTIEDSYRKQVVIDGETCLLDILDTAGQEEYSAMRDQYMRTGEGF
LCVFAINNVKSFDDVHQYREQIKRVKDADEVPMVLVGNKCDLPTRNVSSESARELASSFSIPFIETSAKTRQGVDEAF
YTLVREIRKDKDRKGQMKPKPKKKRKCIIL

4. PORIFERANS

Sponge.Aq XP_003384227.1 PREDICTED: GTPase HRas [Amphimedon queenslandica]
MTEYKLVVVGAGGVGKSALTIQLIQNHFVDEYDPTIEDSYRKQVVIDGDTCLLDILDTAGQEEYSAMRDQYMRTGEGF
LCVFAVDNMKSFEDVESYRAQIRRVKDSNDVPIILVGNKIDLPRREVDQKIAQGFVKNHNMAGYIETSAKTRQGVDDA
FYSLVREIRRYKDERSSQTPAEKPKKKMKCNLL

Sponge.Sd ABD65420.1 K-ras2 [Suberites domuncula]NO genomic sequence
MTEYKLVVVGAGGVGKSALTIQLIQNHFVDEYDPTIEDSYRKQVVIDGETCLLDILDTAGQEEYSAMRDQYMRTGEGF
LCVFAIDNMKSFEDIDSYRGQIRRVKDADDIPMILVGNKIDLPRREVDPKLAQSYGKNHKMPYIETSAKTRQGVDDAF
YSLVREIRRWKETKTDEANTKKTKKKRVCKVF

5. CHOANOFLAGELLATA

Salpingoeca XP_004993374.1_ras protein [Salpingoeca rosetta]

MTEYRLVVVGTGGVGKSALTIQLIQQHFVTEYDPTIEDSYRKHVSIDDEACLLDILDTAGQEDYSAMRDQYMRTGEGF
LCVYSIDSQQSLDEIHSFREQILRVKDQDEVPMILVGNKCDLEEHREVSTEAGQAVAKSYSIPFMETSAKKRINVEEA
FYQLVREIRKYKKKHNPEGGAAPKKKKFCTLL

6. FILASTEREA

Capsaspora XP_004365165.1 ras family protein [Capsaspora owczarzaki ATCC 30864]
MTEYKLVVVGGGGVGKSALTIQLIQNHFVDEYDPTIEDSYRKQVVIDDETCLLDILDTAGQEEYSAMRDQYMRTGEGF
LCVYAITSRPSFEEIHPFREQILRVKDADEVPMILVANKCDLESDRQVSHTEGQATAKQFGIPFMETSAKTRTRVEDA
FYTLVREIRRFKKRNASSDTPKKKKAKCLLL

7. FUNGI

Yeast_SpRAS1 NP_593579.1 GTPase Ras1 Ras1p [Schizosaccharomyces pombe]
MRSTYLREYKLVVVGDGGVGKSALTIQLIQSHFVDEYDPTIEDSYRKKCEIDGEGALLDVLDTAGQEEYSAMREQYMR
TGEGFLLVYNITSRSSFDEISTFYQQILRVKDKDTFPVVLVANKCDLEAERVVSRAEGEQLAKSMHCLYVETSAKLRL
NVEEAFYSLVRTIRRYNKSEEKGFQNKQAVQTAQVPASTAKRASAVNNSKTEDEVSTKCCVIC

Yeast_ScRAS1 sp|P01119|RAS1_YEAST Ras-like protein 1 OS=Saccharomyces cerevisiae (strain ATCC 204508 / S288c) OX=559292 GN=RAS1 PE=1 SV=2
MQGNKSTIREYKIVVVGGGGVGKSALTIQFIQSYFVDEYDPTIEDSYRKQVVIDDKVSILDILDTAGQEEYSAMREQY
MRTGEGFLLVYSVTSRNSFDELLSYYQQIQRVKDSDYIPVVVVGNKLDLENERQVSYEDGLRLAKQLNAPFLETSAKQ
AINVDEAFYSLIRLVRDDGGKYNSMNRQLDNTNEIRDSELTSSATADREKKNNGSYVLDNSLTNAGTGSSSKSAVNHN
GETTKRTDEKNYVNQNNNNEGNTKYSSNGNGNRSDISRGNQNNALNSRSKQSAEPQKNSSANARKESSGGCCIIC

Yeast_ScRAS2 sp|P01120|RAS2_YEAST Ras-like protein 2 OS=Saccharomyces cerevisiae (strain ATCC 204508 / S288c) OX=559292 GN=RAS2 PE=1 SV=4
MPLNKSNIREYKLVVVGGGGVGKSALTIQLTQSHFVDEYDPTIEDSYRKQVVIDDEVSILDILDTAGQEEYSAMREQY
MRNGEGFLLVYSITSKSSLDELMTYYQQILRVKDTDYVPIVVVGNKSDLENEKQVSYQDGLNMAKQMNAPFLETSAKQ
AINVEEAFYTLARLVRDEGGKYNKTLTENDNSKQTSQDTKGSGANSVPRNSGGHRKMSNAANGKNVNSSTTVVNARNA
SIESKTGLAGNQATNGKTQTDRTNIDNSTGQAGQANAQSANTVNNRVNNNSKAGQVSNAKQARKQQAAPGGNTSEASK
SGSGGCCIIS

Aspergillus_RASA XP_753526.1 RAS small monomeric GTPase RasA [Aspergillus fumigatus Af293]
MASKFLREYKLVVVGGGGVGKSCLTIQLIQSHFVDEYDPTIEDSYRKQCVIDDEVALLDVLDTAGQEEYSAMREQYMR
TGEGFLLVYSITSRQSFEEIMTFQQQILRVKDKDYFPIIVVGNKCDLEKERAVSQQEGEALARQFGCKFIETSAKSRI
NVENAFYDLVREIRRYNKEMSSYPSGSGAAGTRAPEGKMDVSEPGDNAGCCGKCVIM

8. APUSOZOA

Thecamonas XP_013760036.1 Ras-like protein rasG [Thecamonas trahens ATCC 50062] exón phase 1
MTEYKLVVVGGGGVGKSALTIQLIQNHFVDEYDPTIEDSYRKQVIIDQETCLLDILDTAGQEEYSAMRDQYMRTGEGF
LCVYAITSRSSFEEITAFREQILRVKDADAVPIMICGNKCDLESERQVSAAEGEELAKSFTVPFLETSAKTRVNVEEA
FFQLVREIRRMKKDDSAGGKPAGKKKKCLIL

9. AMOEBOZOA

Dictyostelium slimeMold XP_629338.1 Ras GTPase [Dictyostelium discoideum AX4]
MTEYKLVIVGGGGVGKSALTIQLIQNHFIDEYDPTIEDSYRKQVSIDDETCLLDILDTAGQEEYSAMRDQYMRTGQGF
LCVYSITSRSSYDEIASFREQILRVKDKDRVPLILVGNKADLDHERQVSVNEGQELAKGFNCPFMESSAKSRINVEEA
FYSLVREIRKELKGDQSSGKAQKKKKQCLIL

G-DOMAINS DNA SEQUENCES

human.KRAS4B

ATGACTGAATATAAACTTGTGGTAGTTGGAGCTGGTGGCGTAGGCAAGAGTGCCTTGACGATACAGCTAATTCAGAATCATTTTGTGG
ACGAATATGATCCAACAATAGAGGATTCCTACAGGAAGCAAGTAGTAATTGATGGAGAAACCTGTCTCTTGGATATTCTCGACACAGC
AGGTCAAGAGGAGTACAGTGCAATGAGGGACCAGTACATGAGGACTGGGGAGGGCTTTCTTTGTGTATTTGCCATAAATAATACTAAA
TCATTTGAAGATATTCACCATTATAGAGAACAAATTAAAAGAGTTAAGGACTCTGAAGATGTACCTATGGTCCTAGTAGGAAATAAAT
GTGATTTGCCTTCTAGAACAGTAGACACAAAACAGGCTCAGGACTTAGCAAGAAGTTATGGAATTCCTTTTATTGAAACATCAGCAAA
GACAAGACAGGGTGTTGATGATGCCTTCTATACATTAGTTCGAGAAATTCGAAAA

chicken.KRAS4B

ATGACAGAGTATAAGCTTGTTGTCGTTGGAGCTGGTGGTGTGGGCAAGAGCGCCTTGACAATACAGCTCATTCAGAACCACTTTGTGG
ATGAGTATGACCCTACCATAGAGGATTCCTACAGAAAGCAAGTAGTAATTGATGGGGAAACCTGTCTCTTGGATATTCTTGATACAGC
AGGTCAAGAAGAATATAGTGCAATGAGGGACCAATATATGAGAACAGGAGAAGGCTTTCTGTGTGTTTTTGCTATAAACAATACAAAA
TCTTTTGAAGATATTCACCATTATAGGGAACAAATAAAGAGAGTTAAAGACTCTGAAGATGTCCCAATGGTGCTAGTAGGAAACAAAT
GTGATTTGCCTTCCAGAACAGTAGATACAAAACAAGCTCAGGATTTAGCAAGAAGTTATGGAATTCCTTTTATTGAAACATCAGCAAA
GACAAGACAGGGTGTTGATGATGCCTTCTATACATTAGTTCGAGAAATCAGAAAA

reedfish.KRAS4B

ATGACAGAATATAAGCTCGTTGTGGTTGGAGCTGGTGGTGTTGGGAAGAGTGCCTTGACCATACAGCTTATTCAAAACCATTTTGTGG
ATGAATATGACCCAACAATAGAGGACTCCTACAGAAAACAAGTAGTCATTGATGGGGAGACATGTTTGTTGGATATTCTCGACACTGC
AGGTCAAGAGGAATACAGTGCAATGAGAGATCAGTATATGCGTACAGGGGAGGGTTTTCTTTGCGTTTTTGCTATTAATAATACAAAG
TCATTTGAAGACATACATCATTATAGAGAACAAATAAAACGAGTGAAAGATTCAGAAGATGTCCCAATGGTTCTGGTAGGAAACAAAT
GTGATCTTCCCTCTCGAACTGTGGACACTAAGCAAGCTCAGGACCTAGCCAGAAGTTACGCAATTCCCTTTATAGAAACATCTGCCAA
AACCAGACAAGGAGTGGATGATGCCTTTTATACATTAGTAAGAGAAATTCGAAAA

shark.KRAS4B

ATGACAGAATACAAACTGGTGGTGGTTGGAGCTGGTGGCGTAGGGAAAAGTGCCCTGACAATACAGCTAATTCAGAACCATTTTGTAG
ATGAATATGATCCCACAATAGAAGATTCCTACAGAAAACAAGTAGTAATTGATGGGGAAACCTGTCTTTTAGATATTCTGGACACAGC
AGGTCAAGAGGAGTACAGCGCTATGAGAGATCAGTACATGAGGACAGGCGAGGGCTTCCTGTGTGTTTTTGCCATCAATAATACTAAA
TCATTCGAAGATATTCATCATTATAGAGAACAGATAAAAAGGGTAAAAGACTCTGATGACGTGCCAATGGTCCTCGTGGGAAATAAAT
GTGATTTGCCTTCAAGAACAGTAGACACAAGACAAGCACAGGAACTAGCGAGAAGTTACGGAATTCCATTCATTGAAACGTCAGCGAA
AACAAGACAAGGCGTTGACGACGCTTTCTACACACTAGTCCGCGAAATCCGAAAG

coelacanth.KRASL

ATGACGGAATACAAGCTGGTTGTGGTGGGTGCAGGCGGCGTAGGAAAAAGTGCTCTCACCATCCAGTTAATTCAGAATCATTTTGTGG
ATGAATATGACCCCACCATAGAGGACTCGTACAGGAAGCAGGTGGTGATTGATGGAGAGACATGTCTGTTGGACATCCTGGACACGGC
CGGTCAAGAGGAGTACAGTGCCATGCGTGATCAGTACATGCGCACAGGAGAAGGTTTCCTCTGTGTTTTCGCCATCAACAATGTCAAG
TCCTTTGAAGACGTTCACCATTACAGGGAGCAGATAAACCGAGTGAAGGACTCAGACGATGTTCCAATGGTTCTGGTGGGGAACAAGT
GTGACCTCCCATCTCGGACGGTGGACACCAAACAGGCGCAGGATCTGGCCAAGAGCTATGGGATCCCTGTCATAGAAACTTCAGCCAA
AACCAGACAGGGGGTGGAGGACGCTTTTTACACACTCGTGCGAGAAATTCGCCGG

xenopus.KRASL

ATGACTGAGTACAAACTGGTGGTGGTCGGTGCTGGAGGCGTAGGGAAGAGCGCTCTCACAATCCAGCTCATTCAGAACCATTTTGTGG
ACGAATATGATCCTACTATTGAGGACTCCTACAGAAAGCAGGTGGTGATAGACGGGGAGACGTGCCTCCTGGATATATTGGACACGGC
GGGACAAGAGGAATACAGCGCCATGAGGGATCAGTACATGCGCACGGGAGAAGGGTTTCTCTGTGTCTTTGCTATTAATAACACAAAG
TCCTTCGAGGATGTCCATCATTACAGGGAACAGATTAACAGAGTAAAAGATTCCGATGATGTTCCCATGGTGTTAGTTGGTAACAAAT
GTGACCTCCCATCCCGGACTGTGGACACAAAGCAAGCACAAGAACTGGCAAAGAGCTACGGTATCCCTTTCATAGAAACCTCGGCCAA
AACCAGACAGGGAGTCGAAGACGCCTTCTATACCCTAGTCCGTGAAATCCGCAAG

reptile.KRASL

ATGACAGAATACAAACTGGTGGTGGTGGGTGCTGGTGGTGTTGGGAAGAGTGCATTGACTATACAGTTGATTCAGAATCACTTTGTGG
ACGAGTATGATCCCACTATAGAGGATTCATACCGGAAGCAAGTGGTAATTGATGGAGAGACGTGTCTCTTGGATATCTTGGACACAGC
TGGGCAAGAGGAGTACAGTGCTATGCGTGACCAGTACATGAGGACAGGAGAAGGCTTCCTCTGTGTCTTTGCAATCAACAACACAAAG
TCTTTCGAAGATGTTCACCACTACAGGGAGCAGATCAACCGTGTGAAGGACTCCGATGATGTTCCAATGGTGCTTGTTGGCAATAAAT

GTGATCTGCCCTCCCGGACGGTGGATACGAAACAAGCCCAGGAGCTTGCCAGAAGCTACGGAATCCCCTTTGTAGAGACCTCTGCCAA
AACCAGACAGGGAGTGGAAGATGCTTTCTACACCCTGGTGCGAGAAATCCGAAAA

reedfish.KRASL

ATGACGGAGTACAAGCTGGTGGTGGTTGGAGCAGGAGGCGTGGGAAAGAGTGCACTCACCATTCAACTTATTCAGAACCACTTTGTGG
ATGAATATGATCCTACTATCGAGGACTCTTACAGGAAGCAGGTTGTGATAGACGGGGAAACGTGTCTCCTGGACATTCTGGACACTGC
TGGTCAAGAAGAGTACAGCGCCATGAGAGATCAGTACATGCGGACTGGCGAGGGTTTCCTTTGTGTTTTTGCCATCAACAACCTCAAA
TCCTTTGAGGATGTGCACTTGTACAGAGAGCAGATTAACAGGGTCAAAGACTCAGATGATGTTCCCATGGTGCTTGTTGGTAACAAGT
GTGATTTGCCATCACGAACAGTGGAGACTAAACCTGCACAAGAGCTGGCAAAGAGCTATGGTATTCCGTTTGTGGAGACATCAGCTAA
AACAAGACAGGGGGTGGAAGATGCTTTTTACACTCTTGTGCGGGAAATCCGAAAG

shark.KRASL

ATGACAGAATACAAGCTGGTGGTGGTAGGAGCTGGTGGCGTTGGAAAGAGCGCGCTGACAATCCAACTAATACAGAACCATTTTGTGG
ATGAATACGACCCAACCATAGAGGACTCTTACAGGAAACAGGTGGTGATTGATGGGGAAACGTGTCTGTTGGATATTCTGGACACAGC
AGGTCAAGAGGAGTACAGTGCTATGAGAGATCAGTACATGCGCACCGGAGAGGGCTTCCTCTGTGTCTTCGCAATCAATAACACCAAA
TCCTTCGAGGATGTCCATCTGTATAGAGAACAAATTAATCGAGTGAAAGACTCCGATGATGTTCCTATGGTCTTAGTCGGAAACAAGT
GTGACTTGCCATCACGGACCGTGGATACGAAACAAGCGCAGGAACTAGCAAAGAGCTACGGGATCCCCTTCATCGAAACGTCAGCCAA
AACAAGACAGGGCGTGGAAGATGCCTTCTACACACTCGTGCGTGAGATTCGCAAG

human.HRAS

ATGACGGAATATAAGCTGGTGGTGGTGGGCGCCGGCGGTGTGGGCAAGAGTGCGCTGACCATCCAGCTGATCCAGAACCATTTTGTGG
ACGAATACGACCCCACTATAGAGGATTCCTACCGGAAGCAGGTGGTCATTGATGGGGAGACGTGCCTGTTGGACATCCTGGATACCGC
CGGCCAGGAGGAGTACAGCGCCATGCGGGACCAGTACATGCGCACCGGGGAGGGCTTCCTGTGTGTGTTTGCCATCAACAACACCAAG
TCTTTTGAGGACATCCACCAGTACAGGGAGCAGATCAAACGGGTGAAGGACTCGGATGACGTGCCCATGGTGCTGGTGGGGAACAAGT
GTGACCTGGCTGCACGCACTGTGGAATCTCGGCAGGCTCAGGACCTCGCCCGAAGCTACGGCATCCCCTACATCGAGACCTCGGCCAA
GACCCGGCAGGGAGTGGAGGATGCCTTCTACACGTTGGTGCGTGAGATCCGGCAG

chicken.HRAS

ATGACCGAGTACAAGCTGGTGGTAGTGGGAGCTGGAGGTGTCGGGAAGAGCGCTTTGACGATACAGCTCATTCAGAACCATTTTGTTG
ATGAGTACGACCCCACAATAGAGGATTCCTACAGAAAGCAAGTCGTCATCGATGGAGAGACCTGTTTGCTGGACATCCTGGATACGGC
GGGGCAGGAGGAGTACAGTGCCATGCGAGACCAGTACATGAGAACGGGGGAAGGATTCCTGTGCGTCTTTGCCATTAACAACACCAAG
TCCTTTGAGGACATCCACCAGTACAGGGAGCAGATCAAGAGGGTGAAAGACTCAGATGATGTCCCCATGGTGCTGGTGGGAAATAAAT
GTGATCTGCCAGCACGGACAGTGGAGACCCGGCAAGCGCAGGACCTGGCCCGGAGTTACGGGATCCCCTACATAGAAACGTCGGCCAA
AACCAGACAGGGCGTCGAAGATGCCTTCTATACCTTAGTGCGGGAGATCCGTCAG

reedfish.HRAS

ATGACCGAGTACAAGCTGGTGGTGGTGGGAGCTGGTGGCGTTGGGAAGAGCGCTTTGACCATCCAGCTGATACAGAACCATTTTGTGG
ATGAATACGACCCAACGATAGAGGATTCTTATAGAAAACAAGTAGTAATCGATGGGGAGACATGCTTGTTAGACATTTTGGACACTGC
GGGTCAAGAGGAGTACAGCGCAATGCGAGATCAGTACATGAGAACTGGAGAGGGCTTCCTGTGTGTCTTTGCCATCAATAACACCAAA
TCATTTGAGGACATTCATCAGTACAGGGAACAAATAAAAAGAGTGAAAGACTCTGACGACGTTCCCATGGTCCTTGTGGGAAACAAAT
GTGACCTGCCGGCGCGGACTGTGGACACGAGACAAGCTCAAGAGCTTGCAAGGAGCTACGGCATCCCGTATATAGAAACCTCAGCCAA
GACCAGACAGGGAGTAGAGGATGCCTTCTACACTTTGGTGCGGGAAATCCGGCAG

shark.HRAS

ATGACCGAGTACAAGCTGGTGGTGGTCGGGGCTGGAGGCGTAGGAAAGAGCGCTCTAACTATTCAGCTGATTCAGAACCACTTTGTGG
ACGAATACGATCCTACAATAGAAGATTCCTATAGAAAGCAGGTAGTTATTGACGGAGAGACGTGTTTGTTAGACATTCTGGATACAGC
GGGTCAAGAGGAGTACAGTGCGATGAGAGACCAGTACATGAGGACTGGCGAGGGCTTTCTGTGTGTCTTTGCAATTAACAATACAAAA
TCCTTTGAAGATATTCATCAGTACAGAGAACAGATAAAAAGGGTAAAGGATTCAGATGACGTCCCTATGGTGTTGGTGGGAAACAAAT
GTGACCTCCCAGCACGCACCGTGGAGGCACGACAGGCACAGGACCTGGCCCGGAGCTACGGGATTCCTTACATTGAAACCTCAGCCAA
AACGAGACAGGGAGTGGAGGATGCCTTTTATACCTTGGTTCGTGAGATCAGCCAA

human.NRAS

ATGACTGAGTACAAACTGGTGGTGGTTGGAGCAGGTGGTGTTGGGAAAAGCGCACTGACAATCCAGCTAATCCAGAACCACTTTGTAG
ATGAATATGATCCCACCATAGAGGATTCTTACAGAAAACAAGTGGTTATAGATGGTGAAACCTGTTTGTTGGACATACTGGATACAGC
TGGACAAGAAGAGTACAGTGCCATGAGAGACCAATACATGAGGACAGGCGAAGGCTTCCTCTGTGTATTTGCCATCAATAATAGCAAG
TCATTTGCGGATATTAACCTCTACAGGGAGCAGATTAAGCGAGTAAAAGACTCGGATGATGTACCTATGGTGCTAGTGGGAAACAAGT
GTGATTTGCCAACAAGGACAGTTGATACAAAACAAGCCCACGAACTGGCCAAGAGTTACGGGATTCCATTCATTGAAACCTCAGCCAA
GACCAGACAGGGTGTTGAAGATGCTTTTTACACACTGGTAAGAGAAATACGCCAG

chicken.NRAS

ATGACTGAATACAAGCTGGTGGTGGTGGGAGCTGGCGGCGTCGGGAAGAGCGCGTTGACCATCCAGCTCATCCAGAACCACTTCGTGG
ACGAGTACGACCCCACCATCGAGGATTCGTACAGAAAGCAGGTTGTCATCGATGGAGAGACGTGCTTGTTGGACATTCTGGACACTGC
AGGACAGGAAGAATACAGTGCTATGCGTGATCAGTACATGAGAACTGGGGAAGGATTCCTTTGTGTGTTTGCCATTAACAACAGTAAA
TCATTCGCTGATATTAACCTTTACAGAGAGCAAATCAAGAGAGTGAAAGATTCAGACGATGTGCCAATGGTGCTGGTGGGGAATAAGT
GCGATTTGCCAACAAGGACAGTAGACACCAAACAGGCTCAAGAGTTAGCAAAAAGCTACGGCATTCCCTTCATAGAGACATCAGCCAA
AACGAGACAGGGTGTGGAAGATGCGTTTTACACACTGGTGAGGGAGATTCGGCAG

reedfishNRAS

ATGACTGAATATAAACTTGTGGTTGTGGGAGCTGGTGGTGTTGGGAAAAGTGCCTTGACAATCCAACTCATTCAGAATCACTTTGTGG
ATGAATATGATCCTACAATTGAGGACTCTTACAGGAAGCAAGTAGTAATTGATGGAGAGACCTGTTTATTGGATATTCTTGACACTGC
AGGACAAGAGGAGTACAGTGCAATGAGGGATCAATACATGCGAACAGGAGAGGGCTTCCTCTGTGTGTTTGCAATCAATAACAGTAAA
TCTTTTGCAGATATTCATCTATACAGAGAACAGATCAAGAGAGTGAAGGACTCGGATGATGTTCCCATGGTTCTTGTGGGCAACAAAT
GTGATTTGCCAACGAGGACAGTTGATACCAAACAAGCACAGGAGTTGGCACGAAGCTATGGGATTGAGTTTGTGGAGACTTCTGCAAA
GACCCGGCAGGGAGTGGAAGATGCCTTTTACACTTTGGTGCGGGAGATCCGGCAT

shark.NRAS

ATGACGGAATATAAACTGGTCGTGGTCGGAGCTGGTGGTGTGGGAAAAAGTGCCTTAACTATCCAGCTGATTCAGAACCACTTTGTGG
ATGAATATGATCCTACAATTGAGGATTCCTACAGGAAGCAAGTTGTAATTGATGGTGAAACATGTTTGTTAGACATTCTGGACACAGC
TGGTCAAGAGGAGTATAGTGCTATGAGAGATCAGTACATGAGAACTGGAGAGGGATTCCTCTGTGTGTTTGCCATAAATAATAGCAAA
TCCTTTGCAGATATTCACTTATACAGGGAACAGATAAAAAGAGTAAAAGATTCTGAAGATGTACCCATGGTCCTTGTAGGGAACAAAT
GTGATTTACCTGCCCGAACGGTGGACACAAAACAGGCACAGGAACTGGCTAGGAGTTATGGGATCCCTTTCATAGAAACCTCAGCCAA
AACGAGGCAGGGTGTTGAAGATGCATTTTACACACTGGTACGAGAGATCCGGCAG

lampreyHRAS

ATGACGGAGTACAAGTTGGTGGTGGTTGGCGCAGGAGGCGTGGGCAAGAGTGCCCTCACCATCCAGCTCATCCAGAACCACTTTGTGG
ACGAGTACGACCCCACCATCGAGGACTCGTACCGCAAGCAGGTGGTGATTGACAGCGAGACGTGTCTGCTGGACATCCTGGACACGGC
CGGGCAGGAGGAGTACAGCGCCATGCGCGACCAGTACATGCGCACAGGCGAGGGCTTCCTCTGCGTCTTCGCCATCAACAACTCAAAG
TCCTTCGAGGACATCCACCAGTACAGGGAGCAGATCAAGCGTGTCAAGGACTCGGACGACGTGCCCATGGTACTGGTGGGCAACAAGT
GTGACCTCCCAGCACGCACAGTCGACACCAAACAGGCGCAGGACCTGGCGCGCAGCTACGGGATCCCTTACGTTGAGACGTCCGCCAA
GACGCGACAGGGCGTGGAAGATGCGTTTTACACCTTGGTGCGTGAGATCCGGGAC

LampreyETR.KRAS

ATGACGGAGTACAAGCTGGTGGTGGTGGGCGCTGGAGGCGTTGGCAAGAGCGCCCTCACCATCCAGCTCATCCAGAACCACTTTGTGG
ACGAGTACGACCCCACCATCGAGGATTCGTACCGCAAGCAGGTGGTGATCGACGGGGAGACGTGCCTGCTGGACATCCTGGACACGGC
CGGGCAGGAGGAGTACAGCGCCATGCGAGACCAGTACATGCGCACGGGCGAGGGCTTCCTCTGCGTCTTCGCCATCAACAACTCCAAG
TCCTTCGAGGACATCCACCAGTACAGGGAACAGATCAAGCGCGTCAAGGACTCCGAGGACGTGCCGATGGTGCTGGTGGGCAACAAGT
GCGACCTGCCCACGCGGACCGTGGACACGAAGCAGGCGCAGGAGCTGGCGCGCAGCTACGGCATCCCCTTCATCGAGACCTCCGCCAA
GACCCGGCAGGGCGTGGACGACGCCTTCTACACGCTGGTGCGGGAGATCCGCAAG

hagfish.KRAS

ATGACGGAGTACAAGTTAGTGGTGGTGGGTGCTGGTGGCGTGGGCAAGAGCGCACTCACCATCCAGCTCATTCAGAACCACTTTGTAG
ATGAATATGATCCCACCATAGAGGATTCATACCGGAAGCAGGTGGTGATAGATGGCGAGACCTGTCTGCTGGATATCTTGGACACAGC
GGGTCAGGAGGAATACAGTGCAATGCGAGACCAGTACATGCGCACTGGGGAGGGCTTCCTCTGCGTGTTCGCCGTTAACAACTCTAAG
TCTTTTGACGACATTCACCTCTACAGGGAACAGATAAAGAGAGTGAAAGATTCGGAAGATGTCCCCATGGTTTTGGTTGGAAACAAAT
GTGACCTCCCGACCCGTACTGTTGACACCAAGCAAGCACTTGACCTTGCACGATCTTTTGGTATCCCTTGTATTGAGACCTCAGCCAA
GACTCGGCAGGGGGTGGACGATGCCTTTTATACGCTTGTCCGTGAAATCCGCAAG

hagfish.HRAS

ATGACGGAGTACAAGCTGGTGGTGGTGGGTGCGGGTGGTGTTGGCAAAAGTGCACTGACTATCCAGCTCATCCAGAACCACTTTGTTG
ATGAATACGACCCCACGATAGAGGACTCGTATAGGAAGCAGGTGGTGATAGACAGCGAAACATGCCTGTTGGATATCCTGGACACAGC
TGGTCAGGAAGAGTACAGTGCCATGCGTGATCAGTACATGCGCACTGGCGAAGGCTTCCTTTGTGTCTTCGCCATTAACAACACCAAG
TCTTTTGAGGACATTCACCAGTACAGGGAGCAAATAAAGCGTGTGAAGGACTCTGATGATGTACCAATGGTATTAGTTGGGAACAAGT
GTGATCTCCCAGCACGCACTGTGGATAGCAGGCAAGCGCAGGACCTGGCACGCTCGTACAACATCCCGTATGTGGAGACGTCTGCAAA
GACGCGTCAGGGTGTGGAGGACGCATTTTACACGTTGGTGCGAGAGATCCGGGAC

lancelet1.Bf

ATGACGGAGTACAAGTTGGTGGTGGTGGGTGACGGAGGCGTGGGGAAGAGTGCCCTCACCATACAGCTCATACAGAACCATTTTGTAC
AGGAGTACGACCCAACTATAGAGGACTCGTACAGAAAGCAGGTGGTGATCGATGGGGAGACGTGTTTGTTGGACATCTTAGACACAGC
AGGGCAAGAGGAGTACAGTGCCATGAGAGACCAGTACATGAGAACAGGAGAAGGATTTCTGTGCGTCTTTGCTATCAACAACGACAAA
TCCTTTGAGGACATTGCCATGTATAGGGAACAGATCAAGAGAGTAAAGGACTCAGACGAGGTTCCTATTGTCCTGGTGGGGAACAAGT

GTGACCTGCCCGCACGCACAGTGGAAAATAAACAGGCATGGACCCTGGCCAAGAGCTACGGAGTCCCCTACGTAGAGACATCTGCAAA
GACAAGAATGGGAGTGGACGACGCGTTCTACACACTGGTGCGAGAAATCAGGGCT

common_starfish

ATGACAGAATACAAATTGGTCGTCGTTGGAGCTGGTGGTGTAGGCAAGAGCGCCCTAACGATACAGCTCATTCAGAATCATTTTGTTG
ATGAATACGACCCTACCATTGAGGATTCATACCGTAAGCAAGTTGTTATAGATGGAGAGACGTGTTTACTGGACATTCTGGACTCCGC
AGGTCAAGAAGAGTATAGCGCAATGAGAGATCAATACATGAGGACTGGAGAAGGTTTTCTCATCGTGTTTGCCGTCAACAATCGTAAA
TCCTTTGAGGATTTCAATTTATATTGGTCCCAGATCTACCGAGTGAAGGATGCAGAAGAAGTCCCTATGGTCTTGGTAGGAAACAAAA
TTGACTTGTCAGACAGAACAGTTGAAGCAAGAGATGGTGCTGCAACAGCCAAGAGTTATGGCATTCCCTACATTGAGACATCAGCTAA
AACCAGACATGGAGTGGATGATGCATTTTACACGCTGGTTCGAGAAATTAGGAAA

sea_urchin

ATGACGGAGTATAAGTTAGTCGTAGTTGGAGCTGGAGGTGTAGGAAAAAGTGCATTGACGATACAACTTATCCAAAACCATTTTGTGG
ACGAGTATGACCCTACAATAGAGGATTCATATAGAAAACAGGTTGTAATAGATGGAGAGACGTGTTTACTGGATATCCTGGATACAGC
AGGTCAAGAAGAATACAGTGCCATGAGAGACCAATACATGAGAACGGGAGAAGGTTTCCTGTGTGTGTTTGCCATCAATAACGAGAAA
TCATTTGAGGATGTCAACCTGTACAGGGAGCAGATCAAACGAGTGAAGGATGCAGAAGAAGTTCCCATGGTCTTGGTTGGAAACAAAA
TTGACTTGCCGAACTGGGCTGTGCAAACGAGACAAGGACTGTCACTAGCTAAGAGTTATGGAATACCTTACATCGAAACATCAGCTAA
GACAAGACAAGGAGTAGACGATGCCTTCTACACACTCGTAAGGGAGATCAGGAAG

Drossophila.Ras85D

ATGACGGAATACAAACTGGTCGTCGTTGGAGCCGGAGGCGTGGGCAAGTCCGCGCTCACCATCCAGCTAATCCAGAACCATTTCGTGG
ACGAGTACGACCCCACAATCGAGGACTCTTACCGAAAGCAAGTGGTTATCGATGGAGAGACCTGCCTGCTGGACATCCTGGACACCGC
CGGCCAAGAGGAGTACTCGGCCATGCGGGATCAGTATATGCGGACTGGCGAGGGATTCCTGCTGGTCTTTGCCGTCAACAGTGCGAAG
TCCTTCGAGGATATCGGCACCTACCGTGAGCAGATCAAGCGCGTAAAGGATGCCGAAGAGGTGCCCATGGTGCTGGTGGGCAACAAAT
GTGATCTGGCCTCGTGGAACGTTAACAACGAGCAGGCAAGAGAGGTGGCCAAACAGTACGGCATTCCATACATTGAGACATCCGCCAA
GACGCGCATGGGCGTGGACGATGCATTTTACACACTGGTGCGCGAAATCCGCAAG

C.Elegans.let-60

ATGACGGAGTACAAGCTTGTGGTAGTTGGAGATGGAGGAGTTGGTAAATCAGCACTCACCATTCAACTCATCCAGAATCACTTTGTCG
AAGAATACGACCCGACCATAGAGGACAGCTACAGAAAGCAGGTTGTGATAGACGGTGAGACATGCCTCCTCGACATATTGGATACCGC
CGGACAAGAAGAATATTCGGCGATGCGTGATCAGTACATGAGGACAGGCGAAGGATTTCTGTTGGTTTTCGCCGTCAACGAGGCTAAA
TCTTTCGAGAATGTCGCTAACTACCGCGAGCAGATTCGGAGGGTAAAGGATTCAGATGATGTTCCTATGGTCTTGGTAGGGAATAAAT
GTGATTTGTCATCTCGATCAGTCGACTTCCGAACAGTCAGTGAGACAGCAAAGGGTTACGGTATTCCGAATGTCGACACATCTGCCAA
AACGCGTATGGGAGTTGATGAAGCATTTTACACACTTGTTAGAGAAATTCGCAAG

sea_anemone

ATGACGGAGTACAAGCTGGTAGTTGTTGGAGCTGGCGGCGTGGGGAAAAGTGCTTTAACAATACAACTGATACAGAACCATTTTGTGG
ATGAGTATGATCCAACTATTGAGGATTCATATAGAAAGCAAGTAGTAATAGATGGTGAAACATGTTTGCTGGATATATTGGACACAGC
AGGACAAGAAGAATACAGTGCTATGCGAGATCAGTACATGCGCACAGGGGAGGGCTTCCTCTGTGTTTTTGCTGTAAATAATAGTAAA
TCATTTGAAGATATTAACCAATATAGGGAGCAGATTAAACGAGTAAAAGATGCCGAGGAAGTCCCTATGGTTTTGGTAGGAAATAAAT
GTGACTTACCACAGCGAACAGTAAGCACATCTGATGCCCAGGAACTAGCAAAAAGCTACAATATCCCTTTTCAAGAGACCTCTGCAAA
AACTCGGCAGGGGGTGGATGACGCATTTTATACACTCGTCAGAGAAATCAGAAAA

soft_coral

ATGACTGAATATAAGCTCGTCGTTGTCGGAGCTGGCGGTGTGGGGAAAAGCGCTTTGACAATTCAGCTAATACAAAACCATTTTGTTG
ATGAATACGATCCCACAATAGAAGATTCATACAGAAAGCAAGTAGTTATAGATGGAGAAACTTGTTTATTGGATATCCTTGACACTGC
TGGACAGGAGGAATATAGTGCAATGAGAGATCAATATATGCGGACAGGGGAAGGGTTTCTATGCGTGTTCGCGATAAATAATGTAAAA
TCTTTCGACGACGTACATCAATACAGAGAACAGATAAAGAGAGTGAAAGATGCCGACGAAGTTCCTATGGTTTTGGTAGGCAATAAAT
GTGATTTACCGACACGAAACGTGAGCAGTGAAAGTGCAAGAGAGTTGGCATCAAGCTTCAGCATCCCATTCATAGAAACATCAGCAAA
AACGAGACAAGGAGTCGACGAAGCATTCTACACTTTAGTACGAGAAATACGAAAA

sponge.Aq

ATGACTGAGTATAAGCTAGTGGTAGTTGGAGCTGGTGGTGTAGGCAAGAGTGCATTGACTATACAGCTAATTCAGAATCATTTTGTTG
ATGAGTATGACCCCACGATAGAGGACTCCTATCGCAAGCAAGTGGTCATTGATGGAGACACTTGTCTACTGGACATCCTAGATACAGC
AGGACAGGAGGAATATAGTGCTATGAGAGACCAGTATATGAGAACAGGAGAGGGTTTCTTGTGTGTTTTTGCAGTCGATAATATGAAG
TCCTTTGAAGATGTGGAGTCCTACAGAGCACAGATACGCCGAGTAAAAGACTCAAATGACGTTCCTATAATCTTGGTAGGTAACAAGA
TTGATCTACCCAGACGAGAAGTTGATCAGAAGATAGCGCAAGGTTTTGTCAAGAACCACAACATGGCAGGATACATTGAGACTTCAGC
CAAAACAAGACAAGGAGTGGACGATGCTTTCTATTCTTTAGTACGAGAGATTAGACGC

salpingoeca

ATGACAGAGTACCGGCTCGTCGTTGTTGGAACTGGTGGCGTGGGAAAGTCTGCGCTTACCATCCAGCTCATCCAACAACACTTCGTCA
CCGAGTATGATCCAACCATCGAGGACTCGTACCGAAAACATGTGTCCATCGACGATGAGGCATGCCTCCTTGATATCCTTGACACAGC
CGGCCAGGAAGACTACAGTGCAATGCGGGATCAGTACATGCGGACGGGCGAAGGGTTTCTGTGCGTGTATTCGATTGATTCACAGCAG
TCGTTGGACGAGATACACTCCTTCCGTGAACAAATCCTCAGAGTCAAGGACCAAGATGAGGTTCCCATGATCCTTGTGGGCAACAAGT
GCGATCTTGAGGAGCACCGGGAAGTGTCGACAGAGGCCGGACAGGCCGTTGCAAAGTCGTACAGCATCCCCTTTATGGAGACATCCGC
AAAGAAGCGCATCAACGTGGAGGAGGCGTTTTACCAGCTCGTGCGTGAGATTCGCAAG

capsaspora

ATGACTGAGTACAAGCTCGTTGTTGTTGGCGGCGGCGGTGTCGGCAAGTCCGCCTTGACCATCCAGCTCATCCAAAACCACTTCGTGG
ACGAGTATGATCCCACCATTGAGGACTCGTACCGCAAGCAGGTCGTCATCGACGATGAGACGTGCCTACTCGACATTCTCGATACCGC
CGGCCAGGAGGAATACAGCGCCATGCGCGACCAATACATGCGAACTGGCGAGGGCTTCCTGTGCGTGTACGCCATCACTTCGCGACCA
TCGTTTGAGGAAATCCATCCTTTCAGGGAGCAAATTTTGCGAGTTAAGGATGCCGACGAAGTGCCCATGATTCTTGTTGCCAACAAGT
GCGATCTTGAGAGCGATCGTCAAGTGTCGCACACGGAAGGCCAGGCAACAGCCAAGCAATTTGGCATTCCCTTCATGGAGACTTCTGC
AAAGACCCGAACGCGTGTCGAGGACGCCTTCTACACTCTTGTTCGTGAAATTCGCCGC

Thecamonas

ATGACCGAGTACAAGCTTGTTGTTGTTGGTGGCGGTGGTGTCGGCAAGTCCGCCCTCACCATCCAGCTCATCCAGAACCACTTTGTTG
ATGAGTATGACCCCACCATTGAGGACTCGTACCGCAAGCAGGTCATCATCGACCAGGAGACGTGCCTGCTGGATATTCTTGATACCGC
CGGACAGGAGGAGTACTCGGCGATGCGTGACCAGTACATGCGGACCGGCGAGGGCTTCCTCTGCGTCTACGCCATCACCTCGCGCTCG
TCGTTTGAGGAGATCACCGCGTTCCGCGAGCAGATCCTCCGTGTCAAGGACGCCGACGCTGTCCCGATCATGATCTGCGGCAACAAGT
GCGATCTCGAGTCGGAGCGCCAGGTCTCTGCTGCCGAGGGCGAGGAGCTTGCCAAGTCGTTCACCGTCCCGTTCCTCGAGACCTCAGC
CAAGACCCGCGTCAACGTCGAGGAGGCGTTCTTCCAGCTCGTCCGTGAGATCCGTCGC

Dictyostelium

ATGACAGAATATAAATTAGTTATTGTAGGTGGTGGTGGTGTTGGTAAAAGTGCATTAACAATTCAATTAATTCAAAATCATTTTATTG
ATGAATATGATCCAACAATTGAAGATAGTTATCGTAAACAAGTTTCAATTGATGATGAAACTTGTTTATTAGATATTTTAGATACTGC
AGGTCAAGAGGAATATAGTGCAATGAGAGATCAATATATGAGAACTGGTCAAGGATTTTTATGTGTTTATTCAATTACATCAAGATCA
TCATATGATGAAATTGCATCATTTAGAGAACAAATTCTAAGAGTTAAAGACAAAGATAGAGTACCATTGATTTTGGTTGGTAATAAAG
CAGATTTGGATCATGAACGTCAAGTTAGTGTAAATGAAGGTCAAGAACTTGCAAAAGGATTCAATTGTCCTTTCATGGAGTCATCTGC
TAAAAGTAGAATTAATGTTGAAGAGGCATTTTACTCTTTAGTTCGTGAAATTAGAAAA

yeastSpRAS1

TTAAGAGAGTACAAATTGGTAGTTGTAGGAGATGGTGGTGTTGGTAAAAGTGCTTTGACAATTCAATTAATTCAATCGCATTTTGTTG
ACGAATACGATCCTACAATCGAAGATTCATATCGGAAAAAGTGTGAAATTGATGGTGAAGGTGCGCTTTTAGATGTATTGGACACGGC
CGGTCAAGAGGAATATTCCGCTATGCGTGAACAATATATGAGAACCGGAGAGGGTTTTTTATTAGTTTATAATATTACCTCTCGGTCC
TCTTTTGACGAAATTTCTACATTTTATCAACAAATCCTTAGAGTTAAGGATAAGGATACATTTCCAGTCGTTTTAGTGGCTAATAAAT
GCGACCTTGAAGCTGAACGTGTAGTTTCAAGAGCTGAAGGCGAACAACTTGCAAAAAGCATGCATTGTTTATATGTTGAAACTTCGGC
GAAGCTAAGGTTAAATGTAGAAGAAGCTTTTTATAGTTTGGTTCGCACGATCCGTCGC

yeastScRAS2

ATAAGAGAGTACAAGCTAGTCGTCGTTGGTGGTGGTGGTGTTGGTAAATCTGCTTTGACCATACAATTGACCCAATCGCACTTTGTAG
ATGAATACGATCCCACAATTGAGGATTCATACAGGAAGCAAGTGGTGATTGATGATGAAGTGTCTATATTGGACATTTTGGATACTGC
AGGGCAGGAAGAATACTCTGCTATGAGGGAACAATACATGCGCAACGGCGAAGGATTCCTATTGGTTTACTCTATAACGTCCAAGTCG
TCTCTTGATGAGCTTATGACTTACTATCAACAGATATTGAGAGTCAAAGATACCGACTATGTTCCAATTGTGGTTGTTGGTAACAAAT
CTGATTTAGAAAACGAAAAACAGGTCTCTTACCAGGACGGGTTGAACATGGCAAAGCAAATGAACGCTCCTTTCTTGGAGACATCTGC
TAAGCAAGCAATCAACGTGGAAGAGGCGTTTTACACTCTAGCACGTTTAGTTAGAGAC

yeastScRAS1

ATAAGAGAGTATAAGATAGTAGTTGTCGGTGGAGGTGGCGTTGGTAAATCTGCTTTAACAATTCAATTCATTCAATCATACTTTGTGG
ACGAATATGACCCTACTATCGAAGATTCTTACAGAAAACAAGTTGTCATCGATGACAAAGTATCCATTTTGGACATTCTAGATACTGC
TGGACAAGAAGAGTATTCTGCGATGAGAGAACAGTACATGAGGACTGGGGAAGGTTTCCTACTGGTCTATTCCGTCACCTCTAGAAAT
TCCTTTGATGAGTTACTGTCTTATTATCAGCAAATTCAAAGAGTAAAAGATTCTGACTACATTCCTGTAGTCGTGGTAGGTAACAAAT
TGGACCTTGAAAATGAAAGACAAGTCTCTTATGAAGACGGGTTACGCCTGGCCAAGCAGTTGAATGCACCCTTTCTAGAAACGTCTGC
GAAACAAGCCATCAACGTAGACGAGGCCTTTTATAGCCTTATTCGTTTGGTAAGGGAC

aspergillusRASA

CTTAGAGAGTACAAGCTAGTTGTTGTCGGTGGTGGTGGTGTCGGAAAGTCATGCTTAACAATCCAACTGATTCAGAGTCACTTCGTCG
ATGAATACGACCCAACAATTGAAGACTCCTACCGGAAGCAGTGTGTCATCGACGATGAGGTCGCTTTGTTGGATGTCTTAGATACTGC
CGGGCAGGAGGAGTACTCGGCGATGCGTGAACAATACATGCGGACTGGCGAAGGCTTCCTCCTGGTCTACTCGATTACGTCTCGGCAG
TCCTTTGAAGAGATTATGACTTTCCAACAACAAATCCTGCGTGTGAAAGACAAGGATTACTTCCCTATTATCGTGGTCGGTAACAAGT
GCGATTTGGAGAAGGAACGAGCCGTCTCTCAACAAGAGGGCGAGGCTCTGGCCAGGCAATTCGGTTGCAAGTTCATTGAAACGTCGGC
AAAATCCCGCATCAACGTTGAGAACGCTTTCTATGACCTCGTGCGCGAAATTCGTCGT

humanMRAS

CTCCCCACATACAAGCTGGTGGTGGTGGGGGATGGGGGTGTGGGCAAAAGTGCCCTCACCATCCAGTTTTTCCAGAAGATCTTTGTGC
CTGACTATGACCCCACCATTGAAGACTCCTACCTGAAACATACGGAGATTGACAATCAATGGGCCATCTTGGACGTTCTGGACACAGC
TGGGCAGGAGGAATTCAGCGCCATGCGGGAGCAATACATGCGCACGGGGGATGGCTTCCTCATCGTCTACTCCGTCACTGACAAGGCC
AGCTTTGAGCACGTGGACCGCTTCCACCAGCTTATCCTGCGCGTCAAAGACAGGGAGTCATTCCCGATGATCCTCGTGGCCAACAAGG
TCGATTTGATGCACTTGAGGAAGATCACCAGGGAGCAAGGAAAAGAAATGGCGACCAAACACAATATTCCGTACATAGAAACCAGTGC
CAAGGACCCACCTCTCAATGTCGACAAAGCCTTCCATGACCTCGTTAGAGTAATTAGGCAA

SharkMRAS

CTCCCCACGTACAAGCTGGTGGTGGTGGGCGACGGAGGTGTGGGGAAAAGTGCCCTGACCATCCAGTTCTTCCAGAAGATCTTTGTGC
CGGATTACGACCCGACCATTGAAGACTCTTACTTGAAGCACACGGAAATCGATGGACAGTGGGCCATTTTGGATGTGCTGGACACGGC
GGGTCAGGAGGAGTTCAGTGCTATGAGGGAGCAGTACATGAGGACTGGCGATGGCTTCCTCATCGTCTTTTCGGTCACAGACAAAGCC
AGCTTCGAGCACGTCGACAGGTTTCACCAGTTGATCCTCCGAGTCAAAGACAGGGAGTCTTTTCCGATGATCTTGGTGGCCAACAAGG
TTGACCTGGTCCACCTGCGGAAGGTCACCAGCGAACAGGGAAGAGAGATGGCGGCGAAGTACAATATACCTTACATAGAAACGAGTGC
CAAGGATCCACCGCTGAATGTTGACAAAGCTTTCCACGACCTTGTCCGTGTCATAAGGCAA

human.TC21

CAGGAGAAGTACCGGCTCGTGGTGGTCGGCGGGGGCGGCGTGGGCAAGTCGGCGCTCACCATCCAGTTCATCCAGTCCTATTTTGTAA
CGGATTATGATCCAACCATTGAAGATTCTTACACAAAGCAGTGTGTGATAGATGACAGAGCAGCCCGGCTAGATATTTTGGATACAGC
AGGACAAGAAGAGTTTGGAGCCATGAGAGAACAGTATATGAGGACTGGCGAAGGCTTCCTGTTGGTCTTTTCAGTCACAGATAGAGGC
AGTTTTGAAGAAATCTATAAGTTTCAAAGACAGATTCTCAGAGTAAAGGATCGTGATGAGTTCCCAATGATTTTAATTGGTAATAAAG
CAGATCTGGATCATCAAAGACAGGTAACACAGGAAGAAGGACAACAGTTAGCACGGCAGCTTAAGGTAACATACATGGAGGCATCAGC
AAAGATTAGGATGAATGTAGATCAAGCTTTCCATGAACTTGTCCGGGTTATCAGGAAA

sharkTC21

CAGGAGAAGTTCAAGCTGGTGGTGGTGGGCGGCGGCGGAGTGGGCAAGAGCGCCCTCACCATCCAGTTCATCCAGTCTTACTTTGTTA
CGGATTATGATCCAACAATTGAAGATTCCTACACCAAGCAATGCGTTATTGATGACCGAGCTGCAAGGTTGGACATTTTGGACACAGC
GGGACAGGAAGAGTTTGGAGCCATGCGAGAACAATACATGAGGACAGGGGAGGGCTTCCTGCTGGTCTTCTCAGTAACCGATAGAGGA
AGTTTTGAAGAGATTTACAAGTTCCAAAGACAGATCCTTCGGGTAAAAGATCGTGATGAATTCCCTATGATCCTAGTAGGAAACAAAG
CTGACCTGGAGCATCAGAGGCAGGTGACGTTAGAGGAAGGACAACAGCTGGCCCGTCAGCTCAAAGTCACGTACATGGAGGCTTCAGC
CAAAATCCGATTGAATGTCGACCAAGCTTTTCACCAACTGGTCCGAGTGATAAGGAGA

humanRRAS

AGCGAGACACACAAGCTGGTGGTCGTGGGCGGCGGCGGCGTGGGCAAGAGCGCGCTGACCATCCAGTTCATCCAGTCCTACTTCGTGT
CTGACTACGACCCCACTATTGAGGACTCCTACACGAAGATCTGCAGTGTGGATGGCATCCCAGCCCGGCTGGACATCCTGGACACCGC
GGGCCAGGAAGAGTTCGGGGCCATGAGAGAGCAGTACATGCGTGCTGGCCACGGCTTCCTGCTGGTGTTCGCCATTAACGACCGGCAG
AGTTTCAACGAGGTGGGCAAGCTCTTCACGCAGATTCTGCGGGTCAAGGACCGCGACGACTTCCCCGTTGTGTTGGTCGGGAACAAGG
CAGATCTGGAGTCACAGCGCCAGGTCCCCCGATCAGAAGCCTCTGCCTTCGGCGCCTCCCACCACGTGGCCTACTTTGAGGCCTCGGC
CAAACTGCGTCTCAACGTGGACGAGGCTTTTGAGCAGCTGGTGCGGGCTGTCCGGAAA

HVR DNA SEQUENCES

human.KRAS4B

GGTGTTGATGATGCCTTCTATACATTAGTTCGAGAAATTCGAAAACATAAAGAAAAGATGAGCAAAGATGGTAAAAAGAAGAAAAAGA
AGTCAAAGACAAAGTGTGTAATTATG

chicken.KRAS4B

GGTGTTGATGATGCCTTCTATACATTAGTTCGAGAAATCAGAAAACACAAAGAGAAGATGAGCAAAGATGGTAAAAAGAAGAAAAAGA
AGACAAAGACAAAGTGTATAATTATG

reedfish.KRAS4B

GGAGTGGATGATGCCTTTTATACATTAGTAAGAGAAATTCGAAAACACAAAGAGAAGATGAGCAAAGATGGGAAGAAGAAAAAGAAAA
AATCCAAGCCAAGATGTATACTTATG

shark.KRAS4B

GGCGTTGACGACGCTTTCTACACACTAGTCCGCGAAATCCGAAAGCACAAAGAGAAAATGAGTAAAGACGGCAAAAAGAAGAAAAATA
AAACCAAGAAAAAGTGTTCGATTATG

reedfish.KRAS4A

AGAGTGGAGGATGCCTTTTATACTCTGGTACGGGAGATACGTCAGTACCGGCTGAAAAAGTTCAGCAAGGAAGAAAAGACTCCACGCT
GTGTGAAGCTAAAAAAGTGTGTTGTTATG

chicken.KRAS4A

AGAGTGGAGGATGCTTTTTATACATTGGTGCGAGAGATTCGACAGTACAGAGTGAAAAAAATCAGCAAAGAAGAAAAGACTCCAGGGT
GCATGAAAATTAAAAAATGCCTTGTAATG

human.KRAS4A

AGAGTGGAGGATGCTTTTTATACATTGGTGAGAGAGATCCGACAATACAGATTGAAAAAAATCAGCAAAGAAGAAAAGACTCCTGGCT
GTGTGAAAATTAAAAAATGCATTATAATG

shark.KRAS4A

AGAGTGGAGGATGCATTTTATACACTGGTACGTGAGATTCGCCAATACAGGTTGAGAAAGCTCAACACTGAAGAAAAGACAACCAGAT
GCGTGGTGTTTAAATGTGTTGTGATG

human.NRAS

GGTGTTGAAGATGCTTTTTACACACTGGTAAGAGAAATACGCCAGTACCGAATGAAAAAACTCAACAGCAGTGATGATGGGACTCAGG
GTTGTATGGGATTGCCATGTGTGGTGATG

chicken.NRAS

GGTGTGGAAGATGCGTTTTACACACTGGTGAGGGAGATTCGGCAGTACCGGATGAAAAAGCTCAACAGCAACGAAGATGGGAATCAGG
GCTGTATGGGGTTGTCCTGCATTGTGATG

reedfish.NRAS

GGAGTGGAAGATGCCTTTTACACTTTGGTGCGGGAGATCCGGCATTACCGAATGAAGAAGCTCAACAGCAGTGAAGAGAGGAATCAGG
GATGCCTGGGAGTTTCCTGCACAGTTATG

shark.NRAS

GGTGTTGAAGATGCATTTTACACACTGGTACGAGAGATCCGGCAGTACAGAACGATAAAACTCAACAGTCAAGATGATAGAAACCAAG
GCTGCTTGGGACTAAAATGCATAATAATG

reedfish.KRASL

GGGGTGGAAGATGCTTTTTACACTCTTGTGCGGGAAATCCGAAAGCACAAAGAGAAAGTGAGCAGAAATGGGAAAAAAAAGACCTCAA
AGCGCAGGTGCACAATCTTA

shark.KRASL

GGCGTGGAAGATGCCTTCTACACACTCGTGCGTGAGATTCGCAAGTACAAGGAGAAGATCAGCAAAAACGGCAAAAAGAAGAAAAAGC
CCTCCAAGAAAAAGTGCATCATTCTT

reedfish.HRAS

GGAGTAGAGGATGCCTTCTACACTTTGGTGCGGGAAATCCGGCAGCATAAACTCCGAAAGCTGAACCCTCCAGATGAAAGCGGACAGG
ACTGCATCAGCTGTAAATGTGTCATTTCG

human.HRAS

GGAGTGGAGGATGCCTTCTACACGTTGGTGCGTGAGATCCGGCAGCACAAGCTGCGGAAGCTGAACCCTCCTGATGAGAGTGGCCCCG
GCTGCATGAGCTGCAAGTGTGTGCTCTCC

chicken.HRAS

GGCGTCGAAGATGCCTTCTATACCTTAGTGCGGGAGATCCGTCAGCATAAACTGCGCAAGCTGAACCCACCAGATGAGAG TGGCCCTG
GCTGCATGAACTGTAAATGCGTGATATCG

shark.HRAS

GGAGTGGAGGATGCCTTTTATACCTTGGTTCGTGAGATCAGCCAACACAAAGTGAGGAAGTCGAACCCTCCCGACGACAG CGGCCGAG
ACTGTAGCAACTGCAAGTGTGTAATATTG

lamprey.HRAS

GGCGTGGAAGATGCGTTTTACACCTTGGTGCGTGAGATCCGGGACCACAAACTGCGCAAGATGAACAACGCCAACAACAA GGACGAGT
GCAGCGTGTGCAGCTGCGTGCTCTCC

lancelet1.Bf

GGAGTGGACGACGCGTTCTACACACTGGTGCGAGAAATCAGGGCTTACAAGGAGAGACAAGGCAAAGCTGGCAAGAAGAA GAAGAAGA
ATTTCAAGAGCCGATGCACCATCTTG

hagfish.KRAS

GGGGTGGACGATGCCTTTTATACGCTTGTCCGTGAAATCCGCAAGTACAAGGAACGCGCAAGCAAGGATGGGAAAAAGAA AAAGAAGA
GATCTCGAAAAATGTGCTTGGTCATG

lamprey.KRAS

GGCGTGGACGATGCCTTCTACACGCTGGTGCGCGAGATCCGCAAGTACAAGGACCGCATGAGCAAAGACGGGCGCAAGAA GAAAAAGA
AATCGAAGAGGAAGTGCCTCCTCATG

hagfish.HRAS

GGTGTGGAGGACGCATTTTACACGTTGGTGCGAGAGATCCGGGACCACAAAGCCCGAAAGCTGCACAACATGGAGGACAA TGGTGGTG
GCTGTAATATGTGCAGCTGTGTAATTTCT
