# Supplementary figures and images for: Origin and Evolution of RAS Oncoprotein Membrane Targeting
Source: Res Sq. 2023 Jan 20:rs.3.rs-2485219. Preprint. [Version 1] doi: 10.21203/rs.3.rs-2485219/v1 (PMC9882654; doi:10.21203/rs.3.rs-2485219/v1)

Effector lobe 1-86

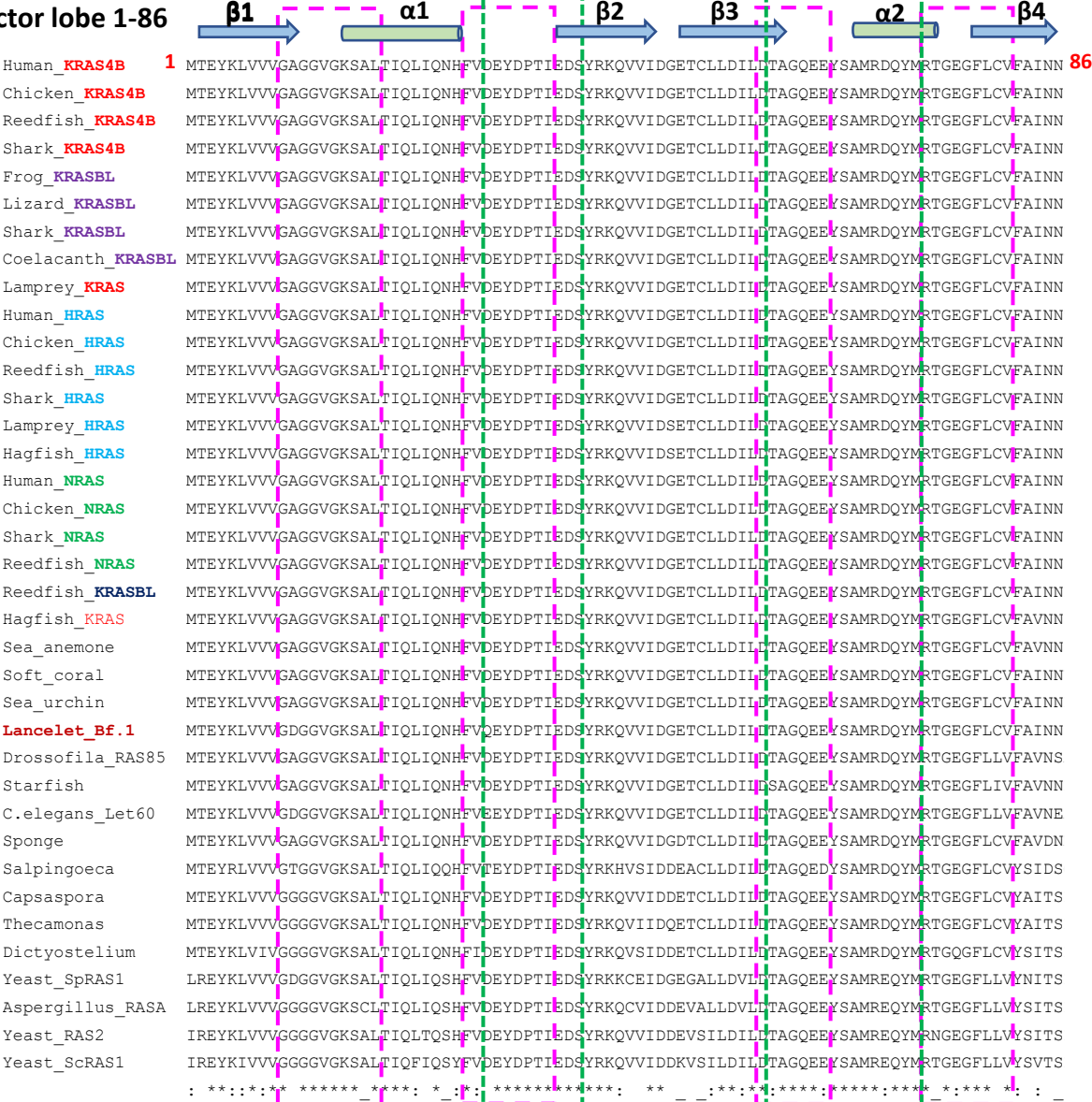

Alosteric lobe 87-166

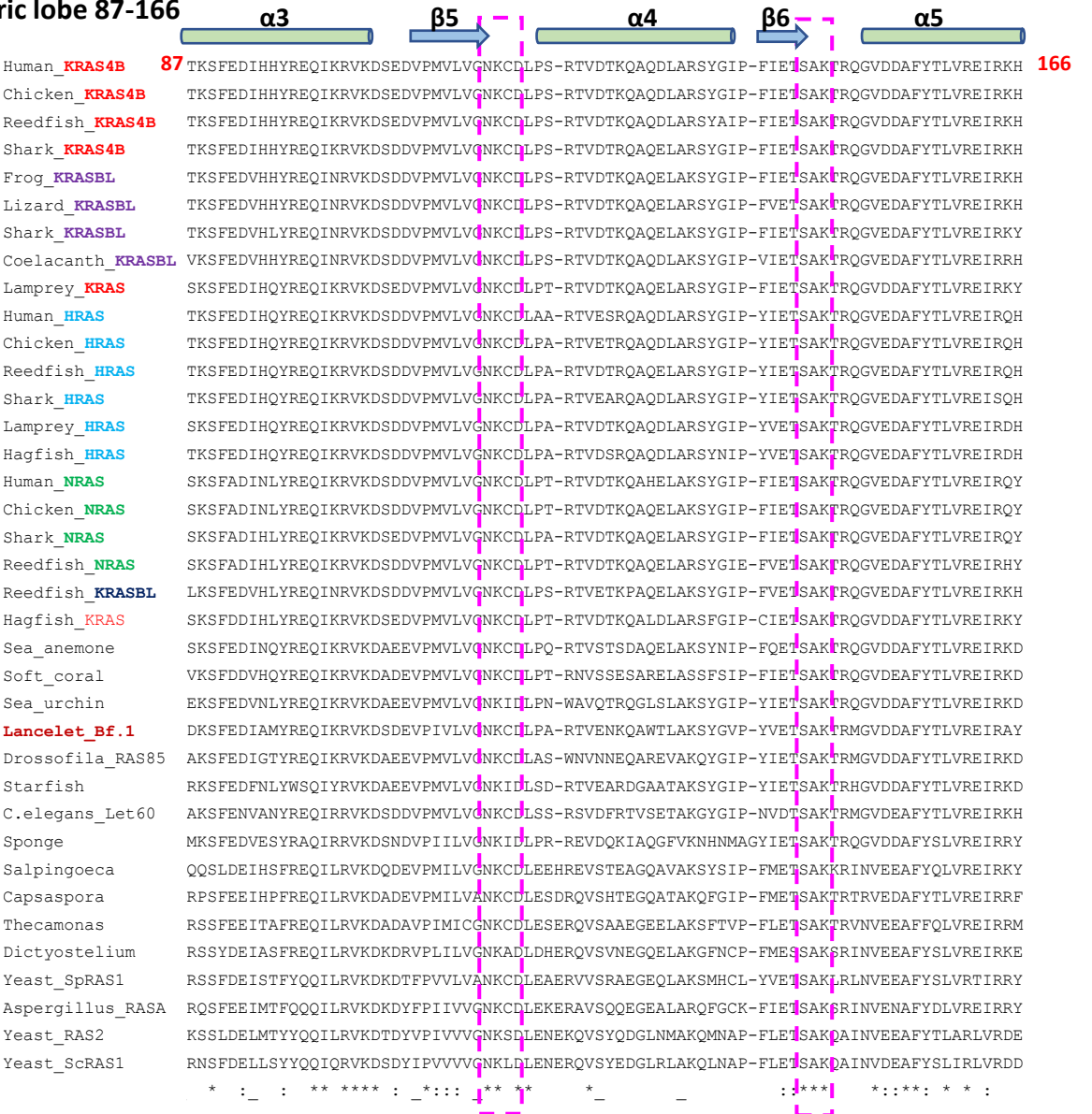

Suppl. Fig. 2

Supplement: Suppl. Fig 2 — Suppl. Fig. 2. Eukaryotic G-domain sequences are highly similar. The alignment of the full-length G-domain oncoprotein sequences from amoebas to humans was generated by the MAFFT server with default parameters. Upper panel effector lobe (residues 1-86) and lower panel allosteric lobe (residues 87-166). Switch-regions I and II (green boxes) and GTP/GDP binding elements (dashed pink boxes). Arrows and rectangles above the sequences α-helices and β-sheets, respectively. Amino acid identity: identical (*); strongly similar (:); weakly similar (.). [file Suppl.Fig.2_10.1.2023.pdf]

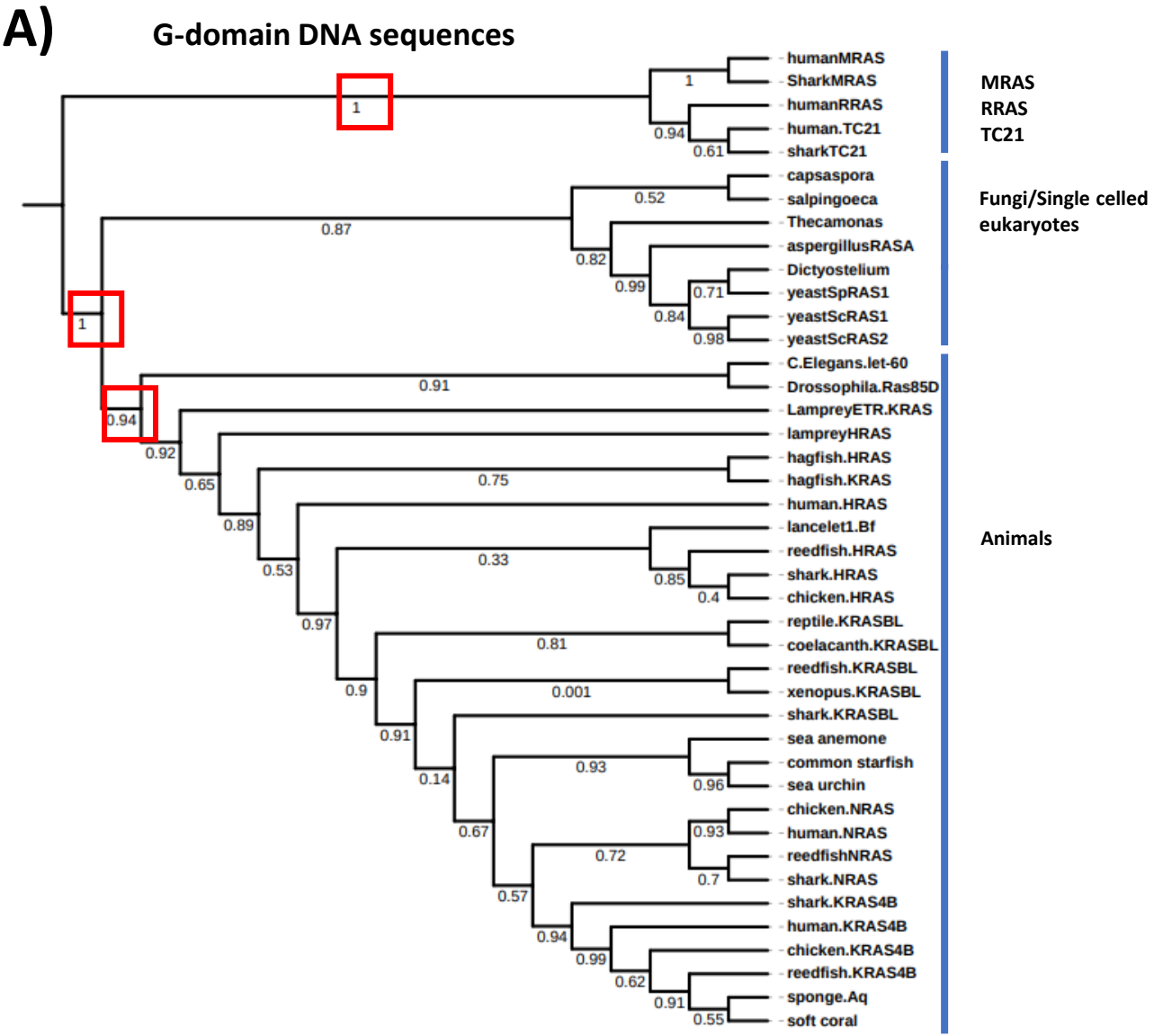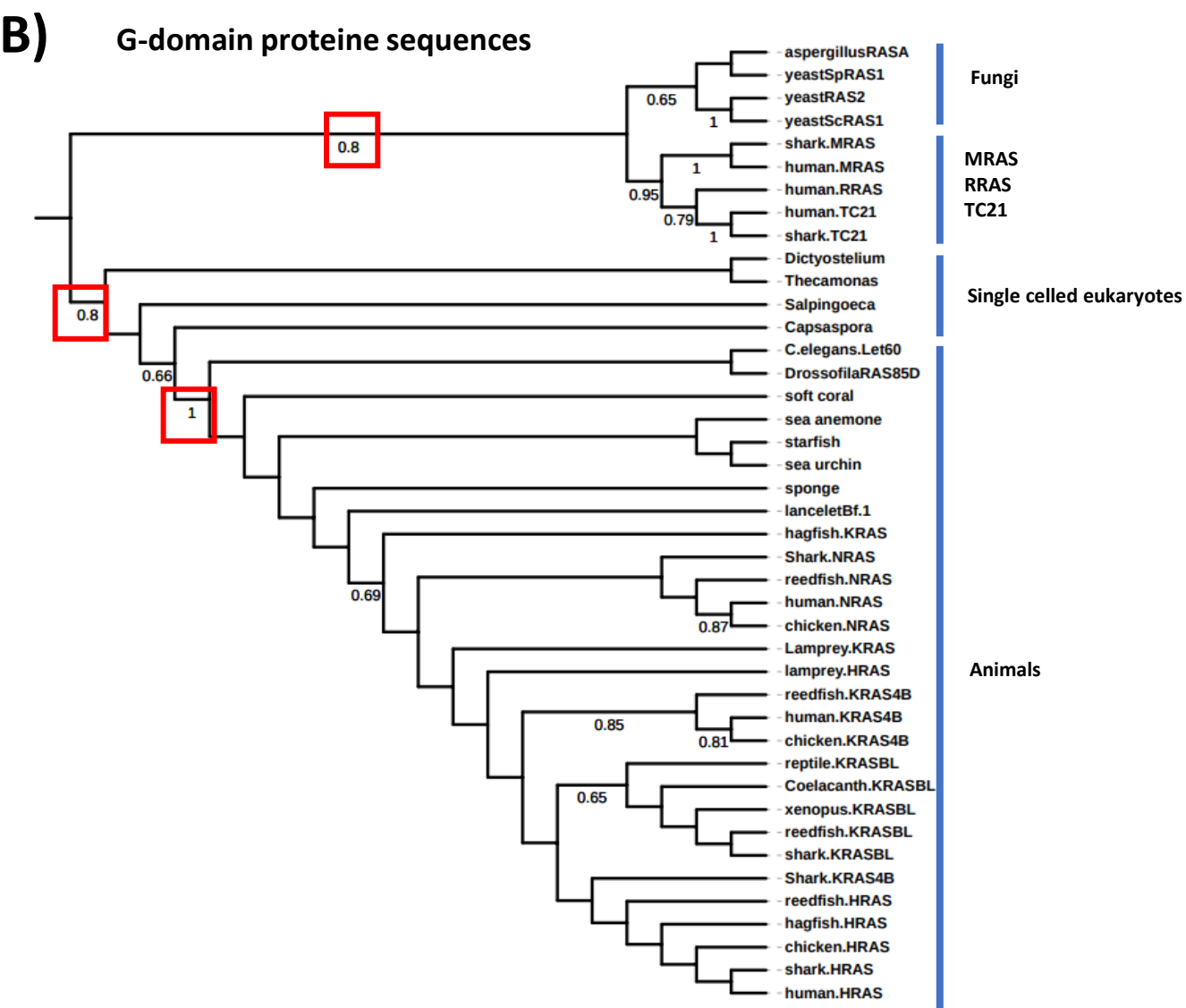

Suppl. Fig. 4

Supplement: Suppl. Fig 4 — Suppl. Fig. 4. Evolutionary relationships between eukaryotic G-domain sequences. (A) DNA sequences. (B) Protein sequences. Evolutionary relationships between DNA and protein sequences corresponding exclusively to G-domains, where analyzed using the tools on the Phylogeny.fr web site [63], MUSCLE for alignment, Gblocks for curation, PhyML for DNA tree building, and BioNJ with 1000 bootstrap replicates and the JTT substitution model for protein sequences [57, 63, 65]. The resulting outputs in the Newick files format were visualized using the Interactive Tree of Life platform 52. Bootstrap values are indicated below tree branches with value at group branch points boxed in red. G-domain sequences from RRAS, MRAS and TC21 which are the closest members to classical oncoproteins in the RAS family, where used as outgroup [12]. [file Suppl.Fig.4_10.1.2023.pdf]

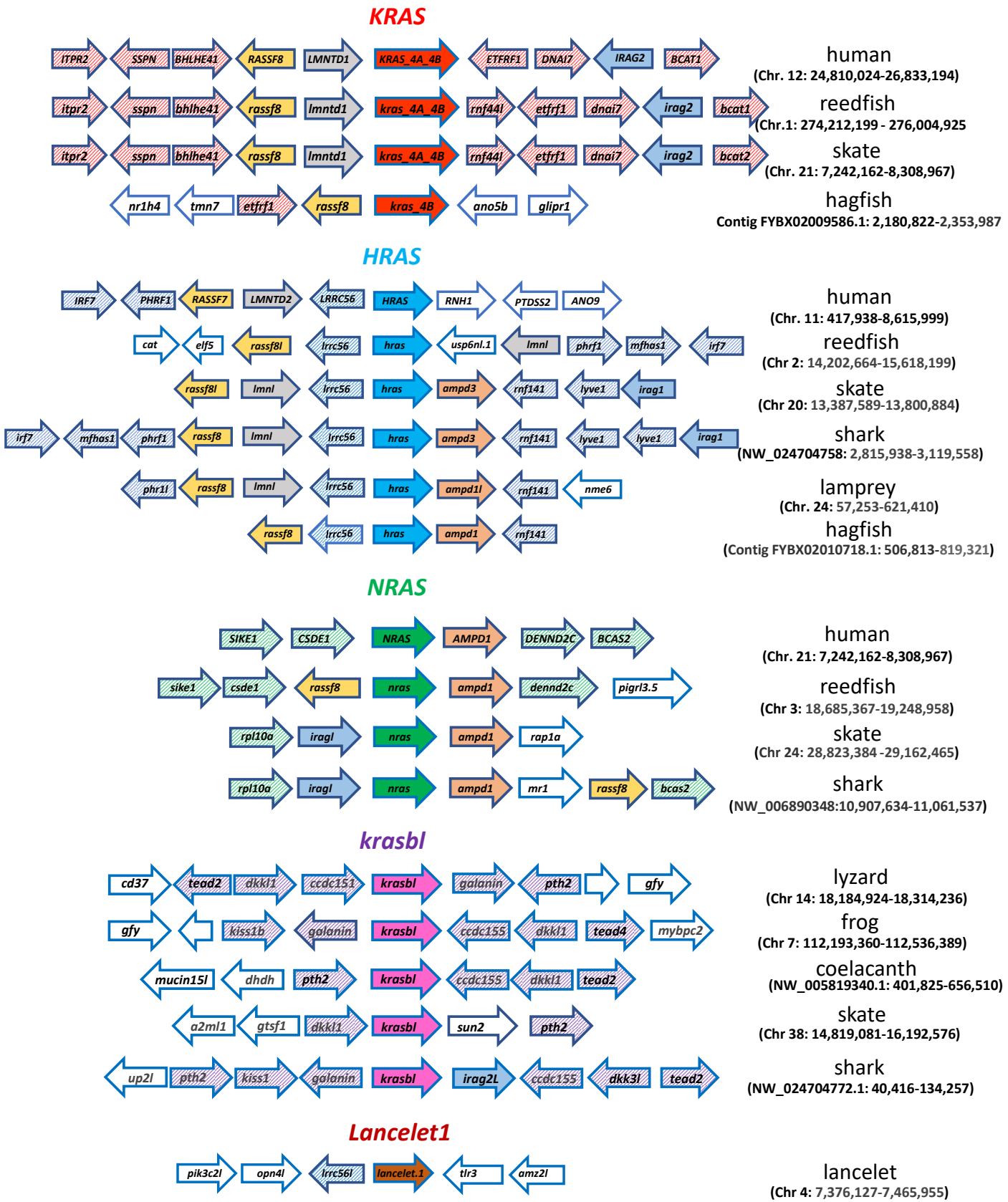

Suppl. Fig. 2

Supplement: Suppl. Fig 7 — Suppl. Fig. 7. Conservation of oncoproteins genomic architecture (synteny) from humans to lancelets. The figure depicts Ras oncogene genomic regions from cephalochordates to mammals. Genes are depicted as arrows pointing to the direction of transcription. Evolutionarily conserved solitary genes (hashed), duplicated genes (solid), and non-conserved (outline) are shown. Genomic coordinates are indicated below each species name. GenBank assembly accession numbers are human, GCF_000001405.39; lizard, GCF_009819535.1; frog, GCF_000004195.4; coelacanth, GCF_000225785.1; reedfish, GCF_900747795.1; skate, GCF_010909765.1; elephant shark, GCF_018977255.1; hagfish, GCA_900186335.2; lamprey, GCF_010993605.1; and lancelet, GCF_000003815.2. [file Suppl.Fig.7_10.1.2023.pdf]
