# Supplementary material for: Origin and Evolution of RAS Oncoprotein Membrane Targeting
Source: Res Sq. 2023 Jan 20:rs.3.rs-2485219. Preprint. [Version 1] doi: 10.21203/rs.3.rs-2485219/v1 (PMC9882654; doi:10.21203/rs.3.rs-2485219/v1)
Supplement: Suppl. Fig 3 — Suppl. Fig. 3. Eukaryotic G-domains pairwise amino acids identity. Heat map representing amino acids Blastp pairwise identities in comparison with human oncoproteins. Numerical values are depicted inside the cells, which are colored from green (low similarity) to red (high similarity). [file Suppl.Fig.3_10.1.2023.pdf]

			human		
			KRAS	HRAS	NRAS
Vertebrates	human	KRAS	100		
		HRAS	93,98	100	
		NRAS	92,77	91,57	100
	chicken	KRAS	100	93,89	
		HRAS	95,18	98,8	
		NRAS	93,37	92,17	99,4
	bony fish	KRAS	99,4	93,37	
		HRAS	95,18	97,59	
		NRAS	93,37	91,57	96,39
		KRASBL	93,37	91,57	92,77
	shark	KRAS	98,19	94,58	
		HRAS	93,98	98,19	
		NRAS	95,18	93,37	96,99
		KRASBL	95,18	92,17	95,18
	lamprey	KRAS	96,99	92,77	
	lamprey	HRAS	94,58	95,18	
	hagfish	KRAS	94,58	90,36	
	hagfish	HRAS	93,37	96,39	
Non-vertebrates		lancelet1_Bf	87,95	88,55	
		common starfish	84,24	84,24	
		sea urchin	89,7	87,88	
		C. elegans	83,13	81,93	
		DrossophilaRAS85D	87,27	86,06	
		sea anemoneNv	91,52	88,48	
		soft coral	88,48	87,88	
		sponge	81,44	79,64	
Single celled eukaryotes		Salpingoeca	73,05	72,46	
		Capsaspora	79,52	79,52	
		Thecamonas	74,7	74,7	
		Dyctiostelium	70,66	71,98	
Fungi		yeastSpRAS2	63,8	64,42	
		yeastScRAS1	63,03	62,42	
		aspergillusRASA	67,07	67,66	
		yeastSpRAS1	64,67	67,66	
MRAS , RRAS and RRAS2/TC21	human	MRAS	56,29	56,29	
	shark	MRAS	58,68	59,28	
	human	TC21	60,98	60,98	
	shark	TC21	60,98	61,59	
	human	RRAS	59,15	58,54	

Suppl. Fig. 3
