# Supplementary material for: Origin and Evolution of RAS Oncoprotein Membrane Targeting
Source: Res Sq. 2023 Jan 20:rs.3.rs-2485219. Preprint. [Version 1] doi: 10.21203/rs.3.rs-2485219/v1 (PMC9882654; doi:10.21203/rs.3.rs-2485219/v1)
Supplement: Suppl. Fig 5 — Suppl. Fig. 5. Oncoprotein specific signature conservation in eukaryotes. Protein Blast searches in NCBI protein eukaryotic databases were performed in Blastp with default parameters using as a bait the oncoprotein specific signature (EYDPTIEDSYRKxxxxxxxxxxxxxxxxAGQEEYSAMRDQYM) corresponding to the effector domain. Single non identical amino acids in one choanoflagellate species (S. rosseta) and in all Fungi are indicated with black rectangles. [file Suppl.Fig.5_10.1.2023.pdf]

Specific signature
EYDPTIEDSYRKxxxxxxxxxxxxxxxxAGQEEYSAMRDQYM

VERTEBRATA (Not shown)

CEPHALOCHORDATA

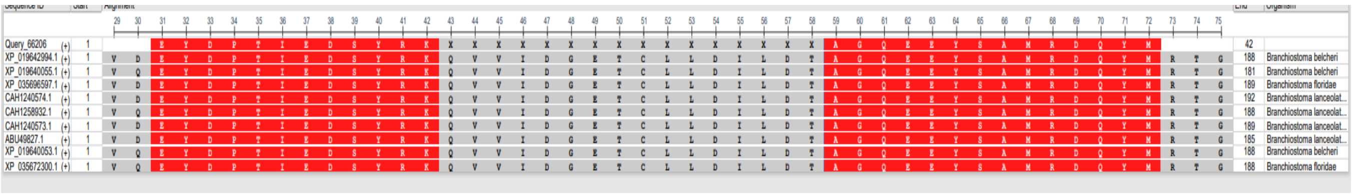

ECHINODERMATA

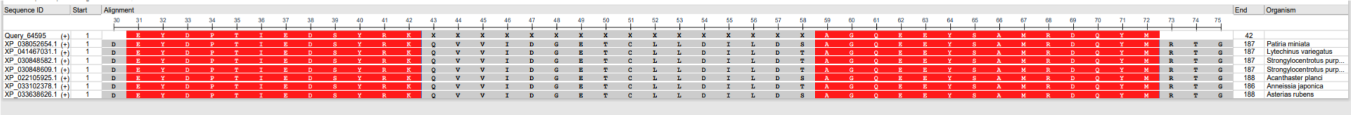

PROTOSTOMES

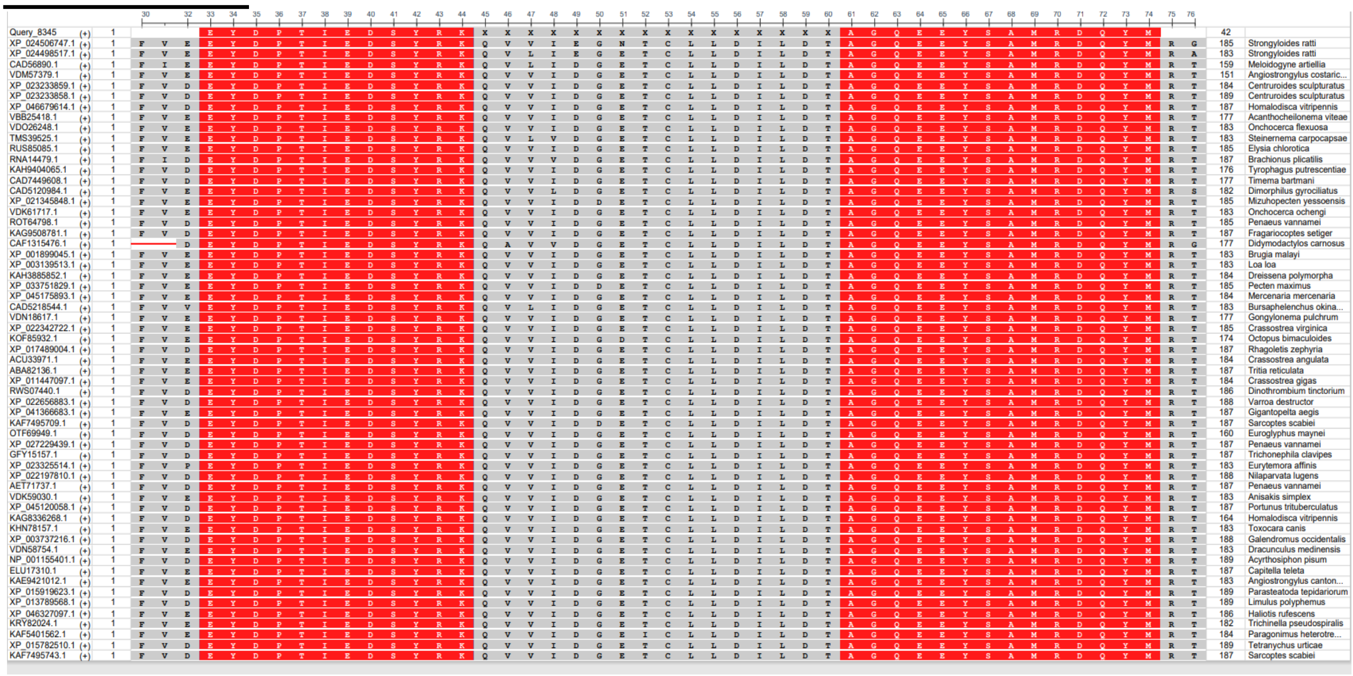

CNIDARIANS

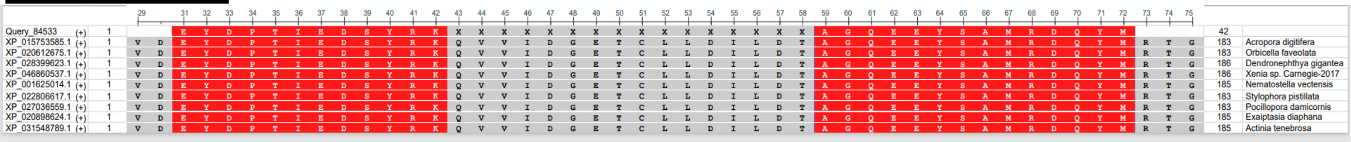

PORIFERANS

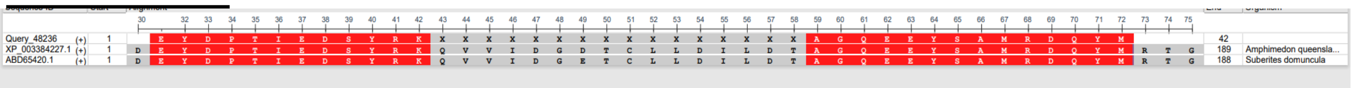

CHOANOFLAGELLATA

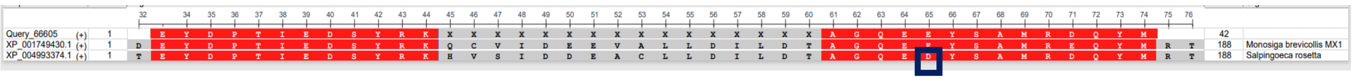

APUSOZOA

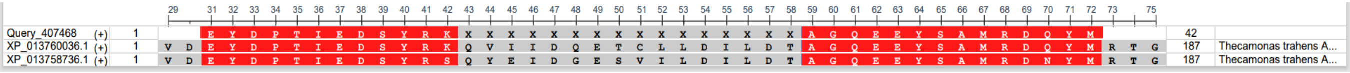

AMOEBOZOA

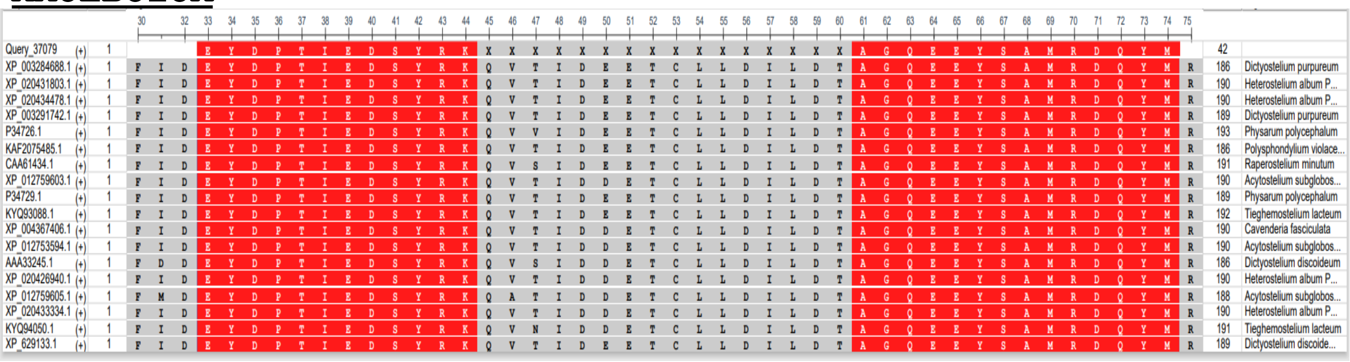

Fungi EYDPTIEDSYRKxxxxxxxxxxxxxxxTAGQEEYSAMREQYM

ASCOMYCOTA

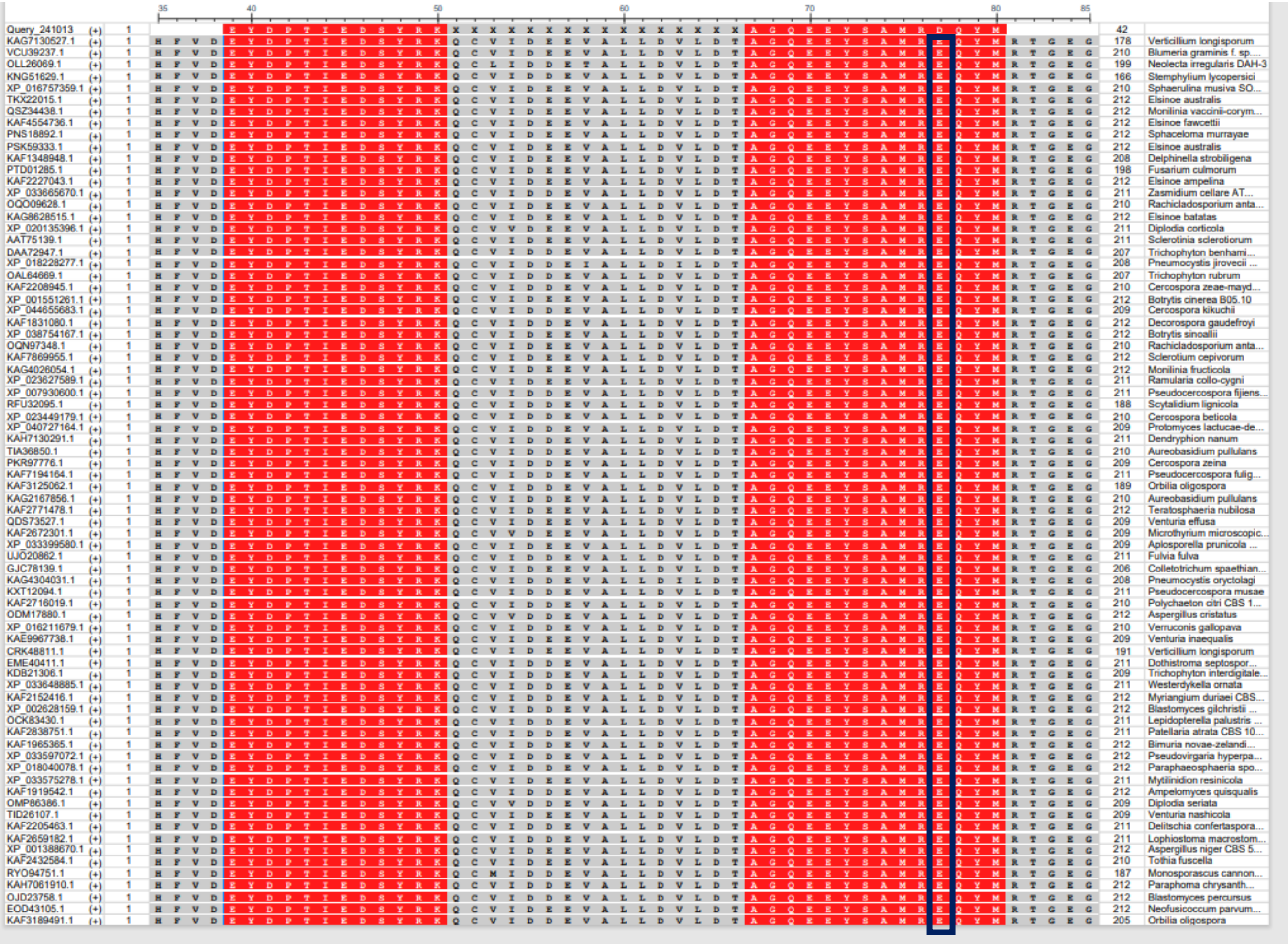

BASIDIOMYCOTA

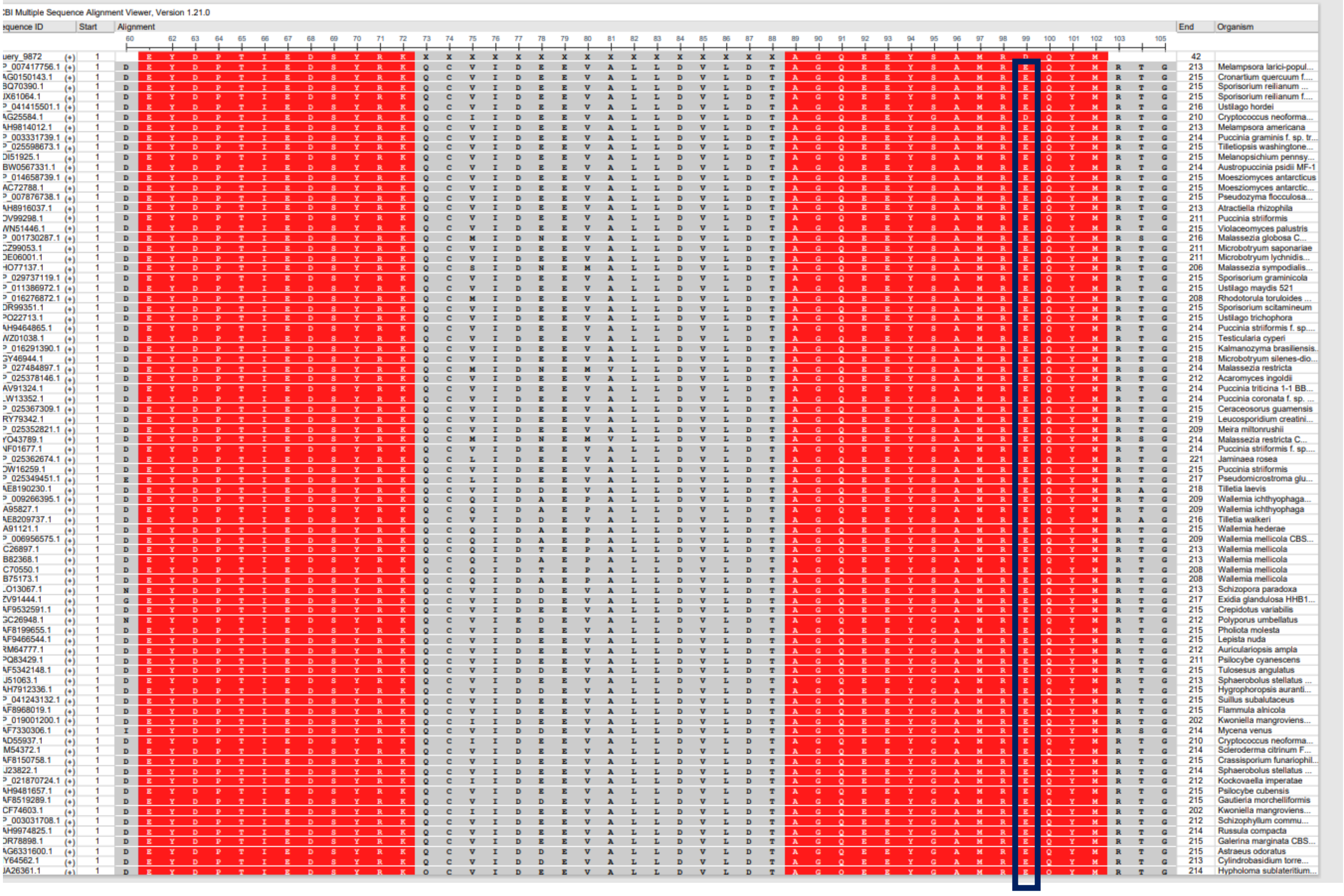

NEOCALLIMASTIGOMYCOTA

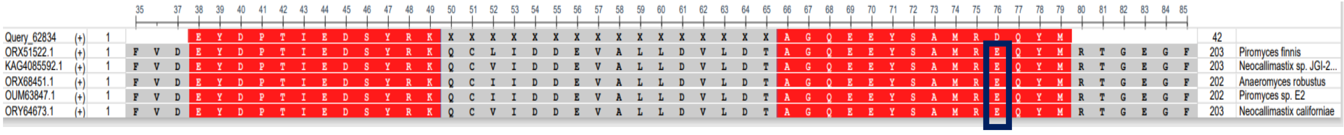

ZOOPAGOMYCOTA

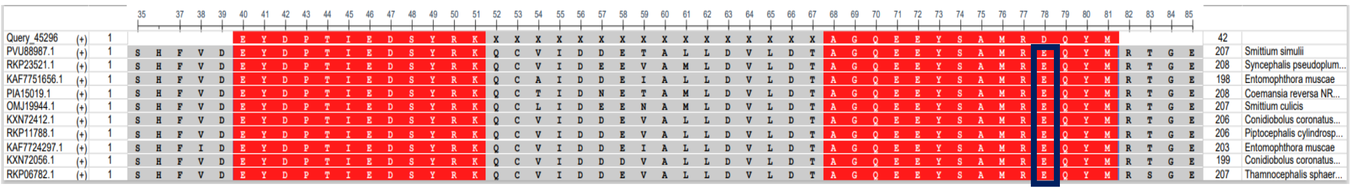

BLASTOCLADIOMYCOTA

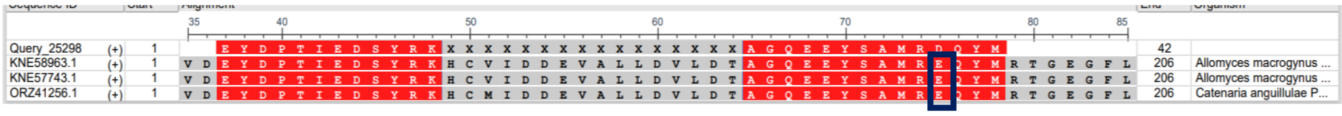

GLOMEROMYCOTA

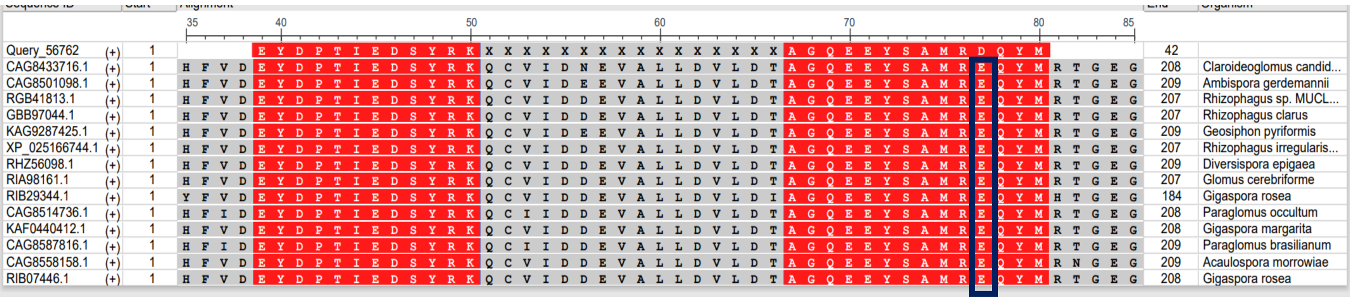

MUCOROMYCOTA

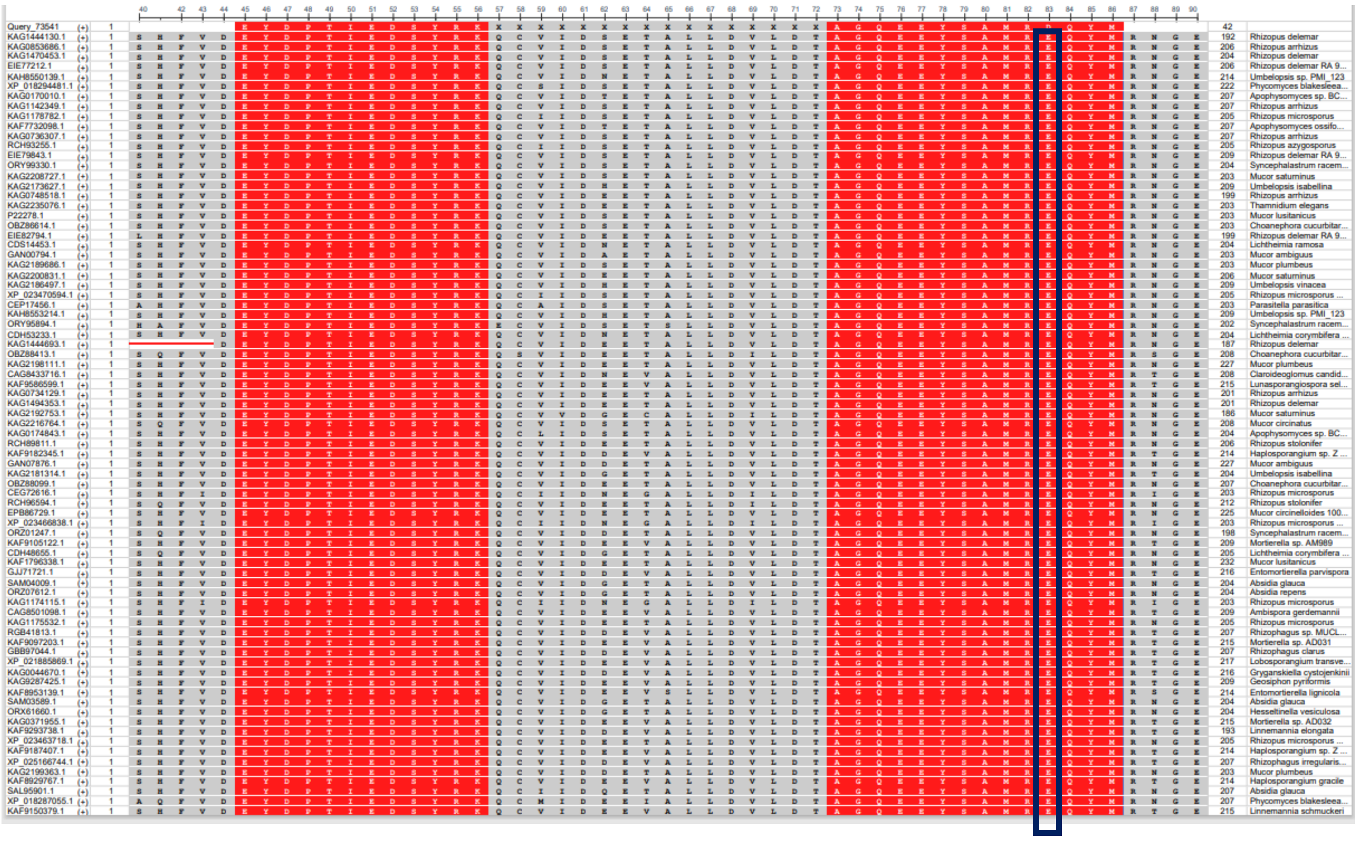

ZYGOMYCOTA

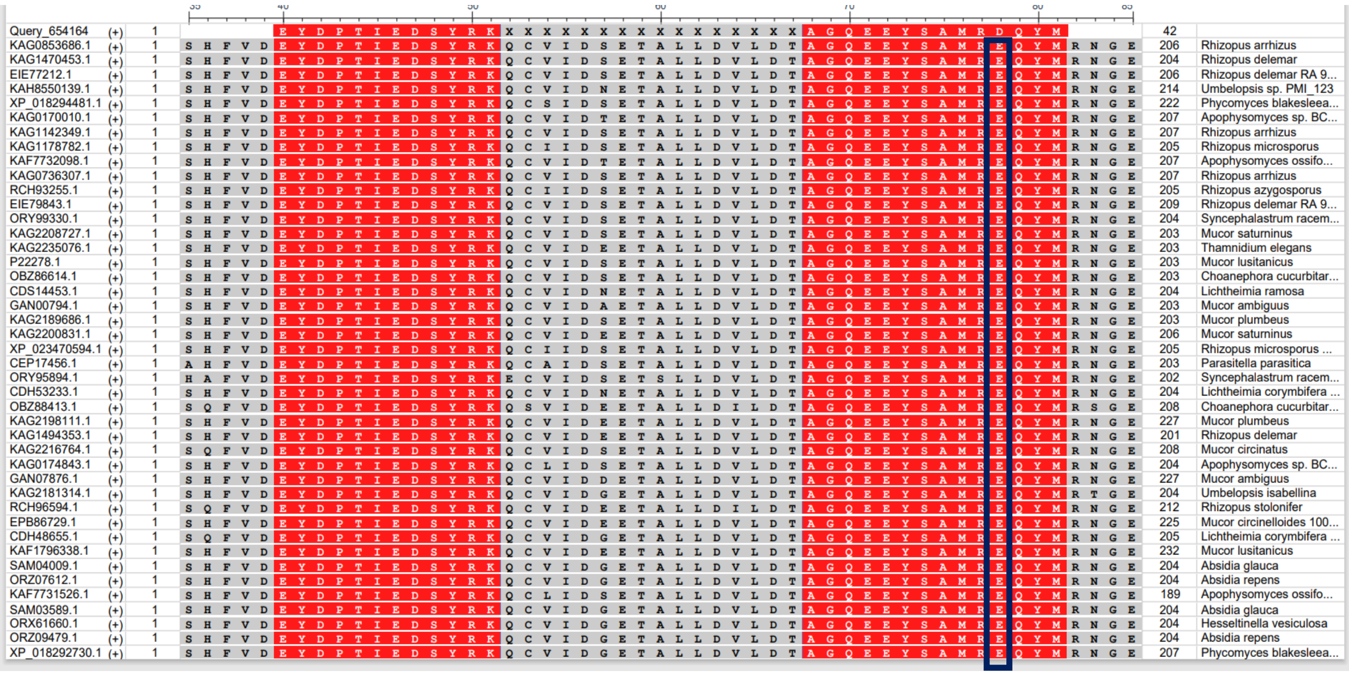

CHYTRIDIOMYCOTA

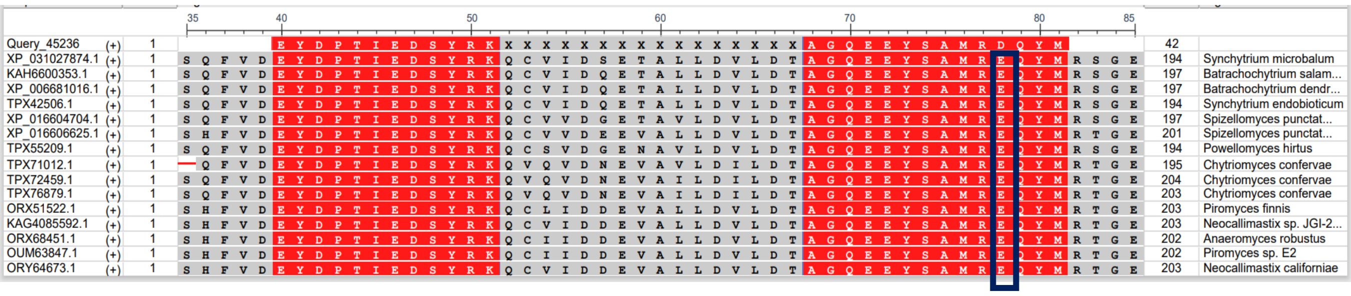

Suppl. Fig. 5
