# Supplementary material for: Origin and Evolution of RAS Oncoprotein Membrane Targeting
Source: Res Sq. 2023 Jan 20:rs.3.rs-2485219. Preprint. [Version 1] doi: 10.21203/rs.3.rs-2485219/v1 (PMC9882654; doi:10.21203/rs.3.rs-2485219/v1)
Supplement: Suppl. Fig 6 — Suppl. Fig. 6. The CaaX motifs of oncoproteins. Whereas the sequences predict geranylgeranylation signal (L or F terminal residues) in KRASBL, HRAS from cartilaginous fishes, and in most non-vertebrate groups they predict farnesylation for all vertebrate oncoproteins since jawed fish other than KRASBL that is not found in birds and mammals. CaaX terminal residues from BLASTP searches are shown as multiple alignment outputs. [file Suppl.Fig.6_10.1.2023.pdf]

HRAS
cartilaginous_fishes

Sequence ID	Sta	186	187	188	189	End	Organism
XP_032894763.1	(+)	C	V	L	L	189	Amblyraja radiata
XP_020371003.1	(+)	C	V	I	L	189	Rhincodon typus
XP_041053274.1	(+)	C	V	I	L	189	Carcharodon carcharias
XP_038663922.1	(+)	C	V	I	L	189	Scyliorhinus canicula
XP_007907536.1	(+)	C	V	I	L	189	Callorhinchus milii

KRASBL
vertebrates

Sequence ID	S	190	191	192	193	End	Organism
XP_032869079.1	(+)	C	I	I	L	188	Amblyraja radiata
XP_038639423.1	(+)	C	I	I	L	188	Scyliorhinus canicula
XP_020374864.1	(+)	C	I	I	L	188	Rhincodon typus
NP_001279499.1	(+)	C	I	I	L	188	Callorhinchus milii
XP_041033495.1	(+)	C	I	I	L	188	Carcharodon carcharias
XP_043915060.1	(+)	C	T	I	L	187	Protopterus annectens
XP_006014991.1	(+)	C	V	I	L	186	Alligator sinensis
XP_018083670.1	(+)	C	A	I	L	186	Xenopus laevis
NP_001081316.1	(+)	C	V	V	L	186	Xenopus laevis
XP_033816768.1	(+)	C	R	I	L	187	Geotrypetes seraphini
NP_001008034.1	(+)	C	V	I	L	186	Xenopus tropicalis
XP_040183575.1	(+)	C	V	I	L	186	Rana temporaria
XP_018430355.1	(+)	C	V	I	L	186	Nanorana parkeri
XP_028669532.1	(+)	C	T	I	L	186	Erpetoichthys calabaricus
XP_030053801.1	(+)	C	R	I	L	187	Microcaecilia unicolor
XP_030400968.1	(+)	C	V	I	L	186	Gopherus evgoodei
XP_005312877.1	(+)	C	V	I	L	186	Chrysemys picta bellii
ACO52008.1	(+)	C	V	I	L	186	Lithobates catesbeianus
KAG8567906.1	(+)	C	V	I	L	186	Engystomops pustulosus
XP_025918807.1	(+)	C	V	I	L	186	Apteryx rowi
XP_003226230.1	(+)	C	I	I	L	186	Anolis carolinensis
XP_015274046.1	(+)	C	V	I	L	186	Gekko japonicus
XP_040288950.1	(+)	C	I	I	L	186	Bufo bufo
XP_030400969.1	(+)	C	V	I	L	186	Gopherus evgoodei
XP_014344273.1	(+)	C	T	I	L	189	Latimeria chalumnae
XP_046721968.1	(+)	C	T	L	L	187	Silurus meridionalis
XP_017313498.1	(+)	C	I	L	L	188	Ictalurus punctatus
XP_017313508.1	(+)	C	I	L	L	187	Ictalurus punctatus

CEPHALOCHORDATA

Sequence ID	St	190	191	192	193	194	End	Organism
Query_66206	(+)						42	
XP_019642994.1	(+)	C	C	V	L	L	188	Branchiostoma belcheri
XP_019640055.1	(+)	C	T	I	L		181	Branchiostoma belcheri
XP_035696597.1	(+)	C	C	V	L	L	189	Branchiostoma floridae
CAH1240574.1	(+)	C	C	V	L	L	192	Branchiostoma lanceolat...
CAH1258932.1	(+)	C	T	I	L		188	Branchiostoma lanceolat...
CAH1240573.1	(+)	C	C	V	L	L	189	Branchiostoma lanceolat...
ABU49827.1	(+)	C	C	V			185	Branchiostoma lanceolat...
XP_019640053.1	(+)	C	T	I	L		188	Branchiostoma belcheri
XP_035672300.1	(+)	C	T	I	L		188	Branchiostoma floridae

ECHINODERMATA

Query_64595	(+)					42	
XP_038052654.1	(+)	C	V	L	L	187	Patiria miniata
XP_041467031.1	(+)	C	I	L	F	187	Lytechinus variegatus
XP_030848582.1	(+)	C	I	L	F	187	Strongylocentrotus purp...
XP_030848609.1	(+)	C	I	L	F	187	Strongylocentrotus purp...
XP_022105925.1	(+)	C	I	L	V	188	Acanthaster planci
XP_033102378.1	(+)	C	C	L	F	186	Anneissia japonica
XP_033638626.1	(+)	C	V	L	L	188	Asterias rubens

PROTOSTOMES

Caenorhabditis

Sequence ID	S	184	185	186	187	End	Organism
Query_15902	(+)					42	
NP_502213.3	(+)	C	Q	I	M	184	Caenorhabditis elegans
XP_003107699.1	(+)	C	Q	I	M	184	Caenorhabditis remanei
AAA28103.1	(+)	C	Q	I	M	187	Caenorhabditis elegans
XP_002633348.1	(+)	C	Q	I	M	184	Caenorhabditis briggsae

Other protostomes

XP_024506747.1	(+)	C	C	I	Q		185	Strongyloides ratti
XP_024498517.1	(+)	C	T	I	L		183	Strongyloides ratti
CAD56890.1	(+)	C	A	I	I		159	Meloidogyne artiellia
VDM57379.1	(+)	C	L	I	L		151	Angiostrongylus costaric..
XP_023233859.1	(+)	C	I	L	L		184	Centruroides sculpturatus
XP_023233858.1	(+)	C	I	L	L		189	Centruroides sculpturatus
XP_046679614.1	(+)	C	S	L	L		187	Homalodisca vitripennis
VBB25418.1	(+)	C	V	I	I		177	Acanthocheilonema viteae
VDO26248.1	(+)	C	V	I	I		183	Onchocerca flexuosa
TMS39525.1	(+)	C	I	I	L		183	Steinernema carpocapsae
RUS85085.1	(+)	C	L	L	L		185	Elysia chlorotica
RNA14479.1	(+)	C	A	L	M		187	Brachionus plicatilis
KAH9404065.1	(+)	C	V	I	L		176	Tyrophagus putrescentiae
CAD5120984.1	(+)	C	T	L	L		182	Dimorphilus gyrociliatus
XP_021345848.1	(+)	C	A	L	I		185	Mizuhopecten yessoensis
VDK61717.1	(+)	C	V	I	V		183	Onchocerca ochengi
ROT64798.1	(+)	C	I	V	F		185	Penaeus vannamei
KAG9508781.1	(+)	C	C	S	L	L	187	Fragariocoptes setiger
CAF1315476.1	(+)	C	V	L	M		177	Didymodactylos carnosus
XP_001899045.1	(+)	C	V	I	I		183	Brugia malayi
XP_003139513.1	(+)	C	V	I	I		183	Loa loa
KAH3885852.1	(+)	C	G	M	L		184	Dreissena polymorpha
XP_033751829.1	(+)	C	A	L	I		185	Pecten maximus
XP_045175893.1	(+)	C	A	V	L		184	Mercenaria mercenaria
CAD5218544.1	(+)	C	N	I	L		183	Bursaphelenchus okina...
VDN18617.1	(+)	C	T	I	I		177	Gongylonema pulchrum
XP_022342722.1	(+)	C	R	L	F		185	Crassostrea virginica
KOF85932.1	(+)	C	V	L	L		174	Octopus bimaculoides
XP_017489004.1	(+)	C	V	I	L		187	Rhagoletis zephyria
ACU33971.1	(+)	C	R	L	F		184	Crassostrea angulata
ABA82136.1	(+)	C	E	L	L		187	Tritia reticulata
XP_011447097.1	(+)	C	R	L	F		184	Crassostrea gigas
RWS07440.1	(+)	C	S	I	L		186	Dinothrombium tinctorium
XP_022656883.1	(+)	C	T	I	F		188	Varroa destructor
XP_041366683.1	(+)	C	I	L	F		187	Gigantopelta aegis
KAF7495709.1	(+)	C	H	I	L		187	Sarcoptes scabiei
OTF69949.1	(+)	C	I	I	L		160	Euroglyphus maynei
XP_027229439.1	(+)	C	I	V	F		187	Penaeus vannamei
GFY15157.1	(+)	C	V	I	L		187	Trichonephila clavipes
XP_023325514.1	(+)	C		I	Q		183	Eurytemora affinis
XP_022197810.1	(+)	C	H	L	F		188	Nilaparvata lugens
AET71737.1	(+)	C	I	V	F		187	Penaeus vannamei
VDK59030.1	(+)	C	T	I	L		183	Anisakis simplex
XP_045120058.1	(+)	C	I	V	F		187	Portunus trituberculatus
KAG8336268.1	(+)	C	H	L	L		164	Homalodisca vitripennis

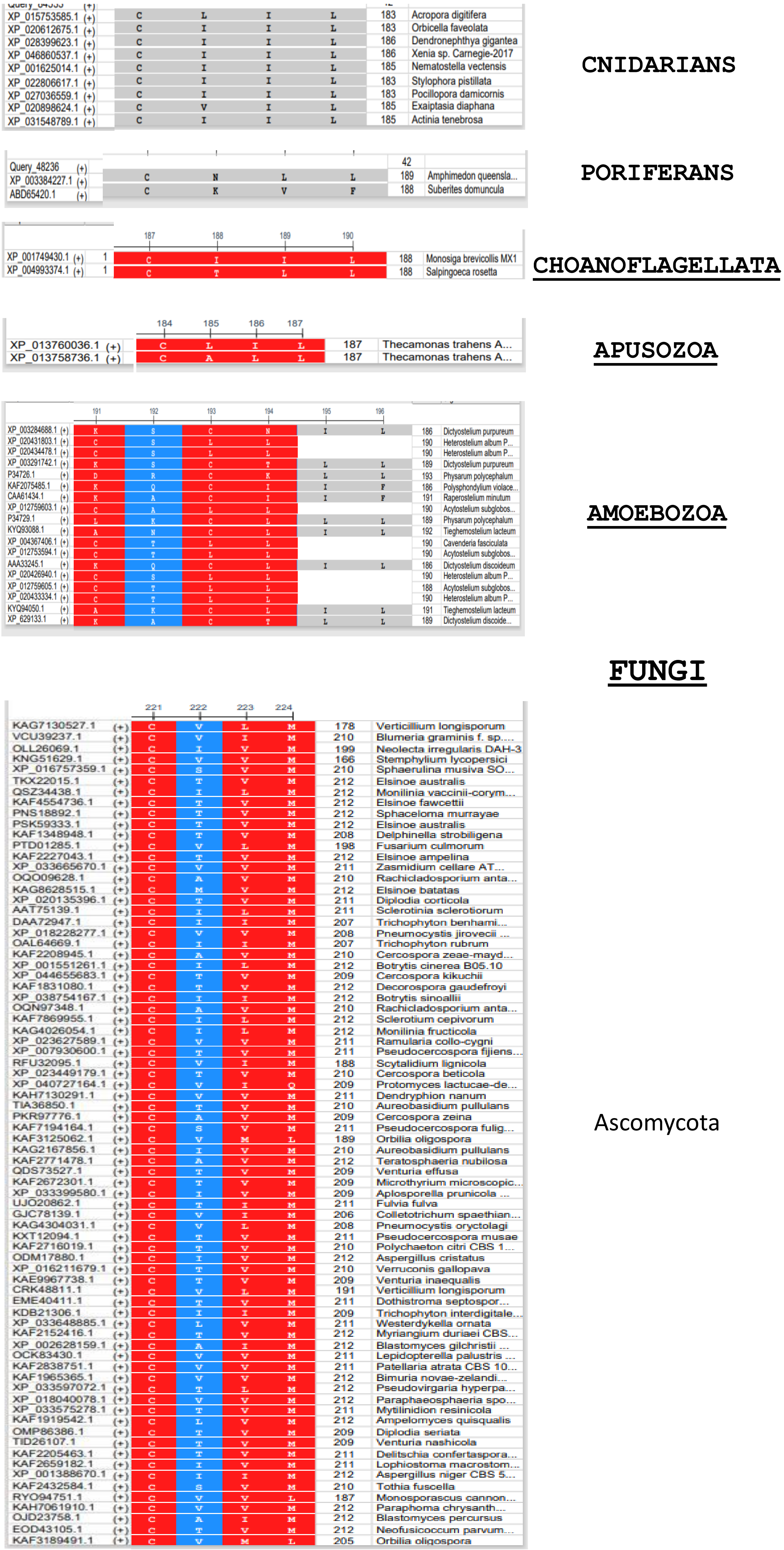

CNIDARIANS
PORIFERANS
CHOANOFLAGELLATA
APUSOZOA
AMOEBOZOA
FUNGI
Ascomycota

Neocallimastigomycota

		201	202	203	204		
Query_62834	(+)					42	
ORX51522.1	(+)	C	V	V	M	203	Piromyces finnis
KAG4085592.1	(+)	C	I	V	M	203	Neocallimastix sp. JGI-2...
ORX68451.1	(+)	C	V	V	M	202	Anaeromyces robustus
OUM63847.1	(+)	C	V	V	M	202	Piromyces sp. E2
ORY64673.1	(+)	C	V	V	M	203	Neocallimastix californiae

Zoopagomycota

		212	213	214	215	216		
PVU88987.1	(+)	K	C	L	I	L	207	Smittium simulii
RKP23521.1	(+)	C	C	V	I	M	208	Syncephalis pseudoplum...
KAF7751656.1	(+)	C	M	I	L		198	Entomophthora muscae
PIA15019.1	(+)	S	C	V	I	L	208	Coemansia reversa NR...
OMJ19944.1	(+)	K	C	I	I	L	207	Smittium culicis
KXN72412.1	(+)	C	T	I	L		206	Conidiobolus coronatus...
RKP11788.1	(+)	C	C	I	L	M	206	Piptocephalis cylindrosp...
KAF7724297.1	(+)	C	T	I	L		203	Entomophthora muscae
KXN72056.1	(+)	C	I	I	M		199	Conidiobolus coronatus...
RKP06782.1	(+)	C	C	V	V	M	207	Thamnocephalis sphaer...

Blastocladiomycota

		206	207	208	209		
KNE58963.1	(+)	C	I	V	M	206	Allomyces macrogynus ...
KNE57743.1	(+)	C	I	V	M	206	Allomyces macrogynus ...
ORZ41256.1	(+)	C	L	V	Q	206	Catenaria anguillulae P...

Basidiomycota

		292	293	294	295		
Query_9872	(+)					42	
XP_007417756.1	(+)	C	V	I	L	213	Melampsora larici-popul...
KAG0150143.1	(+)	C	V	I	L	215	Cronartium quercuum f....
CBQ70390.1	(+)	C	V	V	L	215	Sporisorium reilianum ...
SJX61064.1	(+)	C	V	V	L	215	Sporisorium reilianum f....
XP_041415501.1	(+)	C	V	V	L	216	Ustilago hordei
AAG25584.1	(+)	C	V	V	L	210	Cryptococcus neoforma...
KAH9814012.1	(+)	C	V	I	L	213	Melampsora americana
XP_003331739.1	(+)	C	L	I	L	214	Puccinia graminis f. sp. tr...
XP_025598673.1	(+)	C	V	V	L	215	Tilletiopsis washingtone...
CDI51925.1	(+)	C	V	I	L	215	Melanopsichium pennsy...
MBW0567331.1	(+)	C	I	I	L	214	Austropuccinia psidii MF-1
XP_014658739.1	(+)	C	V	V	L	215	Moesziomyces antarcticus
GAC72788.1	(+)	C	V	V	L	215	Moesziomyces antarctic...
XP_007876738.1	(+)	C	V	V	L	215	Pseudozyma flocculosa...
KAH8916037.1	(+)	C	V	L	L	213	Atractiella rhizophila
POV99298.1	(+)	C	V	I	L	211	Puccinia striiformis
PWN51446.1	(+)	C	V	A	M	215	Violaceomyces palustris
XP_001730287.1	(+)	C	V	T	M	216	Malassezia globosa C...
SCZ99053.1	(+)	C	V	I	L	211	Microbotryum saponariae
KDE06001.1	(+)	C	V	I	L	211	Microbotryum lychnidis...
SHO77137.1	(+)	C	I	V	M	206	Malassezia sympodialis...
XP_029737119.1	(+)	C	V	V	L	215	Sporisorium graminicola
XP_011386972.1	(+)	C	I	V	L	215	Ustilago maydis 521
XP_016276872.1	(+)	C	V	I	L	208	Rhodotorula toruloides ...
CDR99351.1	(+)	C	V	V	L	215	Sporisorium scitamineum
SPO22713.1	(+)	C	V	V	L	215	Ustilago trichophora
KAH9464865.1	(+)	C	V	I	L	214	Puccinia striiformis f. sp....
PWZ01038.1	(+)	C	V	V	L	215	Testicularia cyperi
XP_016291390.1	(+)	C	V	V	L	215	Kalmanozyma brasiliensis..
SGY46944.1	(+)	C	V	I	L	218	Microbotryum silenes-dio...
XP_027484897.1	(+)	C	V	I	I	214	Malassezia restricta
XP_025378146.1	(+)	C	V	V	L	212	Acaromyces ingoldii
OAV91324.1	(+)	C	V	I	L	214	Puccinia triticina 1-1 BB...
PLW13352.1	(+)	C	V	I	L	214	Puccinia coronata f. sp. ...
XP_025367309.1	(+)	C	V	V	L	215	Ceraceosorus guamensis
ORY79342.1	(+)	C	V	I	L	219	Leucosporidium creatini...
XP_025352821.1	(+)	C	M	V	L	209	Meira miltonrushii
AYO43789.1	(+)	C	V	I	I	214	Malassezia restricta C...
KNF01677.1	(+)	C	V	I	L	214	Puccinia striiformis f. sp....
XP_025362674.1	(+)	C	R	V	M	221	Jaminaea rosea
POW16259.1	(+)	C	V	I	L	215	Puccinia striiformis
XP_025349451.1	(+)	C	K	V	M	217	Pseudomicrostroma glu...
KAE8190230.1	(+)	C	V	V	M	218	Tilletia laevis
XP_009266395.1	(+)	C	V	V	L	209	Wallemia ichthyophaga...
TIA95827.1	(+)	C	V	V	L	209	Wallemia ichthyophaga
KAE8209737.1	(+)	C	V	V	M	216	Tilletia walkeri
TIA91121.1	(+)	C	V	V	L	215	Wallemia hederae
XP_006956575.1	(+)	C	V	V	L	209	Wallemia mellicola CBS...
TIC26897.1	(+)	C	V	V	L	213	Wallemia mellicola
TIB82368.1	(+)	C	V	V	L	213	Wallemia mellicola
TIC70550.1	(+)	C	V	V	L	208	Wallemia mellicola
TIB75173.1	(+)	C	V	V	L	208	Wallemia mellicola
KLO13067.1	(+)	C	V	I	L	213	Schizopora paradoxa
KZV91444.1	(+)	C	V	V	A	217	Exidia glandulosa HHB1...
KAF9532591.1	(+)	C	V	V	L	215	Crepidotus variabilis
AGC26948.1	(+)	C	V	V	V	212	Polyporus umbellatus
KAF8199655.1	(+)	C	V	V	L	215	Pholiota molesta
KAF9466544.1	(+)	C	V	V	L	215	Lepista nuda
TRM64777.1	(+)	C	V	V	L	212	Auriculariopsis ampla
PPQ83429.1	(+)	C	V	V	L	211	Psilocybe cyanescens
KAF5342148.1	(+)	C	V	V	L	215	Tulosesus angulatus
KIJ51063.1	(+)	C	V	V	A	213	Sphaerobolus stellatus ...
KAH7912336.1	(+)	C	V	V	L	215	Hygrophoropsis auranti...
XP_041243132.1	(+)	C	V	V	V	215	Suillus subalutaceus
KAF8968019.1	(+)	C	V	V	L	215	Flammula alnicola
XP_019001200.1	(+)	C	V	V	L	202	Kwoniella mangroviens...
KAF7330306.1	(+)	C	V	V	L	214	Mycena venus
AAD55937.1	(+)	C	V	V	L	210	Cryptococcus neoforma...
KIM54372.1	(+)	C	V	V	V	214	Scleroderma citrinum F...
KAF8150758.1	(+)	C	V	V	L	215	Crassisporium funariophil...
KIJ23822.1	(+)	C	V	V	A	214	Sphaerobolus stellatus ...
XP_021870724.1	(+)	C	V	V	L	212	Kockovaella imperatae
KAH9481657.1	(+)	C	V	V	L	215	Psilocybe cubensis
KAF8519289.1	(+)	C	I	V	A	215	Gautieria morchelliformis
OCF74603.1	(+)	C	V	V	L	202	Kwoniella mangroviens...
XP_003031708.1	(+)	C	V	V	L	212	Schizophyllum commu...
KAH9974825.1	(+)	C	V	V	A	214	Russula compacta
KDR78898.1	(+)	C	V	V	L	215	Galerina marginata CBS...
KAG6331600.1	(+)	C	V	V	V	215	Astraeus odoratus
KIY64562.1	(+)	C	V	V	L	213	Cylindrobasidium torre...
KJA26361.1	(+)	C	V	V	L	214	Hypholoma sublateritium...

Chytridiomycota

		219	220	221	222		
XP_031027874.1	(+)	C	E	I	M	194	Synchytrium microbalum
KAH6600353.1	(+)	C	L	L	M	197	Batrachochytrium salam...
XP_006681016.1	(+)	C	L	L	M	197	Batrachochytrium dendr...
TPX42506.1	(+)	C	D	I	M	194	Synchytrium endobioticum
XP_016604704.1	(+)	C	M	V	M	197	Spizellomyces punctat...
XP_016606625.1	(+)	C	T	I	L	201	Spizellomyces punctat...
TPX55209.1	(+)	C	M	I	M	194	Powellomyces hirtus
TPX71012.1	(+)	C	S	I	V	195	Chytriomyces confervae
TPX72459.1	(+)	C	S	I	L	204	Chytriomyces confervae
TPX76879.1	(+)	C	S	I	V	203	Chytriomyces confervae
ORX51522.1	(+)	C	V	V	M	203	Piromyces finnis
KAG4085592.1	(+)	C	I	V	M	203	Neocallimastix sp. JGI-2...
ORX68451.1	(+)	C	V	V	M	202	Anaeromyces robustus
OUM63847.1	(+)	C	V	V	M	202	Piromyces sp. E2
ORY64673.1	(+)	C	V	V	M	203	Neocallimastix californiae

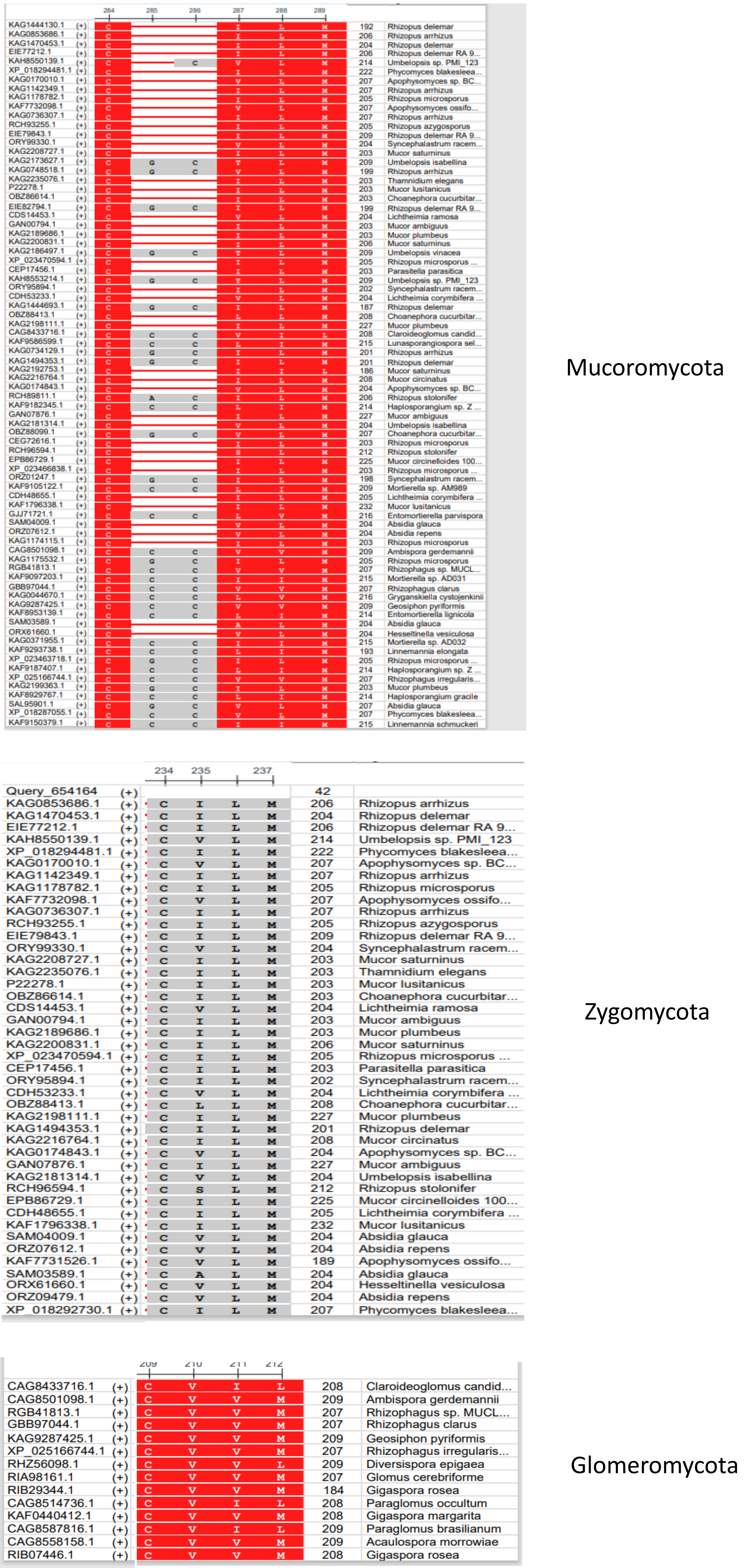
Mucoromycota
234 235 237
Query_654164 (+) 42
KAG0853686.1 (+) C I L M 206 Rhizopus arrhizus
KAG1470453.1 (+) C I L M 204 Rhizopus delemar
EIE77212.1 (+) C I L M 206 Rhizopus delemar RA 9...
KAH8550139.1 (+) C V L M 214 Umbelopsis sp. PMI_123
XP_018294481.1 (+) C I L M 222 Phycomyces blakesleea...
KAG0170010.1 (+) C V L M 207 Apophysomyces sp. BC...
KAG1142349.1 (+) C I L M 207 Rhizopus arrhizus
KAG1178782.1 (+) C I L M 205 Rhizopus microsporus
KAF7732098.1 (+) C V L M 207 Apophysomyces ossifo...
KAG0736307.1 (+) C I L M 207 Rhizopus arrhizus
RCH93255.1 (+) C I L M 205 Rhizopus azygosporus
EIE79843.1 (+) C I L M 209 Rhizopus delemar RA 9...
ORY99330.1 (+) C V L M 204 Syncephalastrum racem...
KAG2208727.1 (+) C I L M 203 Mucor saturninus
KAG2235076.1 (+) C I L M 203 Thamnidium elegans
P22278.1 (+) C I L M 203 Mucor lusitanicus
OBZ86614.1 (+) C I L M 203 Choanephora cucurbitar...
CDS14453.1 (+) C V L M 204 Lichtheimia ramosa
GAN00794.1 (+) C I L M 203 Mucor ambiguus
KAG2189686.1 (+) C I L M 203 Mucor plumbeus
KAG2200831.1 (+) C I L M 206 Mucor saturninus
XP_023470594.1 (+) C I L M 205 Rhizopus microsporus ...
CEP17456.1 (+) C I L M 203 Parasitella parasitica
ORY95894.1 (+) C I L M 202 Syncephalastrum racem...
CDH53233.1 (+) C V L M 204 Lichtheimia corymbifera ...
OBZ88413.1 (+) C L L M 208 Choanephora cucurbitar...
KAG2198111.1 (+) C I L M 227 Mucor plumbeus
KAG1494353.1 (+) C I L M 201 Rhizopus delemar
KAG2216764.1 (+) C I L M 208 Mucor circinatus
KAG0174843.1 (+) C V L M 204 Apophysomyces sp. BC...
GAN07876.1 (+) C I L M 227 Mucor ambiguus
KAG2181314.1 (+) C V L M 204 Umbelopsis isabellina
RCH96594.1 (+) C S L M 212 Rhizopus stolonifer
EPB86729.1 (+) C I L M 225 Mucor circinelloides 100...
CDH48655.1 (+) C I L M 205 Lichtheimia corymbifera ...
KAF1796338.1 (+) C I L M 232 Mucor lusitanicus
SAM04009.1 (+) C V L M 204 Absidia glauca
ORZ07612.1 (+) C V L M 204 Absidia repens
KAF7731526.1 (+) C V L M 189 Apophysomyces ossifo...
SAM03589.1 (+) C A L M 204 Absidia glauca
ORX61660.1 (+) C V L M 204 Hesseltinella vesiculosa
ORZ09479.1 (+) C V L M 204 Absidia repens
XP_018292730.1 (+) C I L M 207 Phycomyces blakesleea...
Zygomycota
CAG8433716.1 (+) C V I L 208 Claroideoglomus candid...
CAG8501098.1 (+) C V V M 209 Ambispora gerdemannii
RGB41813.1 (+) C V V M 207 Rhizophagus sp. MUCL...
GBB97044.1 (+) C V V M 207 Rhizophagus clarus
KAG9287425.1 (+) C V V M 209 Geosiphon pyriformis
XP_025166744.1 (+) C V V M 207 Rhizophagus irregularis...
RHZ56098.1 (+) C V V L 209 Diversispora epigaea
RIA98161.1 (+) C V V M 207 Glomus cerebriforme
RIB29344.1 (+) C V V M 184 Gigaspora rosea
CAG8514736.1 (+) C V I L 208 Paraglomus occultum
KAF0440412.1 (+) C V V M 208 Gigaspora margarita
CAG8587816.1 (+) C V I L 209 Paraglomus brasilianum
CAG8558158.1 (+) C V V M 209 Acaulospora morrowiae
RIB07446.1 (+) C V V M 208 Gigaspora rosea
Glomeromycota

Suppl. Fig. 6
