# Supplementary material for: Origin and Evolution of RAS Oncoprotein Membrane Targeting
Source: Res Sq. 2023 Jan 20:rs.3.rs-2485219. Preprint. [Version 1] doi: 10.21203/rs.3.rs-2485219/v1 (PMC9882654; doi:10.21203/rs.3.rs-2485219/v1)
Supplement: Suppl. Fig 8 — Suppl. Fig. 8. Appearance of KRAS Exon 4A in vertebrates. Genomic regions of shark, hagfish and lamprey showing the locations of exons 4B (blue letters) and 4A (green letters). Stop codons are highlighted in red. Genomic regions: shark NW_006890288.1 (547536..553042); hagfish, Eburgeri 3.2: FYBX02009586.1: 2433624:2434677:1; lamprey Chr. 65: NC_046133.1, 5454894 to 5455895. GenBank assembly accession numbers: shark, GCF_000165045.1; hagfish GCA_900186335.2; lamprey, GCF_010993605.1. [file Suppl.Fig.8_10.1.2023.pdf]

Elephant Shark *kras*

Exon 3

AGAACAGATAAAAAGGGTAAAAGACTCTGATGACGTGCCAATGGTCCTCGTGGGAAATAAATGTGATTTG
CCTTCAAGAACAGTAGACACAAGACAAGCACAGGAACTAGCGAGAAGTTACGGAATTCCATTCATTGAAA
CGTCAGCGAAAACAAGACAAgtgagtgaaatggggggaaactgtcatggcctgtgtggatgggctttgttcttaatgg
aatcattgccagtgattgaatgtgaactttagcgacttgcacaactgactgctattgttagttacttacacgtatgtca
cttgcacttggcaaagcacaaaggcagcaacaacaaatctcatttccacagagcctttcaagtcgggaggacgtcccaa
ggcgctggacagtcaggggtttggacccgagctgggggttgggatcaggaggggtggagggaagggagggtgaggggta
ggaaggtgaccgatggcgcagtcgaagaggagggtcttgagagcaattttcaattttcctttctgtgaaatatccattc
ttttaagttgtgagactctcattgaaaagtgatttttttttcctcaagactaagagaaaaaataggtttcagtttggttc
acttggtagcactcgtgtctctgggtcagaagcttgtggattcaagcccccaaatcaacaacaacaacaacttggattt
atatcgcgccctctacgccggacggcgtctcagagcgctttacagtaacagatcccggggccgagccggggggaggggg
aggaagatgaccaaaggcgcggtcgaaaagaaagggttgagtgcacagtctaggctggcaatccagtgcagtactgggg
gtttgtgcgttgtcgagagttggcatccttcacgtctgatgttaaaccaagttcttgtctgcctgttcaggtggatatg
aaagatgtttaaaagagatgaatttctcctgttatcctggtccacagagccagagataaattggacattcttataatgg
ctgttttgtaatgcggatttccgaaaattagaattaaaaaaaagtactgctgcatgctctacaataacgatatcgctta
gtcctttggcttgttagaggtatctacgttacattaattaacaggtcttaacagcgacttgagcgcttttgtggaactt
tcactttctttgccatcgtgaataggcataactgatcttgaaagcagttttagatcccgtgtctacgttgcagcgtagc
aggaataatatcttaagatttataatgtcatttggtgcttgataaacctctagcttaacgaagctttgttcacaagtat
ttatttgacctttccttgagagctgttgattagtaataagtagaggcttaaacattatattaattgattaaattggtat
cttcgggactgaaatcaacctctctaatggtggcggtggttctcttagaaatctctgcatggctaaaatcacaactcct
cgttttatgtgttggtgacgggcagaccgaccggcgaatcgagatgattcaatattctcaaaagatttttttgagaaaa
aaaatcagcactgggggaaaagaaatgtgaatcattttgtaacgtttctgtcaaatgctgattgagggagttgtgtttt

Exon 4A

gcaatgcagAGAGTGGAGGATGCATTTTATACACTGGTACGTGAGATTCGCCAATACAGGTTGAGAAAGCT
CAACACTGAAGAAAAGACAACCAGATGCGTGGTGTTTAAATGTGTTGTGATGTAACGGGgtaagtggtgaat
ttctacaggcaagagcttccttcaggcaaggacagaatacctgtctttaaaccgtttcgaatatataataatccttgta
tttggtatcgcgcctcatcacctcttcgagacgtctcacagcgcttcacagaataaactgtcaagtgaattaactgtat
ttttttgtggacgaaacgcacagcagtgtcccgcaaacagccgtggattgagcgaccaaattcttttatgatgtttt
tttggggaatcattttattgaagagtttattacccccggaaaagcgatccaccaatcgctcccccccctccccaaattat
tataataaaaaataataataatgttgtcactcaatccacggctgtttgtggggacgctgctgtgcgcaattggctgccg
catttcgcccacaaaataccgtatattctgtgaagcgctttgagatgtctcaatgacatgataaggcgctgtaacaagt
acaaggattattatgttgaaagtcaacactattcgagaagagtttctcctggtatcctggccagcacttcttgagttat
tgattattgattgattattcacctcatggcggtgtgggggaatttgcaatgtataaagtgttaattttgttgagcttac
aacaaaatcctcaaagtgcttcacaatttacatttcacacattattgtgaagttctttgaggagttttgacttctaggc
tcaatacaaatggcagttctttcttgggtgttttttttccccaacatttaatattcctctttatcaagaatcatttttt
ttcgcaagagttagacaccatttcaactgaacgtcaacaaaacccgatgtgtacctgtgtgaaactttaaaaccttcaa
cacacatgaagattaatacagaaggtcgagaccatgttgatttcttttaaaaattctattcatatcagaacgttgattg
atgaaattggacttttgtactgaaaaatggaacattgtggagggaaatgttttgccaaagaagcttttcgagagcaata
ttctcccacaatgaatccatttgacagcgtatcctgtgaagtgcgttgagacatttcaacgatgtgatagggcactaga
tcaaatgcaaggattattattaatatttagctggaacattagatgttgcatgttatacctgataattgcagtgtagtaa
tttcaatctcagcaaagtatactttggagcagttaatgtacttgtgagttaaatatcacaaaggaacgcaccttgcata

Exon 4B

ttcaacagtggtaggtgattgatactgataacatactgactctgtgtgtgtgtgttttacttacagGGCGTTGACGAC
GCTTTCTACACACTAGTCCGCGAAATCCGAAAGCACAAAGAGAAAATGAGTAAAGACGGCAAAAAGAAGA
AAAATAAAACCAAGAAAAAGTGTTCGATTATGTGA

Hagfish *kras*

Exon 3

GGAACAGATAAAGAGAGTGAAAGATTCGGAAGATGTCCCCATGGTTTTGGTTGGAAACAAATGTGACCTC
CCGACCCGTACTGTTGACACCAAGCAAGCACTTGACCTTGCACGATCTTTTGGTATCCCTTGTATTGAGA
CCTCAGCCAAGACTCGGCAGgtgagccaatcgacgggctttggtgtttgataaagtttaccttctggaaaatactaa
aaactcagatcatcttgcttgtgaattggaaactagaaacgtcaatttgtgggagt*gggggggggggggggggggggg*
*gggggggggggggggggggggggggggggggggggggggggggggg*ggtggtgcctcaaatgattttcctttttct
tctcaattctcccagaattgtccatcttctttgcactcgtgatatactgtggttttgagtttggcattttcccagaagt
tccacattaaccgtaaatgctaaagcactgattatagtggtcctttttttgagttgatgcttgctcaagctgttcaggct
ttatgtatttgatgtgcgcacaaactccctgaatgcgagactgccattatatcctagtttctcactcttccctggttgt

Exon 4B

ctatttcctttttattttcttatagGGGGTGGACGATGCCTTTTATACGCTTGTCCGTGAAATCCGCAAGTACA
AGGAACGCGCAAGCAAGGATGGGAAAAAGAAAAAGAAGAGATCTCGAAAAATGTGCTTGGTCATGTGA

Lamprey *kras*

Exon 3

GGAACAGATCAAGCGCGTCAAGGACTCTGAGGACGTGCCGATGGTGCTGGTGGGTAACAAGTGTGACCTG
CCCACGCGGACCGTGGACACGAAGCAGGCGCAGGAGCTGGCACGCAGCTACGGCATCCCCTTCATAGAGA
CCTCCGCCAAGACTCGGCAGgtaggcgggggggaggggggggcgagggggcgggggggaggtgggggcagctgtggg
ggggggtggttggggagtgtccaggggggagtaaggggcggctggtgggggtcccgtggcgtgccgcaccacgaccccgg
cgacccgggggggtttcgattcccgcgtgcctgggatgccgcctggccacgtggccgtgtgtccgcgtgtccgcgtagtg
tgtgtgtgcgaggctatgggtgttcgccctgcgtgtctgcgtgtccgtgtgtccgcgtgtcccgcgcgtgtgtgcgagg
ctatgggtgttggtcctgcgtgccgcgtgtccgcgtgtccgtgtgtccgtgcgtgtgtaaggctatcggtgttggcctg
cgtgtctgcctgtccgtgtgtccgtgtgtccgtgtgtgtgtaaggctatgggtgttcgccctgcgtgtctgcgtgtccg
tgtgtgtgcgaggctatgggtgttggccctgtgtgtccgcgtgtccgcgtgtgtgcgaggctatgggtgttcgccctgc
gtgtctgtgtgtccgtgtgtccgcctgtctgcgtgtgtgtgcgaggctccggtgttggccccccgcgtgtccgtgcgcg
tctctgcgcacgagcccctcccgtcgtcccgcatgagagcgtgagg***ccccccccccccccgcccccccccccc***tctaa

Exon 4B

cgagaggctccgctgtgtgcgtgccccctgctgcagGGCGTGGACGACGCCTTCTACACCCTGGTGCGGGAGAT
CCGCAAGTACAAGGACCGCGTGAGTAAAGACGGGCGGAAGAAGAAAAAGAAAATCGAAAAAGAAGTGCCT
CCTCATGTAA

Suppl. Fig. 8
