# Supplementary material for: Origin and Evolution of RAS Oncoprotein Membrane Targeting
Source: Res Sq. 2023 Jan 20:rs.3.rs-2485219. Preprint. [Version 1] doi: 10.21203/rs.3.rs-2485219/v1 (PMC9882654; doi:10.21203/rs.3.rs-2485219/v1)
Supplement: Suppl. Fig 9 — Suppl. Fig. 9. Alternative carboxyl terminal splicing variants in the Ras superfamily. NCBI sequences with exons in alternate colors, and screen captures of the genomic gene structures with the spliced exons indicated by arrows. Time of appearance of isoforms was deduced from NCBI protein BLAST searches. Isoforms as described in the Uniprot server that are produced by alternative splicing affecting the carboxyl terminal region are shown in the box. [file Suppl.Fig.9_10.1.2023.pdf]

Kras

Alternative splicing exon skipping

isoform 4A first appears in carthilaginous fishes

human.KRAS4B NP_001356716.1 GTPase KRas isoform b [Homo sapiens]
MTEYKLVVVGAGGVGKSALTIQLIQNHFVDEYDPTIEDSYRKQVVIDGETCLLDILDTAGQEEYSAMRDQ
YMRTGEGFLCVFAINNTKSFEDIHHYREQIKRVKDSEDVPMVLVGNKCDLPSRTVDTKQAQDLARSYGIP
FIETSAKTRQGVDDAFYTLVREIRKHKEKMSKDGKKKKKKSKTKCVIM

Exon 4B

human.KRAS4A NP_001356715.1 GTPase KRas isoform a [Homo sapiens]
MTEYKLVVVGAGGVGKSALTIQLIQNHFVDEYDPTIEDSYRKQVVIDGETCLLDILDTAGQEEYSAMRDQ
YMRTGEGFLCVFAINNTKSFEDIHHYREQIKRVKDSEDVPMVLVGNKCDLPSRTVDTKQAQDLARSYGIP
FIETSAKTRQRVEDAFYTLVREIRQYRLKKISKEEKTPGCVKIKKCIIM

Exon 4A

Isoform 2A (identifier: **P01116-1**) [UniParc] FASTA Add to basket
Also known as: K-Ras4A
This isoform has been chosen as the canonical[i] sequence. All positional information in this downloadable versions of the entry.
« Hide

```
        10         20         30         40         50
MTEYKLVVVG AGGVGKSALT IQLIQNHFVD EYDPTIEDSY RKQVVIDGET
        60         70         80         90        100
CLLDILDTAG QEEYSAMRDQ YMRTGEGFLC VFAINNTKSF EDIHHYREQI
       110        120        130        140        150
KRVKDSEDVP MVLVGNKCDL PSRTVDTKQA QDLARSYGIP FIETSAKTRQ
       160        170        180
RVEDAFYTLV REIRQYRLKK ISKEEKTPGC VKIKKCIIM
```

Isoform 2B (identifier: **P01116-2**) [UniParc] [UniParc] FASTA Add to basket
Also known as: K-Ras4B
The sequence of this isoform differs from the canonical sequence as follows:
151-153: RVE → GVD
165-189: QYRLKKISKEEKTPGCVKIKKCIIM → KHKEKMSKDGKKKKKKSKTKCVIM

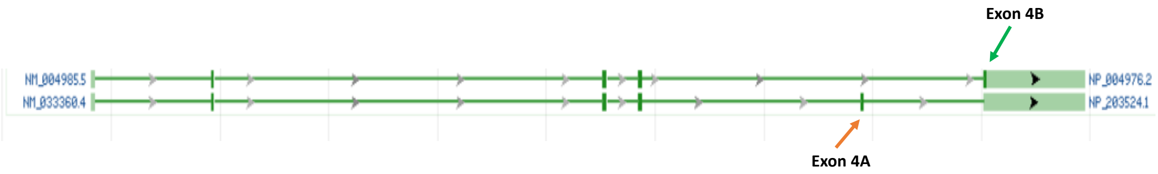

CDC42 Rho family

Alternative splicing exon skipping

isoform 2 NP_426359 first appears in cartilaginous fish

NP_001782.1 NM_001791.4 cell division control protein 42 homolog isoform 1 precursor [Homo sapiens]
MQTIKCVVVGDGAVGKTCLLISYTTNKFPSEYVPTVFDNYAVTVMIGGEPYTLGLFDTAGQEDYDRLRPL
SYPQTDVFLVCFSVVSPSSFENVKEKWVPEITHHCPKTPFLLVGTQIDLRDDPSTIEKLAKNKQKPITPE
TAEKLARDLKAVKYVECSALTQKGLKNVFDEAILAALEPPEPKKSRRCVLL

Exon 6

NP_426359.1 NM_044472.3cell division control protein 42 homolog isoform 2 [Homo sapiens]
MQTIKCVVVGDGAVGKTCLLISYTTNKFPSEYVPTVFDNYAVTVMIGGEPYTLGLFDTAGQEDYDRLRPL
SYPQTDVFLVCFSVVSPSSFENVKEKWVPEITHHCPKTPFLLVGTQIDLRDDPSTIEKLAKNKQKPITPE
TAEKLARDLKAVKYVECSALTQRGLKNVFDEAILAALEPPETQPKRKCCIF

Exon 5

Isoform 2 (identifier: **P60953-2**) [UniParc] FASTA Add to basket
Also known as: Placental
This isoform has been chosen as the canonical[i] sequence. All positional in entry.
« Hide

```
        10         20         30         40         50
MQTIKCVVVG DGAVGKTCLL ISYTTNKFPS EYVPTVFDNY AVTVMIGGEP
        60         70         80         90        100
YTLGLFDTAG QEDYDRLRPL SYPQTDVFLV CFSVVSPSSF ENVKEKWVPE
       110        120        130        140        150
ITHHCPKTPF LLVGTQIDLR DDPSTIEKLA KNKQKPITPE TAEKLARDLK
       160        170        180        190
AVKYVECSAL TQKGLKNVFD EAILAALEPP EPKKSRRCVL L
```

Isoform 1 (identifier: **P60953-1**) [UniParc] [UniParc] FASTA Add
Also known as: Brain
The sequence of this isoform differs from the canonical sequence as follow
163-163: K → R
182-191: PKKSRRCVLL → TQPKRKCCIF

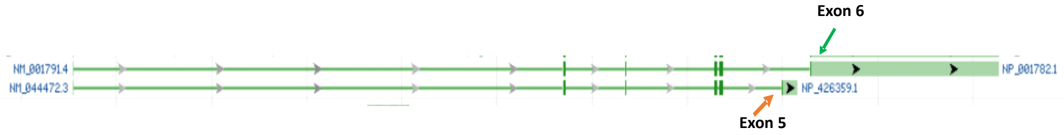

Polarizing the Neuron through Sustained Co-expression of Alternatively Spliced Isoforms.
Yap K, Xiao Y, Friedman BA, Je HS, Makeyev EV.
Cell Rep. 2016 May 10;15(6):1316-28. doi: 10.1016/j.celrep.2016.04.012. Epub 2016 Apr 28.

Hras

Alternative splicing exon skipping

isoform 2 NP_789765 (H-RasIDX) only in placental mammals

human.HRAS NP_001123914.1 |NM_001130442.3GTPase HRas isoform 1 [Homo sapiens]
MTEYKLVVVGAGGVGKSALTIQLIQNHFVDEYDPTIEDSYRKQVVIDGETCLLDILDTAGQEEYSAMRDQYMRTG
EGFLCVFAINNTKSFEDIHQYREQIKRVKDSDDVPMVLVGNKCDLAARTVESRQAQDLARSYGIPYIETSAKTRQ
GVEDAFYTLVREIRQHKLRKLNPPDESGPGCMSCKCVLS

Exon 5

NP_789765.1| NM_176795.5|::1-170 GTPase HRas isoform 2 [Homo sapiens]
MTEYKLVVVGAGGVGKSALTIQLIQNHFVDEYDPTIEDSYRKQVVIDGETCLLDILDTAGQEEYSAMRDQYMRTG
EGFLCVFAINNTKSFEDIHQYREQIKRVKDSDDVPMVLVGNKCDLAARTVESRQAQDLARSYGIPYIETSAKTRQ
GSRSGSSSSGTLWDPPGPM

Exon 4

Isoform 1 (identifier: **P01112-1**) [UniParc] FASTA Add to basket
Also known as: H-Ras4A, p21
This isoform has been chosen as the canonical[i] sequence. All positional information in this entry downloadable versions of the entry.
« Hide

```
        10         20         30         40         50
MTEYKLVVVG AGGVGKSALT IQLIQNHFVD EYDPTIEDSY RKQVVIDGET
        60         70         80         90        100
CLLDILDTAG QEEYSAMRDQ YMRTGEGFLC VFAINNTKSF EDIHQYREQI
       110        120        130        140        150
KRVKDSDDVP MVLVGNKCDL AARTVESRQA QDLARSYGIP YIETSAKTRQ
       160        170        180
GVEDAFYTLV REIRQHKLRK LNPPDESGPG CMSCKCVLS
```

Isoform 2 (identifier: **P01112-2**) [UniParc] FASTA Add to basket
Also known as: H-RasIDX, p19
The sequence of this isoform differs from the canonical sequence as follows:
152-189: VEDAFYTLVREIRQHKLRKLNPPDESGPGCMSCKCVLS → SRSGSSSSGTLWDPPGPM

Show »

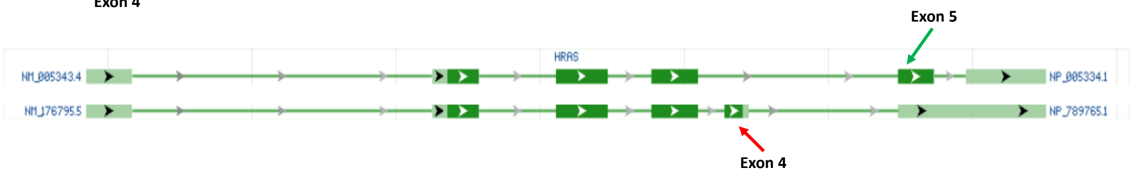

Alternative splicing of the human proto-oncogene c-H-ras renders a new Ras family protein that trafficks to cytoplasm and nucleus.
Guil S, de La Iglesia N, Fernández-Larrea J, Cifuentes D, Ferrer JC, Guinovart JJ, Bach-Elias M.
Cancer Res. 2003 Sep 1;63(17):5178-87.

Arl6   Arf family

Alternative splicing exon skipping

isoform NP_001310442 first appears ray-finned fish

```
Arl6 NP_816931.1 NM_177976.3|:1-186 ADP-ribosylation factor-like protein
6 isoform 1 [Homo sapiens]186aa
MGLLDRLSVLLGLKKKEVHVLCLGLDNSGKTTIINKLKPSNAQSQNILPTIGFSIEKFKSSSLSFTVFDM
SGQGRYRNLWEHYYKEGQAIIFVIDSSDRLRMVVAKEELDTLLNHPDIKHRRIPILFFANKMDLRDAVTS
VKVSQLLCLENIKDKPWHICASDAIKGEGLQEGVDWLQDQIQTVKT
```

Exon 6   Exon 8

```
Arl6isoform NP_001310442.1 NM_001323513.2|:1-193 ADP-ribosylation
factor-like protein 6 isoform BB3SL [Homo sapiens]
MGLLDRLSVLLGLKKKEVHVLCLGLDNSGKTTIINKLKPSNAQSQNILPTIGFSIEKFKSSSLSFTVFDM
SGQGRYRNLWEHYYKEGQAIIFVIDSSDRLRMVVAKEELDTLLNHPDIKHRRIPILFFANKMDLRDAVTS
VKVSQLLCLENIKDKPWHICASDAIKGEGLQEGVDWLQEKTIQSDPDCEDMKR
```

Exon 6   Exon 7   Exon 8

Isoform 1 (identifier: **Q9H0F7-1**) [UniParc] FASTA Add to basket

Also known as: BBS3

This isoform has been chosen as the canonical[1] sequence. All positional informat[ion …]

downloadable versions of the entry.

Hide

```
        10         20         30         40         50
MGLLDRLSVL LGLKKKEVHV LCLGLDNSGK TTIINKLKPS NAQSQNILPT
        60         70         80         90        100
IGFSIEKFKS SSLSFTVFDM SGQGRYRNLW EHYYKEGQAI IFVIDSSDRL
       110        120        130        140        150
RMVVAKEELD TLLNHPDIKH RRIPILFFAN KMDLRDAVTS VKVSQLLCLE
       160        170        180
NIKDKPWHIC ASDAIKGEGL QEGVDWLQDQ IQTVKT
```

Isoform 2 (identifier: **Q9H0F7-2**) [UniParc] FASTA Add to basket

Also known as: BBS3L

The sequence of this isoform differs from the canonical sequence as follows:

179-186: DQIQTVKT → EKTIQSDPDCEDMKR

Show »

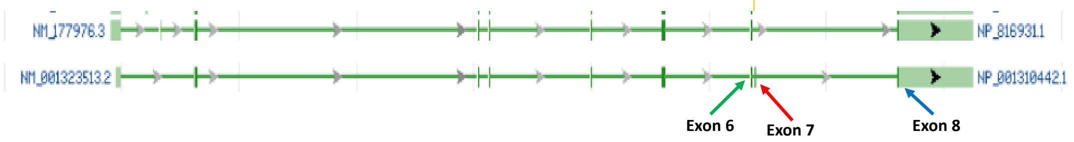

Identification and functional analysis of the vision-specific BBS3 (ARL6) long isoform.
Pretorius PR, Baye LM, Nishimura DY, Searby CC, Bugge K, Yang B, Mullins RF, Stone EM, Sheffield VC, Slusarski DC.
PLoS Genet. 2010 Mar 19;6(3):e1000884. doi: 10.1371/journal.pgen.1000884.

TRIM23/Ard1

Alternative splicing:
isoform beta   alternative 5´ splice site exón 11, stop codon exon13
isoform gamma alternative 5´ splice site exón 11, stop codon exón 12

Isoform beta   placental mammals

Isoform gamma primates (first appears in old world monkeys)

Exon 13

Beta NM_033227	NP_150230.1	CGRSWYIQGCD--ARS------VFQIICDQYTGKEVVTEKG
Alpha NM_001656	NP_001647.1	CGRSWYIQGCD--ARSGMGLYEGLDWLSRQLVAAGVL-DVA
Gamma NM_033228	NP_150231.1	CGRSCFS-------------------------------DNM

Exon 11   Exon 12

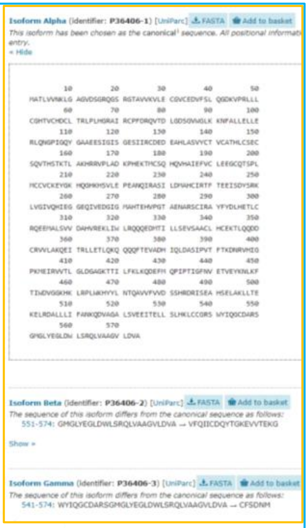

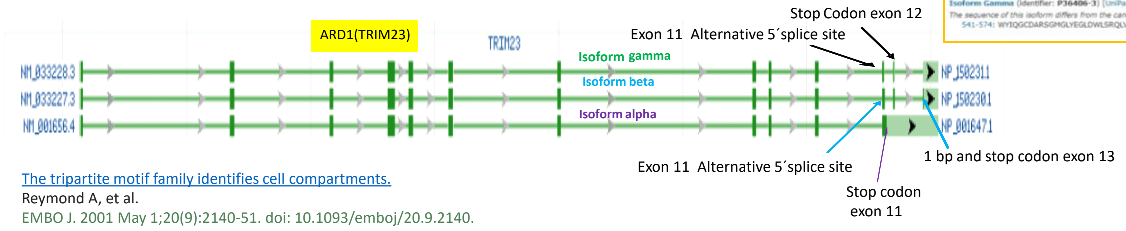

The tripartite motif family identifies cell compartments.
Reymond A, et al.
EMBO J. 2001 May 1;20(9):2140-51. doi: 10.1093/emboj/20.9.2140.

Rab8A/MEL

Alternative splicing isoform 2, alternative 3 ´splice position exon 7

Isoform 2 present only in placental mammals

```
>NP_005361.2|:P61006| NM_005370.5|:1-137 RAB8A_HUMAN Ras-related
protein Rab-8A OS=Homo sapiens OX=9606 GN=RAB8A PE=1 SV=1 207
MAKTYDYLFKLLLIGDSGVGKTCVLFRFSEDAFNSTFISTIGIDFKIRTIELDGKRIKLQIWDTA
GQERFRTITTAYYRGAMGIMLVYDITNEKSFDNIRNWIRNIEEHASADVEKMILGNKCDVNDKRQ
VSKERGEKLALDYGIKFMETSAKANINVENAFFTLARDIKAKMDKKLEGNSPQGSNQGVKITPDQ
QKRSSFFRCVLL
```

1. Exon 7

```
>P61006-2|RAB8A_HUMAN Isoform 2 of Ras-related protein Rab-8A
 OS=Homo sapiens OX=9606 GN=RAB8A 207 aa
MAKTYDYLFKLLLIGDSGVGKTCVLFRFSEDAFNSTFISTIGIDFKIRTIELDGKRIKLQIWDTA
GQERFRTITTAYYRGAMGIMLVYDITNEKSFDNIRNWIRNIEEHASADVEKMILGNKCDVNDKRQ
VSKERGEKLALDYGIKFMETSAKANINVENRYQSKNGQKIGRQQPPGEQPGSQNHTGPAEEEQLF
PMCSSVRNTALL
```

1. Exon 7 1. alternative 3 ´splice position exon 7

Isoform 1 (identifier: **P61006-1**) [UniParc] FASTA Add to basket

This isoform has been chosen as the canonical[1] sequence. All positional inform[ation …]

downloadable versions of the entry.

« Hide

```
        10         20         30         40         50
MAKTYDYLFK LLLIGDSGVG KTCVLFRFSE DAFNSTFIST IGIDFKIRTI
        60         70         80         90        100
ELDGKRIKLQ IWDTAGQERF RTITTAYYRG AMGIMLVYDI TNEKSFDNIR
       110        120        130        140        150
NWIRNIEEHA SADVEKMILG NKCDVNDKRQ VSKERGEKLA LDYGIKFMET
       160        170        180        190        200
SAKANINVEN AFFTLARDIK AKMDKKLEGN SPQGSNQGVK ITPDQQKRSS

FFRCVLL
```

Isoform 2 (identifier: **P61006-2**) [UniParc] FASTA Add to basket

The sequence of this isoform differs from the canonical sequence as follows:

161-205: AFFTLARDIK...QKRSSFFRCV → RYQSKNGQKI...PMCSSVRNTA

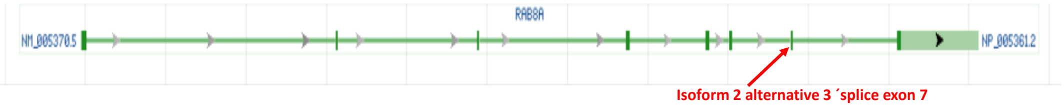

Complete sequencing and characterization of 21,243 full-length human cDNAs.
Ota T, et al.
Nat Genet. 2004 Jan;36(1):40-5. doi: 10.1038/ng1285. Epub 2003 Dec 21

Rab28

Alternative splicing mutually exclusive exons

Isoform NP_004240 appeared first in cartilaginous fish

Isoform NP_001153073 appeared first in placental mammals

```
>NP_001017979.1| NM_001017979.3:1-221 ras-related protein Rab-28 isoform 1 [Homo
sapiens]221aa
MSDSEEESQDRQLKIVVLGDGASGKTSLTTCFAQETFGKQYKQTIGLDFFLRRITLPGNLNVTLQIWDIG
GQTIGGKMLDKYIYGAQGVLLVYDITNYQSFENLEDWYTVVKKVSEESETQPLVALVGNKIDLEHMRTIK
PEKHLRFCQENGFSSHFVSAKTGDSVFLCFQKVAAEILGIKLNKAEIEQSQRVVKADIVNYNQEPMSRTV
NPPRSSMCAVQ
```

Exon 9

```
>NP_004240.2| NM_004249.4:1-220 ras-related protein Rab-28 isoform 2 [Homo
sapiens]220 aa
MSDSEEESQDRQLKIVVLGDGASGKTSLTTCFAQETFGKQYKQTIGLDFFLRRITLPGNLNVTLQIWDIG
GQTIGGKMLDKYIYGAQGVLLVYDITNYQSFENLEDWYTVVKKVSEESETQPLVALVGNKIDLEHMRTIK
PEKHLRFCQENGFSSHFVSAKTGDSVFLCFQKVAAEILGIKLNKAEIEQSQRIVRAEIVKYPEEENQHTT
STQSRICSVQ
```

Exon 8

```
>NP_001153073.1| NM_001159601.2:1-204 ras-related protein Rab-28 isoform 3 [Homo
sapiens]204aa
MSDSEEESQDRQLKIVVLGDGASGKTSLTTCFAQETFGKQYKQTIGLDFFLRRITLPGNLNVTLQIWDIG
GQTIGGKMLDKYIYGAQGVLLVYDITNYQSFENLEDWYTVVKKVSEESETQPLVALVGNKIDLEHMRTIK
PEKHLRFCQENGFSSHFVSAKTGDSVFLCFQKVAAEILGIKLNKAEIEQSQGHFIIFISSTNRE
```

Exon 7

Isoform S (identifier: P51157-1) [UniParc] FASTA Added to basket
Also known as: Rab28S
This isoform has been chosen as the canonical[1] sequence. All positional information in this e
downloadable versions of the entry.
« Hide

```
        10         20         30         40         50
MSDSEEESQD RQLKIVVLGD GASGKTSLTT CFAQETFGKQ YKQTIGLDFF
        60         70         80         90        100
LRRITLPGNL NVTLQIWDIG GQTIGGKMLD KYIYGAQGVL LVYDITNYQS
       110        120        130        140        150
FENLEDWYTV VKKVSEESET QPLVALVGNK IDLEHMRTIK PEKHLRFCQE
       160        170        180        190        200
NGFSSHFVSA KTGDSVFLCF QKVAAEILGI KLNKAEIEQS QRVVKADIVN
       210        220
YNQEPMSRTV NPPRSSMCAV Q
```

Isoform L (identifier: P51157-2) [UniParc] FASTA Added to basket
Also known as: Rab28L
The sequence of this isoform differs from the canonical sequence as follows:
193-221: VVKADIVNYNQEPMSRTVNPPRSSMCAVQ → IVRAEIVKYPEEENQHTTSTQSRICSV
Show »

Isoform 3 (identifier: P51157-3) [UniParc] FASTA Added to basket
The sequence of this isoform differs from the canonical sequence as follows:
192-221: RVVKADIVNYNQEPMSRTVNPPRSSMCAVQ → GHFIIFISSTNRE

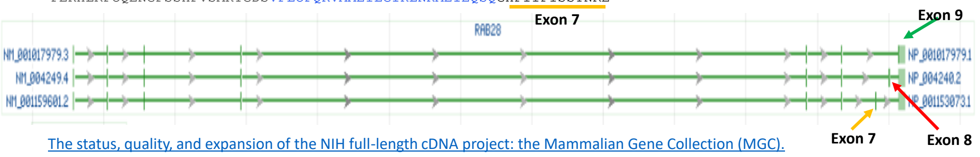

The status, quality, and expansion of the NIH full-length cDNA project: the Mammalian Gene Collection (MGC).
Gerhard DS, et al.
Genome Res. 2004 Oct;14(10B):2121-7. doi: 10.1101/gr.2596504.

Rab35 Rab family

Alternative splicing exon skipping

Isoform 2 NP_001161078 appeared first in jawless fish cyclostomata (lampreys)

```
NP_001161078.1 NM_001167606|:1-152 ras-related protein Rab-35 isoform 2 [Homo
sapiens]
MARDYDHLFKLLIIGDSGVGKSSLLLRFADNTFSGSYITTIGVDFKIRTVEINGE
KVKLQIWDTAGQERFRTITSTYYRGTHGVIVVYDVTSAESFVNVKRWLHEINQNC
DDVCRILDVQLHHGAGPPSKERQPGKTAAATTERCGEAHEEQ

NP_006852.1 NM_006861.7|:1-201 ras-related protein Rab-35 isoform 1 [Homo sapiens]
MARDYDHLFKLLIIGDSGVGKSSLLLRFADNTFSGSYITTIGVDFKIRTVEINGE
KVKLQIWDTAGQERFRTITSTYYRGTHGVIVVYDVTSAESFVNVKRWLHEINQNC
DDVCRILVGNKNDDPERKVVETEDAYKFAGQMGIQLFETSAKENVNVEEMFNCIT
```

Exon 5

```
ELVLRAKKDNLAKQQQQQNDVVKLTKNSKRKKRCC
```

Isoform 1 (identifier: Q15286-1) [UniParc] FASTA Add to basket
This isoform has been chosen as the canonical[1] sequence. All positional information in this entry refers
downloadable versions of the entry.
« Hide

```
        10         20         30         40         50
MARDYDHLFK LLIIGDSGVG KSSLLLRFAD NTFSGSYITT IGVDFKIRTV
        60         70         80         90        100
EINGEKVKLQ IWDTAGQERF RTITSTYYRG THGVIVVYDV TSAESFVNVK
       110        120        130        140        150
RWLHEINQNC DDVCRILVGN KNDDPERKVV ETEDAYKFAG QMGIQLFETS
       160        170        180        190        200
AKENVNVEEM FNCITELVLR AKKDNLAKQQ QQQNDVVKL  TKNSKRKKRC

C
```

Isoform 2 (identifier: Q15286-2) [UniParc] FASTA Add to basket
The sequence of this isoform differs from the canonical sequence as follows:
118-201: VGNKNDDPER...KNSKRKKRCC → DVQLHHGAGPPSKERQPGKTAAATTERCGEAHEEQ
Show »

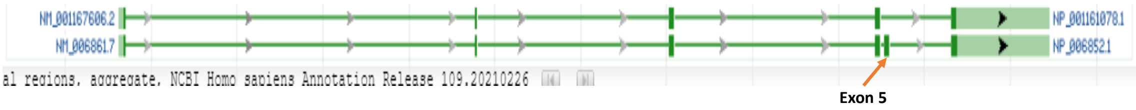

Complete sequencing and characterization of 21,243 full-length human cDNAs.
Ota T, et al.
Nat Genet. 2004 Jan;36(1):40-5. doi: 10.1038/ng1285. Epub 2003 Dec 21

Arl8B/Arl10C/Gie1. Arf family

Alternative splicing exon skipping

Isoform2 Q9NVJ2 appeared first in placental mammals in boreoeutheria

```
NP_060654.1|: Q9NVJ2-1|1-186 ADP-ribosylation factor-like protein 8B [Homo sapiens]
MLALISRLLDWFRSLFWKEEMELTLVGLQYSGKTTFVNVIASGQFSEDMIPTVGFNMRKVTKGNVTIKIWDIGGQPR
FRSMWERYCRGVNAIVYMIDAADREKIEASRNELHNLLDKPQLQGIPVLVLGNKRDLPNALDEKQLIEKMNLSAIQD
REICCYSISCKEKDNIDITLQWLIQHSKSRRS
```

Exon 4 Exon 5 Exon 6

Exon 7

```
Q9NVJ2-2|ARL8B_HUMAN Isoform 2 of ADP-ribosylation factor-like protein 8B OS=Homo
sapiens OX=9606 GN=ARL8B 163aa
MLALISRLLDWFRSLFWKEEMELTLVGLQYSGKTTFVNVIASGQFSEDMIPTVGFNMRKVTKGNVTIKIWDIGGQPR
FRSMWERYCRGVNAIVYMIDAADREKIEASRNELHNLLDKPQLQGIPISHFSGLFSIQNLEEAEASPEVFQSFLAII
LELLSVPLK
```

Exon 4 Exon 7

Isoform 1 (identifier: Q9NVJ2-1) [UniParc] FASTA Add to basket
This isoform has been chosen as the canonical[1] sequence. All positional information in t
downloadable versions of the entry.
« Hide

```
        10         20         30         40         50
MLALISRLLD WFRSLFWKEE MELTLVGLQY SGKTTFVNVI ASGQFSEDMI
        60         70         80         90        100
PTVGFNMRKV TKGNVTIKIW DIGGQPRFRS MWERYCRGVN AIVYMIDAAD
       110        120        130        140        150
REKIEASRNE LHNLLDKPQL QGIPVLVLGN KRDLPNALDE KQLIEKMNLS
       160        170        180
AIQDREICCY SISCKEKDNI DITLQWLIQH SKSRRS
```

Isoform 2 not in NCBI

Isoform 2 (identifier: Q9NVJ2-2) [UniParc] FASTA Add to basket
The sequence of this isoform differs from the canonical sequence as follows:
125-186: VLVLGNKRDL...LIQHSKSRRS → ISHFSGLFSI...ILELLSVPLK

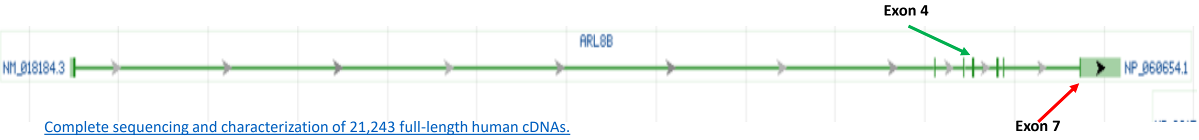

Complete sequencing and characterization of 21,243 full-length human cDNAs.
Ota T, et al.
Nat Genet. 2004 Jan;36(1):40-5. doi: 10.1038/ng1285. Epub 2003 Dec 21

Rab15

Alternative splicing isoform NP_941959, alternative 5 ´splice position exon 4

Isoform NP_941859 appears first in primates

```
>NP_001295083.1|P59190-1 NM_001308154.2 :1-212 ras-related protein Rab-15 isoform 2
[Homo sapiens]
MAKQYDVLFRLLLIGDSGVGKTCLLCRFTDNEFHSSHISTIGVDFKMKTIEVDGIKVRIQIWDTAGQERY
QTITKQYYRRAQGIFLVYDISSERSYQHIMKWVSDVDEYAPEGVQKILIGNKADEEQKRQVGREQGQQLA
KEYGMDFYETSACTNLNIKESFTRLTELVLQAHRKELEGLRMRASNELALAELEEEEGKPEGPANSSKTC
WC
>NP_941959.1|P59190-2 NM_198686.3:1-208 ras-related protein Rab-15 isoform 1 [Homo
sapiens]
MAKQYDVLFRLLLIGDSGVGKTCLLCRFTDNEFHSSHISTIGVDFKMKTIEVDGIKVRIQIWDTAGQERY
QTITKQYYRRAQGIFLVYDISSERSYQHIMKWVSDVDEVGDATSLPGCGEGASPGKARRGPDGKANASRK
LCLPQPWMKTSGTHQKASRRSLLGIRLMRSRNGRWEESKGSSWRRSMAWTSMKQVPAPTSTLKSHSRV
```

Exon 4

alternative 5´splice exon 4

Isoform 1 (identifier: **P59190-1**) [UniParc] FASTA Add to basket

This isoform has been chosen as the canonical[i] sequence. All positional inform
downloadable versions of the entry.

« Hide

```
        10         20         30         40         50
MAKQYDVLFR LLLIGDSGVG KTCLLCRFTD NEFHSSHIST IGVDFKMKTI
        60         70         80         90        100
EVDGIKVRIQ IWDTAGQERY QTITKQYYRR AQGIFLVYDI SSERSYQHIM
       110        120        130        140        150
KWVSDVDEYA PEGVQKILIG NKADEEQKRQ VGREQGQQLA KEYGMDFYET
       160        170        180        190        200
SACTNLNIKE SFTRLTELVL QAHRKELEGL RMRASNELAL AELEEEEGKP
       210
EGPANSSKTC WC
```

Isoform 2 (identifier: **P59190-2**) [UniParc] FASTA Add to basket

The sequence of this isoform differs from the canonical sequence as follows:

109-212: YAPEGVQKIL...PANSSKTCWC → VGDATSLPGC...TSTLKSHSRV

Rab15 alternative splicing is altered in spheres of neuroblastoma cells.
Pham TV, et al.
Oncol Rep. 2012 Jun;27(6):2045-9. doi: 10.3892/or.2012.1731. Epub 2012 Mar 15.

Suppl. Fig. 9
