# Supplementary material for: Origin and Evolution of RAS Oncoprotein Membrane Targeting
Source: Res Sq. 2023 Jan 20:rs.3.rs-2485219. Preprint. [Version 1] doi: 10.21203/rs.3.rs-2485219/v1 (PMC9882654; doi:10.21203/rs.3.rs-2485219/v1)
Supplement: Suppl. Fig 1 — Suppl. Fig. 1. Lancelet2 has distinct gene structure and HVR characteristics. Clustalw MAFFT alignment of lancelet1 and lancelet2 cephalochordate sequences. Exons are depicted in alternate black and blue letters. Whereas lancelet1 has homolog introns a-c like vertebrate oncogenes and intron d like other invertebrates, lancelet2 has a single intron (indicated by a red arrow). The dicysteine motif that includes the CaaX cysteine of Lancelet2 (highlighted in orange) is also different from other oncogenes. Amino acid identity: identical (*); strongly similar (:); weakly similar (.). [file Suppl.Fig.1_10.1.2023.pdf]

```
                                                    a                          d                               b
lancelet1_Bf   MTEYKLVVVGDGGVGKSALTIQLIQNHFVQEYDPTIEDSYRKQVVIDGETCLLDILDTAGQEEYSAMRDQYMRTGEGFLCVFAINNDKSFEDIAMYRE
lancelet2_Bf   MTEYKLVVVGAGGVGKSALTIQLIQNHFVDEYDPTIEDSYRKQVVIDGETCLLDILDTAGQEEYSAMRDQYMRTGEGFLCVFALNNTKSFEDISMYRE
               ********** ******************:****************************************************:** ******:****

                                                                     c
lancelet1_Bf   QIKRVKDSDEVPIVLVGNKCDLP-ARTVENKQAWTLAKSYGVP-YVETSAKTRMGVDDAFYTLVREIRAYKERQGKAGKK-KKKNFKSRCTIL
lancelet2_Bf   QIKRVKDSEQVPMVLVGNKADLPQRVIDNKQAQDMAKSYSIPYVETSAKTRLGVEDAFYTLVREIRMDKANRQATKRQGK--DGTSGTCCVLL
               ********::**:******.*** *.::****  :****.:**********:**:***********   *  .:   :  ::  *..  ..  *  :*
```

Suppl. Fig. 1
